# Using immersive virtual reality to recreate the synaesthetic experience

**DOI:** 10.1177/20416695231166305

**Published:** 2023-09-04

**Authors:** Rebecca Taylor, Sarune Savickaite, Susanna Henderson, David Simmons

**Affiliations:** School of Psychology and Neuroscience, University of Glasgow, Glasgow, UK; School of Education, University of Glasgow, Glasgow, UK; School of Psychology and Neuroscience, University of Glasgow, Glasgow, UK

**Keywords:** synaesthesia, virtual reality, perception, cross-modal correspondences

## Abstract

Synaesthesia is a condition where people experience unusual sensory or cognitive sensations in response to apparently unrelated stimuli. This paper presents two experiments which aimed to examine whether Virtual Reality (VR) technology can be used to recreate the synaesthetic experience. There is a lack of research in this area, with most studies focussing primarily on synaesthetic colors. Experiment 1 aimed to build on previous research by using not only a traditional color-picker but also VR to capture a more nuanced picture of synaesthetic perception. A multiple case study design was used to examine the experiences of six participants in detail. Data gathering took place via Zoom. During the initial data-gathering session, participants used a color-picker to provide grapheme-color associations. After this session, some of the participants’ synaesthetic experiences were recreated using a VR-by-proxy approach. Results indicated that VR is capable of capturing elements of synaesthetic perception that other methods have been unable to, such as texture, small degrees of movement, and 3D structure. Experiment 2 expanded upon these findings by moving beyond the VR-by-proxy approach and asking three participants to recreate their own audiovisual synaesthetic associations in the VR environment. Inductive Thematic Analysis was used to analyze the results of this experiment. The potential of expanding this technique to other forms of perceptual diversity is discussed.

Synaesthesia is a condition where people experience unusual sensory or cognitive sensations in response to apparently unrelated stimuli (Simner, 2011). For example, hearing speech or music may trigger the experience of colors or tastes, or letters may be perceived as inherently colored. Estimated prevalence in the general population has varied from as low as 0.5% (Baron-Cohen et al., 1996) to as high as 4.4% (Simner et al., 2006). Current estimates assume no strong sex bias—although previous thinking was that it was more common in women, this was likely due to differences in self-referral (Simner & Carmichael, 2015).

Stimuli giving rise to synaesthetic experiences are known as “inducers,” and the resultant perceptual or cognitive experiences are known as “concurrents” (Grossenbacher, 1997). Inducers and concurrents may be in the same or different modalities (Rothen et al., 2013). Convention dictates that synaesthetic variants are denoted in the inducer–concurrent format, a practice that will be followed in this report. Some synaesthetes experience their concurrents as being spatially extended (Simner, 2011), for example, those with grapheme-color synaesthesia may see the elicited colors as projected on the text they are reading. These synaesthetes are known as “projectors.” Others simply have knowledge of their concurrent or see it “in the mind's eye” and are known as “associators” (Ward & Simner, 2020).

Synaesthesia can be developmental, acquired, or induced. This study will focus on developmental synaesthesia only, which is synaesthesia that has been present from a young age, and accounts for most known cases of synaesthesia (Ward & Simner, 2020).

## Types of Synaesthesia

Over 100 variants of synaesthesia have been reported to date (Ward & Simner, 2020). Synaesthetes often display more than one type of synaesthesia (Ward, 2019). Colored days are the most common known variant, experienced by 64% of synaesthetes (Simner et al., 2006). Cognitive constructs such as graphemes or words represent the majority of inducers, accounting for 88%, while color is by far the most common concurrent, accounting for 95% of all elicited experiences. This study will focus primarily on grapheme-color and auditory-visual synaesthesia.

Grapheme-color synaesthesia is the most commonly studied variant, accounting for around 45% of synaesthetes, or 1.4% of the total population (Simner et al., 2006). In grapheme-color synaesthesia, letters are perceived as being inherently colored. Colors will be individual to each synaesthete, although certain graphemes have a higher-than-average chance of being a particular color—for example, A is red and D is brown more often than would be expected by chance (Rich et al., 2005; Simner et al., 2005).

In auditory-visual synaesthesia, additional visual content is triggered by hearing sounds (Neufield et al., 2012). Throughout the literature, additional color experiences are heavily focussed on; however, any visual quality such as shape (Chiou et al., 2013), texture, and movement (Moos et al., 2013; Eagleman & Goodale, 2009) may be elicited. Additionally, concurrents may be experienced in three dimensions (Eagleman & Goodale, 2009). The automatic visual content experienced by auditory-visual synaesthetes often shows similarities with general cross-modal associations that the nonsynaesthetic population have between sound and vision, such as high-pitched tones appearing higher in space and being smaller, less rounded, and lighter in color than lower-pitched tones (Ward et al., 2006). These cross-modal correspondences are sometimes termed “synaesthetic congruences” and are thought to play a role in information processing (Spence, 2011).

## Recreating Synaesthetic Experiences

Many studies have depicted synaesthetes’ grapheme-color associations using color pickers. However, these are generally used as a method of assessing the genuineness of grapheme-color synaesthesia (Rothen et al., 2013), rather than portraying all concurrent phenomenology. Chiou and colleagues expanded upon this, using the software GIMP in conjunction with hand drawings, verbal descriptions, and Adobe Photoshop (1990) to recreate the concurrent experiences of synaesthetes who experienced colored geometric shapes in particular locations in space in response to sounds (Chiou et al., 2013). Relatedly, a professional animator created animated reconstructions of auditory-visual synaesthesia, which were later used in an experiment examining whether non-synaesthetes prefer these to control animations (Ward et al., 2008).

More broadly, many artists have created works based upon synaesthetic associations. Artists such as Mikalojus Konstantinas Čiurlionis and Wassily Kandinsky created the pieces *Sonata of the Stars* (1908) and *Fuga (Fugue)* (1914), respectively, which both expressly aimed to translate music into paintings (Ward et al., 2008). More recently, artists Melissa McCracken and Jack Coulter are known for paintings of their auditory-visual synaesthesia, with McCracken producing oil paintings of popular songs and Coulter creating a depiction of Glastonbury Festival 2016, painted after listening to every performance at the event (Taggart, 2019). Relatedly, the artist Carol Steen created the painting “Vision,” which depicts her experience of tactile-visual synaesthesia during an acupuncture session (Steen, 2001). Steen explains how she was inspired to create “Vision” due to her inability to adequately describe the nature of her experiences with words (Steen, 2001). More recently, the musician Billie Eilish has recreated her own synaesthetic experiences of the songs in her debut album using art installations (Billie Eilish Experience, 2019) (see https://web.archive.org/web/20190403020359/billieeilishexperience.com/).

Clearly, synaesthetic experiences are more multifaceted than can be captured by color pickers alone, and several studies, in addition to the work of synaesthetic artists, reflect this. This paper aims to build upon these previous works, in particular the work of Ward et al. (2008), by presenting an exploratory feasibility study in which synaesthetic associations are recreated in a Virtual Reality (VR) environment using the art package OpenBrush (2016)^1^. Openbrush (2016) allows users to paint in an immersive 3D environment with a range of brushes including dynamic and textured effects. As a medium, VR is more accessible in terms of the time and skill required to create sketches compared to animation packages. Furthermore, VR is a highly authentic and realistic tool with strong ecological validity (Savickaite et al., 2021), and, due to its immersive nature, allows users other than the creator to “step in” to the 3D environment and experience it. Therefore, the purpose of this paper is to introduce VR as a new method of exploring the phenomenology of synaesthesia with the potential of sharing this with nonsynaesthetes.

The only study which has utilized VR technology as a means of recreating synaesthesia focussed on spatial sequence synaesthesia only. In this study, the authors used WorldViz VR software to allow participants to place items from common sequences (such as the months of the year) in 3D space, corresponding to how they experience these sequences (Eagleman, 2009). This was nonimmersive, with an avatar representing the participants. Additionally, a recent project created VR musical visualizations drawing on the concept of synaesthetic associations but did not appear to work with synaesthetes to achieve this (Weinel, 2021). Recent related studies have, however, focussed on the application of VR technology in areas such as art therapy (Hacmun et al., 2018) and visual processing styles in relation to autistic and ADHD traits (Savickaite et al., 2021).

This paper presents two experiments. The first experiment is a pilot study discussing the cases of six synaesthetes who report various combinations of grapheme-color and auditory-visual synaesthesia. We decided to carry this research out with a small group of participants in the form of multiple case studies in order to garner in-depth, detailed accounts and representations of their experiences, which is a benefit of qualitative research (Queiros et al., 2017).

Due to COVID-19, in Experiment 1, the researcher acted as an intermediary (“VR-by-proxy”). To this end, individual Zoom interviews were conducted with each participant, where we gathered detailed descriptions of their synaesthetic experiences in response to a range of stimuli. We used Google Slides to present stimuli, collect data, and recreate color associations via a color-picker. In cases where shape, movement, textural or three-dimensional qualities were present in the concurrent, we attempted to recreate the participant's experience in OpenBrush (2016). In this way, we aimed to provide recreations of synaesthetic experiences using both a traditional color-picker and OpenBrush (2016), and to ascertain whether OpenBrush (2016) can capture some elements of the synaesthetic experience in a way that traditional methods cannot—and by extension, whether it is worthwhile pursuing VR further as a means of exploring synaesthesia.

In Experiment 2, we followed a similar procedure to Experiment 1, but this time (post-pandemic) participants attended experimental sessions in person and were able to draw their experiences in OpenBrush (2016). Again, a multiple case study design was used to explore a small number of participants’ experiences in detail. Inductive Thematic Analysis (ITA) was used for the analysis of participants’ descriptions of their experiences.

## Experiment 1

### Method

#### Participants

Six participants were recruited for the study through university channels, personal connections, and the UK Synaesthesia Association's mailing list. Inclusion criteria were a history of developmental synaesthesia and proficiency in English. Three of the participants were men and three were women, with ages ranging from 20–59.

#### Apparatus

OpenBrush (2016) was accessed via PlayStation VR on a Playstation 4 slim, CUH-ZVR2 model. This is a fully immersive, head-mounted display (Mandal, 2013) with six degrees of freedom (Melatti & Johnsen, 2017), a 90-hz refresh rate, 5.7-inch OLED display with 100 degrees field of view. Headset dimensions were approximately 187 × 185 × 277 mm.

#### Updated Screening Questionnaire

A short screening questionnaire was used to establish which types of synaesthesia participants experienced. This questionnaire was a condensed version of one previously available on the University of Sussex's website (for an update version see https://www.syntoolkit.org/studies/syn-database-volunteer/start). Questions 9, 11, 15, 16, and 17 were retained from the original survey. Participants were first asked to complete a grid that allowed them to match a range of triggering stimuli with potential synaesthetic experiences. Next, they were asked whether they experience letters, numbers, days, or months as having genders or personalities. The next two questions asked whether they had experienced these types of synaesthesia for as long as they can remember and whether their associations ever change. Finally, the last question asked whether there was anything else that the participants would like to say about their synaesthesia.

#### Stimuli

Stimuli were tailored to each participant based on their answers to the questionnaire. Stimuli (presented visually) included letters of the alphabet, numbers 0–9, days of the week, and months of the year, all presented in Arial font. Auditory stimuli included a selection of everyday sounds and musical notes (C_4_, D_4_, E_4_, F_4_, G_4_, A_4_, B_4_ played on piano; E_2_, F_2_, G_2_, A_2_, B_2_, C_3_, D_3_ played on a guitar), a selection of short piano melodies, and various voices, celebrity, and noncelebrity, saying single words or short phrases. All participants were played an equal ratio of male and female voices. Piano melodies ranged in duration from 3–13 s and were unfamiliar to the participants. Music and noises were obtained from the website Freesound (https://freesound.org/). Voice clips were primarily sourced from sound files provided by Mahrholz et al. (2018), while some celebrity voice clips were found on YouTube (https://www.youtube.com/). Celebrity voice clips used were of Queen Elizabeth II and Barack Obama available under Creative Commons licenses.

**Figure 1.**
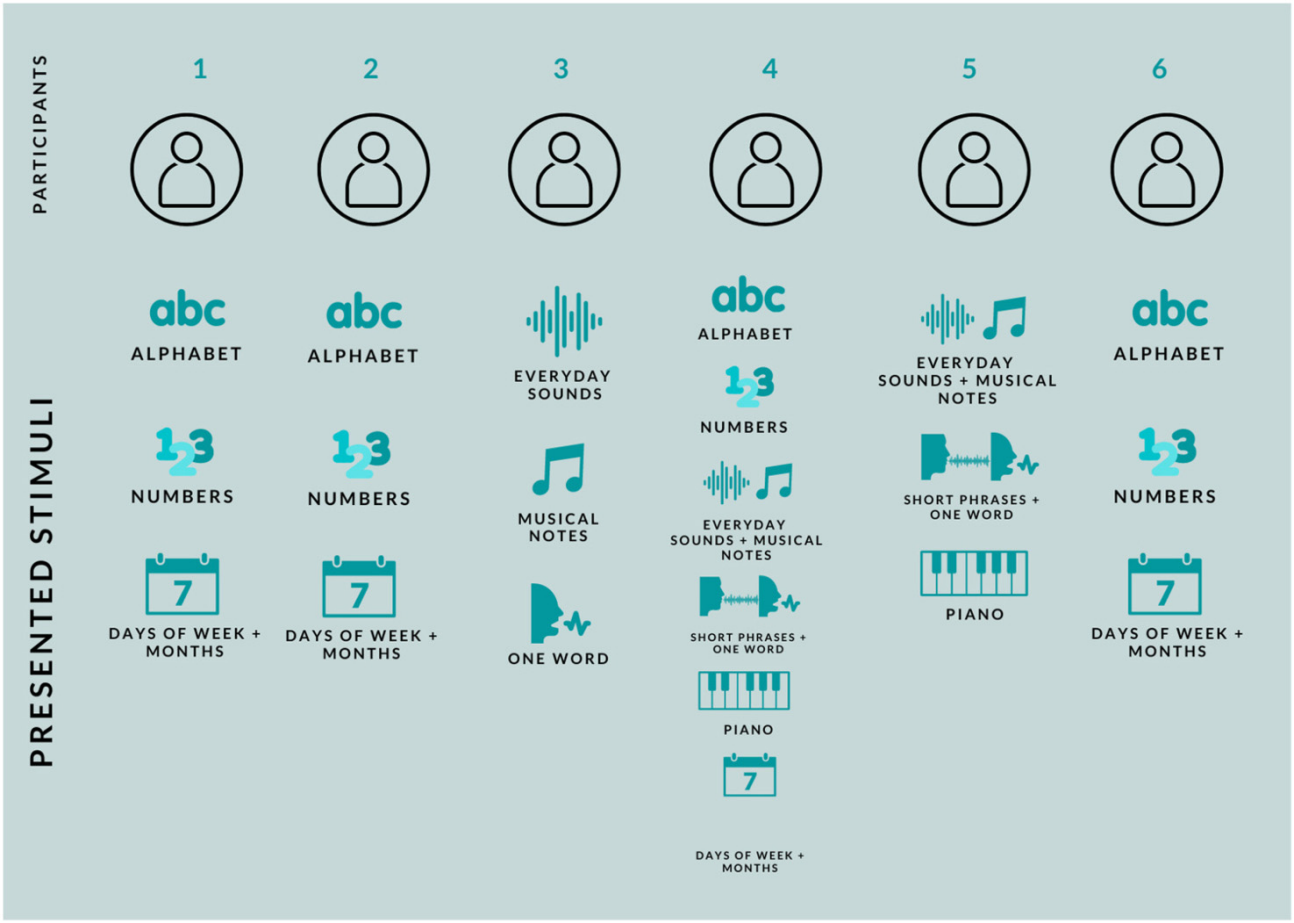
Summary of the stimuli presented to each participant.

Sound files were downloaded from their various sources then converted to either WAV or MP3 files via the program Audacity (The Audacity Team, 2000). Clips sourced from YouTube were trimmed down to the required length (e.g., the word “hello” was extracted from a clip of a speech) also via Audacity. One participant reported that she experienced differing concurrents based on the volume of a sound. In this participant's case, volume was manipulated simply by playing each sound file twice, once at full volume on the researcher's laptop and once at half volume^2^. See Figure 1 for details of the stimuli presented to each participant.

#### Procedure

**Figure 2.**
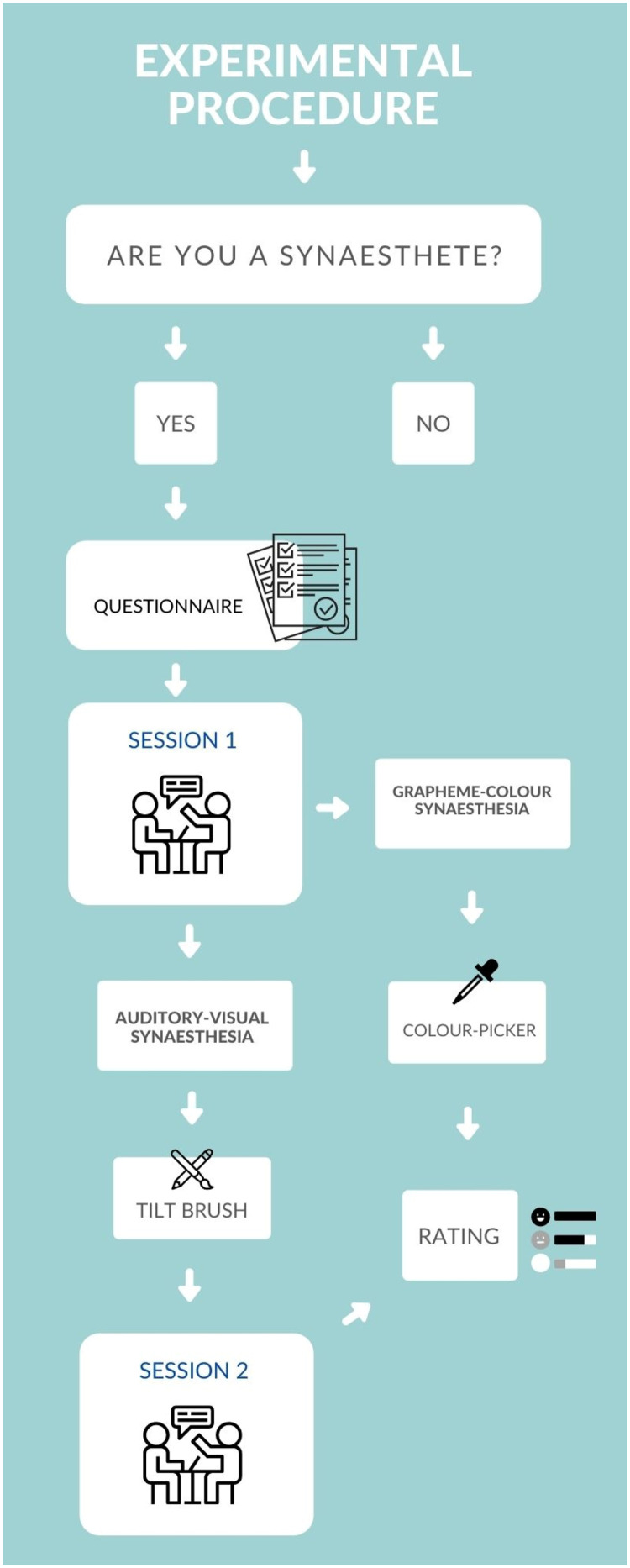
Flowchart illustrating the experimental procedure.

A full flowchart of the experimental procedure is presented in Figure 2. The current experiment was approved by the College of Science and Engineering, University of Glasgow Ethics committee.

#### Questionnaire

First, participants completed a short questionnaire aiming to establish which types of synaesthesia they experienced and so the following sessions could be tailored for each individual participant.

#### Session 1

Next, participants were invited to a Zoom (Zoom Video Communications, 2012) call with the researcher. During this call, participants were presented with stimuli that had been prepared in advance based on their responses to the questionnaire. As we were interested in gathering as much detail as possible about participants’ phenomenological experiences, the viewing time of stimuli was not limited. These stimuli were presented in a Google Slides (Google LLC, 2006) presentation, which was shared at the beginning of the meeting. Both the researcher and the participant had access to this and could edit it by adding descriptions or colors. Where complex shapes were described, sketches were made using pen and paper. At the end of the interview, the researcher showed participants a short video introducing them to OpenBrush (2016). This video is available in the supplementary materials. The purpose of this was to familiarize the participants with OpenBrush (2016) and ascertain whether they thought this medium could be used to display their synaesthetic experiences.

Participants who experienced grapheme-color synaesthesia changed the colors of stimuli themselves using the color-picker on the shared Google Slides presentation and provided ratings for how well this matched their synaesthetic experiences during this initial session. Color data were stored as hexadecimal color codes (HEX). Ratings were measured on a continuous scale from 0–5, with 0 indicating “no match” and 5 indicating “perfect match.”

#### OpenBrush: VR Recreations

After the meeting, the researcher identified which stimuli had been sufficiently captured on slides and which would benefit from further exploration in OpenBrush (2016). For example, if an individual experienced the letter A as brick red and had changed the color of the A in the slideshow to reflect this, it would suffice to leave it at that. However, if a participant viewed a sound as a shimmering of colors around their periphery, the researcher would attempt to implement this in OpenBrush (2016). OpenBrush (2016) illustrations were captured as videos which were edited in Adobe Premier Pro (Adobe Inc, 2003). Ultimately, three of the six participants’ experiences were recreated in this manner.

#### Session 2

Finally, a second meeting was arranged with three participants whose concurrents were further explored. In this meeting, the completed short videos, made by the experimenter using OpenBrush (2016), were shown. Participants were asked to give these an accuracy rating from 0–5 and were also asked to comment on whether VR had any merit in capturing their synaesthetic experiences. These comments were not formally recorded; instead, only the main points were extracted and paraphrased. The full details of the questions and comments are available in Supplementary Material.

### Results

#### Screening Questionnaire

The questionnaire revealed that Participants 1, 2, 4, and 6 experience grapheme-color synaesthesia. Participants 3, 4, and 5 experience auditory-visual synaesthesia, with all three reporting that voices, music, and noises trigger colors and shapes. Participant 4 also reported texture associations for all inducing stimuli. Participants 1 and 2 also reported experiencing ordinal-linguistic personification. Participants 1, 4, and 5 reported that their synaesthetic associations never change, while Participants 2 and 6 reported that most associations never change. Only Participant 3 reported associations that frequently change. All six participants reported experiencing these types of synaesthesia for as long as they can remember.

#### Synaesthetic Recreations

The full results tables detailing participants’ responses to all presented stimuli can be found in the supplementary online materials. In this section, only synaesthetic experiences which were visualized are reported. Using a color picker, Participants 2, 4, and 6 recreated their grapheme-color associations. Participant 1 also used a color picker, in addition to making formatting changes to best capture their experiences. As Participant 2 described some complex color interactions, as well as associated shapes, some of their grapheme-color associations were recreated in OpenBrush (2016). Participants 3 and 4 described complex visual experiences in response to a variety of stimuli. These descriptions were also recreated in OpenBrush (2016). Participant 5 also described visual experiences in response to a range of auditory stimuli, but as these were simple two-dimensional shapes with no movement it was deemed unnecessary to implement these in a three-dimensional environment. Participant 6 also described shapes associated with the days of the week and months of the year, but these were also simple two-dimensional shapes, so again it was decided that it was not necessary to implement these in OpenBrush (2016).

#### Alphabet

Table 1 depicts Participant 1, 2, 4, and 6's grapheme-color associations for the alphabet, as chosen on a color-picker. Associated textures, when they were experienced, are also reported. Most letters tended to be the same color for each participant whether they were in uppercase or lowercase, but where there are differences, these are reported. Additionally, Participant 2 experienced some letters as changing colors in certain words.

**Table 1.**
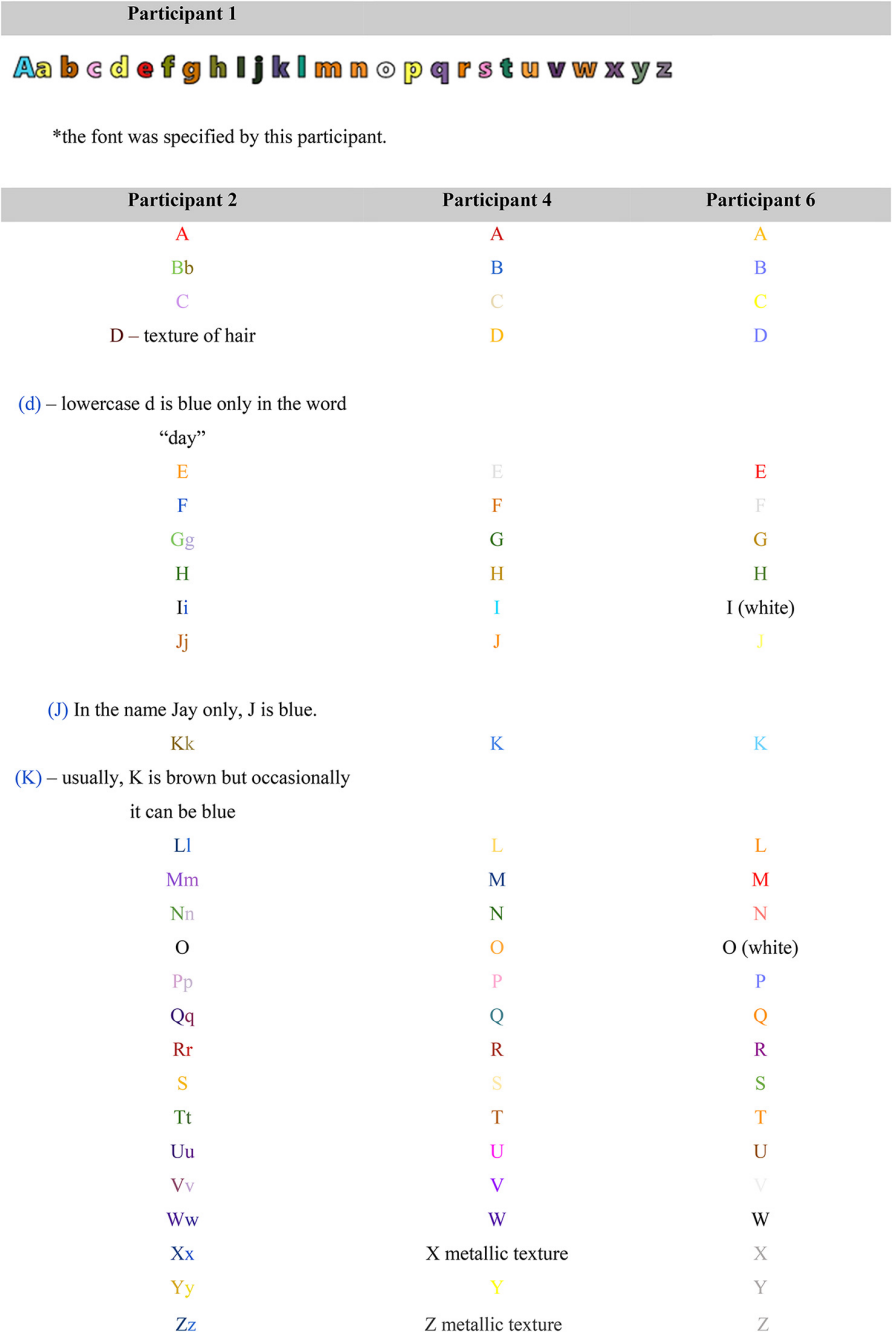
Grapheme-Color Associations for the Alphabet.

#### Numbers

**Figure 3.**
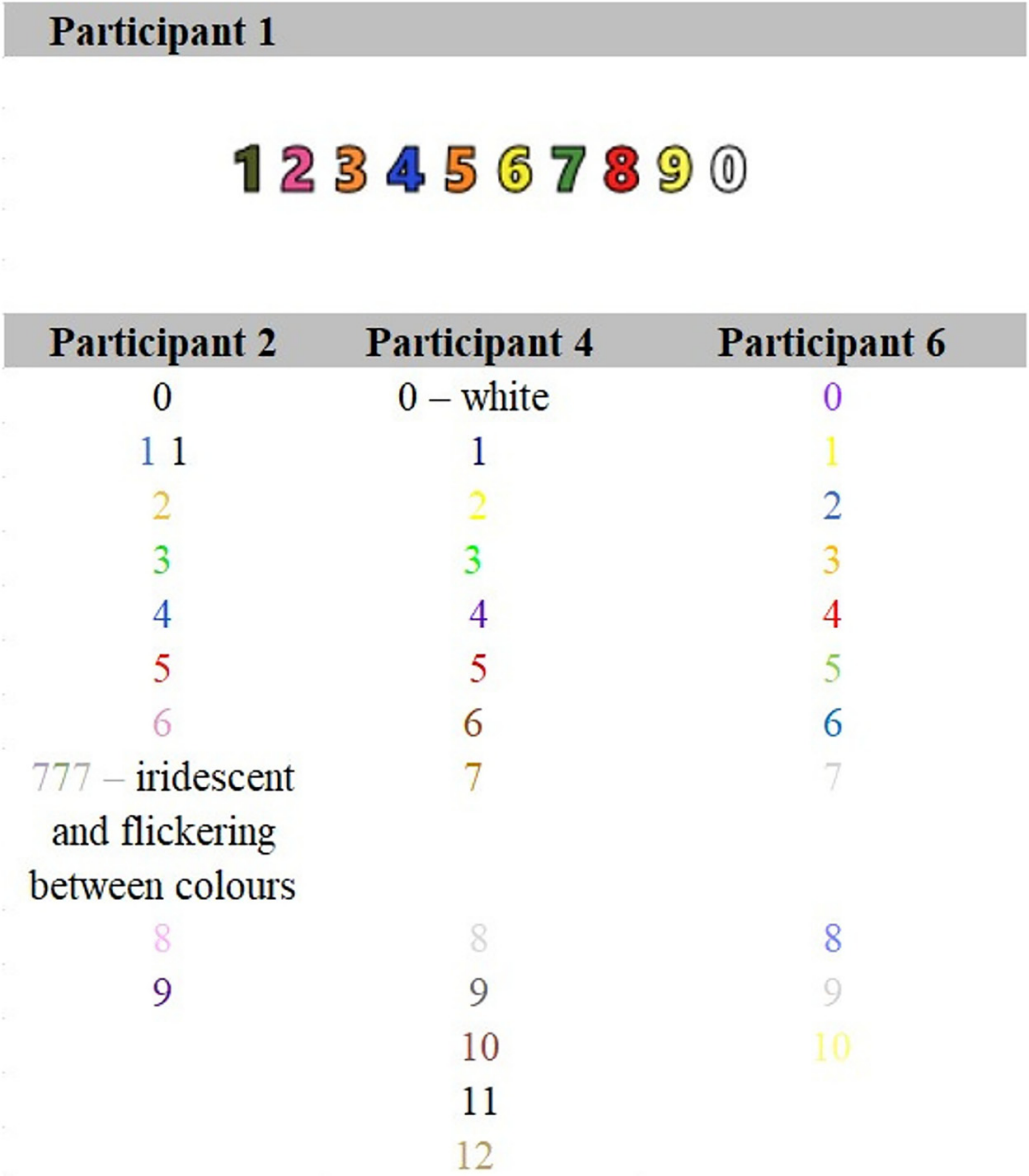
Grapheme-color associations for numbers.

For most participants, the digits 0–9 are colored and multiple-digit numbers become a combination of the digits within. For Participant 6, this is the case after the number 10, and for Participant 4, after the number 12. Therefore, Figure 3 reports color associations for the numbers 0–9, 0–9, 0–12, and 0–10 for Participants 1, 2, 4, and 6, respectively.

#### Days of the Week

**Figure 4.**
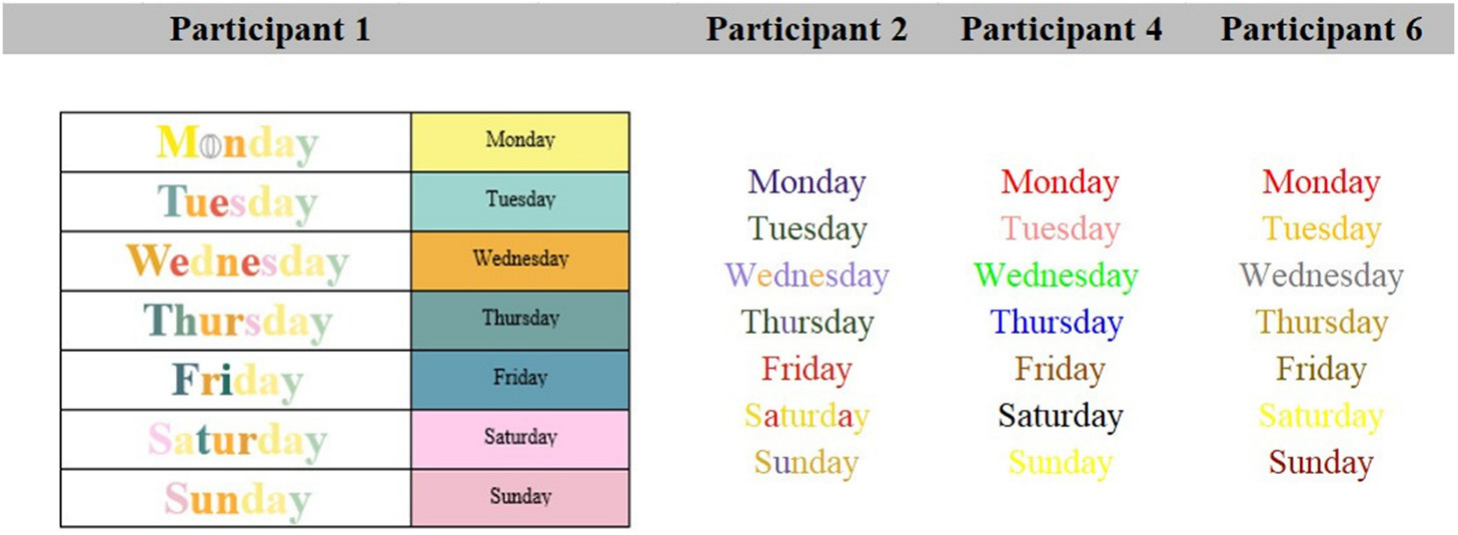
Grapheme-color associations for days.

Participants 4 and 6 experience the days of the week as one color. Participant 2 also experiences a dominant color, but occasionally the colors of some individual letters shine through. Participant 1 reported experiencing both individual color associations and overall dominant color. For clarity, these are displayed in separate columns (Figure 4).

#### Months of the Year

**Figure 5.**
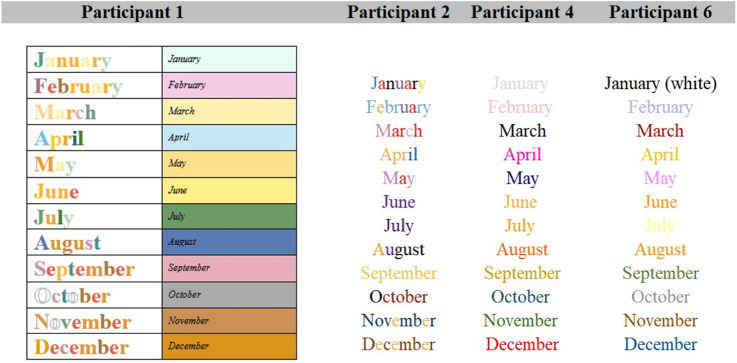
Grapheme-color associations for months.

Participant 2 experiences the months of the year as combinations of the letters within, while Participants 4 and 6 experience one dominant color association (Figure 5). Again, Participant 1 reported experiencing both individual color associations and overall dominant color.

#### Ratings

The ratings that each participant gave the recreations documented above, out of 5, are reported below. Ratings are not intended for formal statistical analysis and are aimed at understanding how representative the drawings/recreations appear to the participants (Table 2). Note that they are almost all high ratings.

**Table 2.** Accuracy Ratings for Grapheme-Color Recreations on a Continuous Scale 0 (Least Accurate) to 5 (Most Accurate).

	Participant 1	Participant 2	Participant 4	Participant 6
Letters	4.5	4.5	4.5	5
Numbers	4.5	3.5	5	5
Days of the Week	4.5	4	5	5
Months of the Year	4.5	3.5	4.5	5

#### OpenBrush: VR Recreations

**Figure 6.**
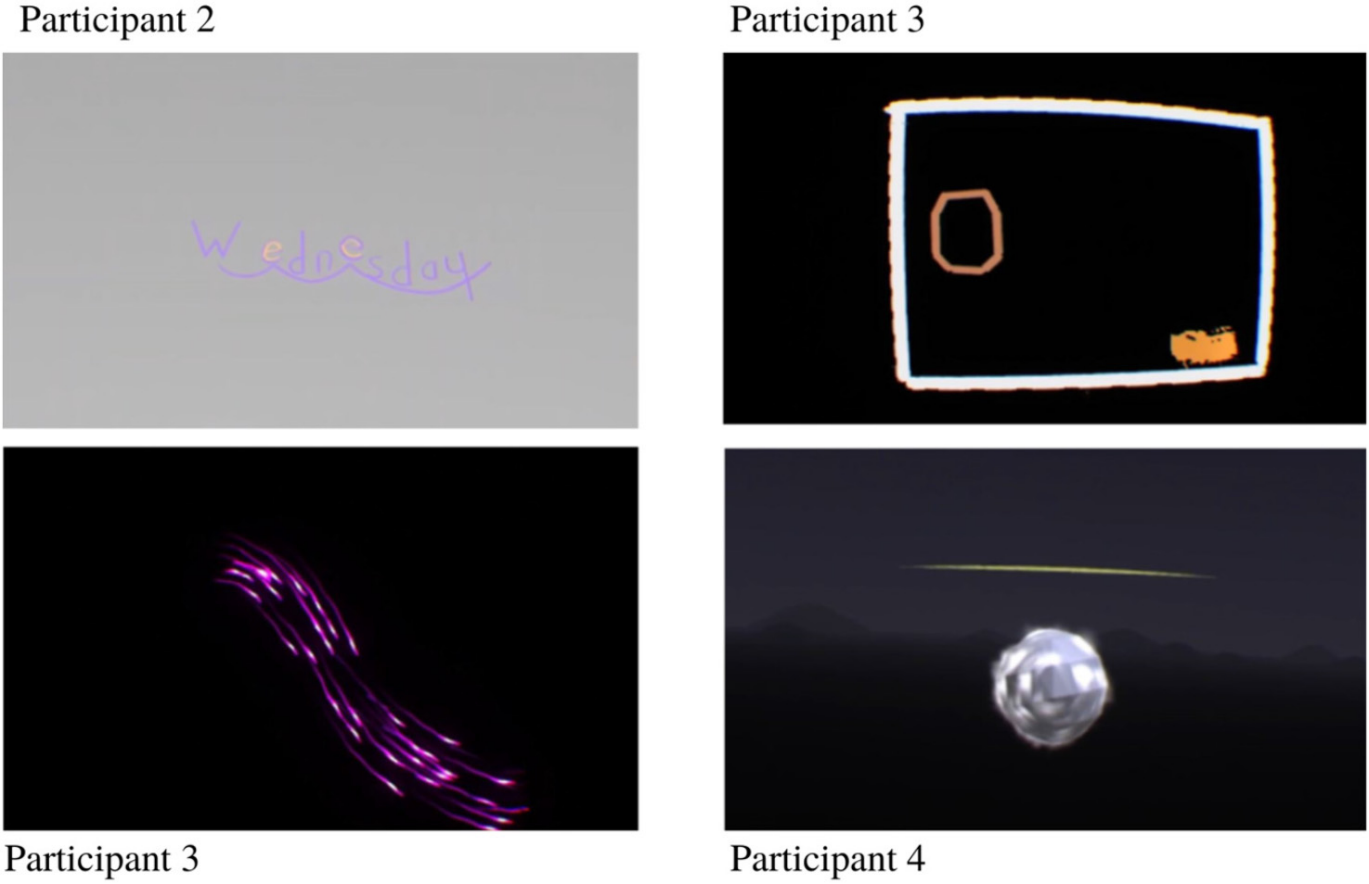
Example screenshots of OpenBrush (2016) videos created by the researcher, using participants’ descriptions.

This section concerns the synaesthetic experiences which were recreated in OpenBrush (2016). Participants’ ratings of each depiction are provided, as well as their general thoughts on each image and on OpenBrush (2016) as a medium. Example screenshots of OpenBrush (2016) videos generated by the researcher are presented in Figure 6. All videos can be found in the supplementary online materials.

#### Participant 2

In addition to colors, Participant 2 reported associated shapes for the days of the week and months of the year. These shapes reflect the attention he gives to each part of the word as he scans it. When presented with the OpenBrush (2016) videos which depict these shapes in addition to colors, Participant 2 reported that they were good schematics, but in most cases did not accurately reflect how he truly sees them, but rather how much focus he gives each letter. These shapes would be better used as indicators of how bold the letters should be. Full ratings and descriptions are presented in Table 3.

**Table 3.** Participant 2 OpenBrush (2016) Feedback.

Stimuli	Ratings and Feedback
7	4.5 of 5
	The participant said that this depiction is the most accurate he has seen. The overlay of colors captures what he had difficulty describing well. However, a vaguely indistinct nature is at the core of the letter 7, so seeing it so clearly took away from the overall accuracy.
Monday	2 of 5
	The slanting is distracting, rather the end of the word should fade out
Tuesday	2 of 5
	The u and e should be like the e on Wednesday, with the individual letter's colors overlaid on the background color
Wednesday	3.5 of 5
	Generally good
Thursday	3/5 The u is both purple and yellow, which is difficult to both explain and capture
Friday	2 of 5
	Should be redder
Saturday	3.5 of 5
	Generally good, a yellow outline around the “A”s would be better
Sunday	3 of 5
	The u is both purple and yellow, which is difficult to both explain and capture
January	4 of 5
	This would be more accurate if there was no line underneath but the letters were still in the same position
February	3.5 of 5
	Too much movement up and down
March	4 of 5
	The M and C should be softer
April	3.5 of 5
	Colors should be more mottled. The end of the word is very blue only if he thinks of the end alone.
May	4 of 5
	This time the soft line underneath helped as you can’t take it away and still get the same impression
June	4.5 of 5
	Base purple color very accurate, and the active smoke effects capture the idea of a sunset coming through clouds well
July	2.5 of 5
	Color is too gray (partly due to screen)
August	3 of 5
	Again, the line is helpful here. However, A should be a deeper color
September	3 of 5
	The simplicity of September is captured but the color is too bright
October	3.5 of 5
November	3.5 of 5
	“Nov” section could be darker
December	4 of 5 Generally good

Overall, he did not believe that VR was particularly effective at capturing his synaesthesia in a way that simply changing the colors of letters could not, with exceptions for the number 7 and June. For increased accuracy, he reported, the background should be darker; however, this was difficult to share with the participant, as when the VR environment was reduced to videos, dark colors did not show up well. The participant did express interest in VR if he could use it himself, as he believes it could replicate the feeling of being “alone in his head” better than videos. His biggest issue with any art-based medium was that it is impossible to separate his two types of synaesthesia—grapheme-color and ordinal-linguistic personification—therefore, it is not always possible to capture what feels most important about a word, as it may be related to gender or personality.

#### Participant 3

Overall, the participant expressed surprise at how much of her experience was accurately recreated in VR. However, other aspects were impossible to capture, particularly with regard to certain types of motion. She also noted that her synaesthesia is very difficult to explain, so there would always be a loss in accuracy when another person attempts to reproduce it. Full ratings and descriptions are presented in Table 4.

**Table 4.** Participant 3 OpenBrush (2016) Feedback.

Stimuli	Description of Experience	Ratings and Feedback
Researcher's voice	Bottle green and lemon yellow, like a vignette around a photograph. Any time researcher said “em” there was a flash of lime green in the top right-hand corner.	2 of 5
		Colors are relatively accurate but should be a lot gentler. The frame was drawn very definitively, but really there is no end to the image, it fades out into the periphery.
Birdsong	Initially, this was seen as an orangey color “jiggling” back and forth in the bottom right corner. This then jumped across to middle left and became a static peach color, which was octagonal and twisted as the chirps changed.	Bottom right: 5 of 5
		Although the shape isn’t quite accurate and the colors a little too solid, the movement and rough edges are very accurate. The participant expressed surprise at how well this captured her experience.
		Middle left (peach octagon): 0/5.
		No movement from right to left, and overall, very inaccurate depiction.
Cars: full volume and half	This elicited a pink waterfall starting at ¾ way up on the left-hand side. Just below halfway, purple shards were seen. At half volume, the shards were seen as bubbles instead. Where the bubbles began, the waterfall began falling straight rather than continuing on its path.	4/5 for both depictions. The color is not quite right, but the movement is good (the way it falls in different places is also a good representation of how speech patterns can be, particularly for people with lyrical voices)
White noise	This sound elicited white waves similar to that of a heart rate monitor. The waves were very close together and left a trace of around 10% as they moved from left to right across her visual field.	3 of 5
	When played twice with a gap in between, the wave went back to the start after the short gap. When played on a loop, the wave got stuck at the end after about 13–14s.	The movement and fading out are very important aspects of this sound, and this was not captured. Also, the waves needed to be more angular.
Phone	A teal aura was experienced all over her visual field	5 of 5
		Good because of the transparency. The subtle motion is good but it should be more of a pulsing motion with each ring.

#### Participant 4

Participant 4 reacted positively to the VR environment, stating that it looked promising and expressing interest in using it himself. Overall, he appreciated the 3D element and how it was possible to move around and interact within the environment. Full ratings and descriptions are presented in Table 5.

**Table 5.** Participant 4 OpenBrush (2016) Feedback.

Stimuli	Description of Experience	Ratings and Feedback
Piano note	All musical notes on the piano take the same form. They move upwards or downwards on the y-axis depending on pitch. The shape is a 3D, smooth-edged triangle. It is a creamy pinkish-orange color, with a texture like cheap plastic. The edges are slightly darker.	2.5 of 5
		The color is good, but the shape should be more rounded, and it is difficult to tell if the plastic texture has been captured properly.
Birdsong	This elicited sparkly speckles in the top center. These were a blue and creamy color, tiny and not particularly vivid.	5 of 5
		Two perspectives were shown here, one from a distance and one closer up. From a distance, the participant said it was very close to how he experiences it. The close-up was harder to judge as he does not experience it that way.
Rain	A misty metallic gray mass was seen in the middle, with little bumps protruding outwards. These bumps were not static, they moved slightly.	3 of 5
		Mass is too spherical and too far away; the perspective should be from very close up, almost in amongst the misty mass
Cars passing	Similar experience to that of rain, whiter in color, again a mass in the middle, as car passes getting bigger and expanding, then getting smaller, yellow strip at top. White ball most dominant	3 of 5
		Yellow strip at the top is accurate. For the mass, the color is correct and the effect of some parts being darker and some parts lighter is good, but again the shape is too spherical and too far away. The mass should look more like a cloud or a chunk of mist
White noise	This elicited a blade in the center moving closer. It was yellowish-white in color and metallic. If he turned around and saw it side on it would be like TV interference, but he's seeing it from the other angle.	4 of 5
		Should be longer on the y-axis, like a sheet of paper on its edge. However, static texture is good and the way it comes to a point is good

### Discussion

This study explored the concurrent visual experiences of six synaesthetes. Our aims were to provide recreations of grapheme-color and auditory-visual synaesthesia using both a traditional color picker and a VR package and to determine whether VR is capable of capturing elements of our participants’ synaesthesia that traditional methods cannot.

For participants with grapheme-color synaesthesia only, results indicated that color pickers are relatively good at capturing their synaesthetic experiences. Most participants consistently ranked their depictions as 4 or above on a scale of 0–5, indicating very good to perfect accuracy (*M* = 4.53, *SD* = 0.50)*.* Generally, synaesthetic concurrents were limited to color, but occasionally, participants described accompanying textures or movement. For example, Participant 4 described “x” and “z” as having a metallic texture, and Participant 2 described “June” as a sunset shining through clouds and “7” as shadowy, smoky, and iridescent. Participant 2's associations for “7” and “June” were recreated in OpenBrush (2016). These recreations were described by the participant as the “most accurate” depictions of his experience he had seen and scored more highly than his ratings of numbers and months using a color picker. It appears that when grapheme-color synaesthesia is more complex than simply colors, OpenBrush (2016) is an advantageous method of portraying these additional qualities. However, when color is the only concurrent, color-pickers perform well, and most participants were relatively satisfied with their depictions. Indeed, for most of Participant 2's grapheme-color associations, he did not believe that OpenBrush (2016) was any more accurate than using a color picker.

For participants with auditory-visual synaesthesia, OpenBrush (2016) showed considerable promise as a method of portraying complex elicited experiences. This was dependent on the nature of the concurrent experiences: for example, Participant 5 experienced two-dimensional colored shapes in response to a range of sounds. He expressed that these could easily be captured by Adobe Photoshop, similar to the methods employed by Chiou et al. (2013), and these were therefore excluded from implementation in VR. However, for more complex experiences involving textures, small degrees of movement, and three-dimensions, such as those described by Participants 3 and 4, OpenBrush (2016) was capable of capturing a more complete representation of synaesthetic concurrents than color pickers or static 2D media such as Adobe Photoshop. This can be seen in complex recreations such as the pink waterfall interspersed with purple shards which Participant 3 experienced in response to the sound of cars passing, the twinkling speckles experienced by Participant 4 in response to birdsong, and the aura Participant 3 experienced in response to the phone ringing, all of which were captured with very good to perfect accuracy. Participant 4 commented on how the three-dimensional nature of VR more accurately reflects his synaesthesia than other methods, and that he “likes that you can move” in the VR environment. This was important as the sound of white noise, for example, elicited the image of a blade moving towards him; however, he knew that if he viewed the blade from the side it would have the appearance of television interference. Using OpenBrush (2016) for this purpose has allowed for a richer and more accurate depiction of multifaceted concurrent experiences than has previously been achieved.

Regarding auditory-visual synaesthesia, general trends have pointed to higher-pitched sounds being lighter, brighter, and located higher in space than low-pitched sounds (Ward et al., 2006). Three of our participants reported auditory-visual synaesthesia and were played a selection of everyday sounds, musical notes, and voice stimuli. As the focus of this study was recreating visual aspects of synaesthesia, the fundamental frequencies of each sound were not taken into account. However, a selection of male and female voices was played, and male voices tend to be lower than female voices (Rendall et al., 2005), although variations exist depending on the individual. Additionally, musical notes were played on a scale. With voice stimuli, Participants 4 and 5 both explicitly stated that higher-pitched voices were seen higher in space, and female voices tended to be experienced as higher up than male voices. No differences were observed regarding the color of voices depending on pitch; however, this was not formally analyzed as it was beyond the scope of this study (see Moos et al., 2013 for a detailed analysis).

Regarding musical notes, Participant 4 experienced three-dimensional shapes in response to notes which were specific to the instrument being played. These shapes moved up or down in space depending on pitch. Participant 5 played short melodies rather than notes, as he expressed that his elicited experiences were related to timbres and tones rather than individual notes. Still, for most melodies, his concurrent visual experiences were a horizontal line of triangles, with the position of individual triangles moving up and down depending on the pitch of notes played within the melody. This was similar to how Participant 4 experienced melodies, except he experienced one three-dimensional shape which moved around in space depending on the pitch of each note. For Participant 3, no clear trend was observed relating to the pitch of notes she was played. Regarding synaesthetic color, Participant 5 stated that “bass sounds trigger dark colors” while high-pitched sounds, such as a “bright, high violin,” would be white. This trend was not observed in Participants 3 and 4; rather, each instrument tended to elicit its own unique color regardless of the pitch of the note it was playing.

Although some common auditory-visual trends were observed, the overall impression from these data is the variety of concurrent experiences and the differences in content between individuals. For example, all three participants were played the same sound clip of cars passing each other on a road, and this elicited the following three experiences: a pink waterfall with shards or bubbles (depending on volume), a white mass moving underneath a yellow strip of color, and a slightly uneven, dull gray band. Although the results are interesting and in line with research showing commonalities between pitch, brightness of color, and location in space (Ward et al., 2006), it is the differences in experience that truly stand out, and which this study best captures.

#### Limitations and Future Directions

As previously mentioned, due to the COVID-19 pandemic, our initial method which involved participants operating the VR equipment themselves was revised in favor of the “VR-by-proxy” method described in this report. Some drawbacks to this method were seen, with participants expressing difficulty in explaining their experiences accurately. Previous research has highlighted how challenging some synaesthetes find verbalizing their experiences; comparing it, for example, to explaining the color red to someone who cannot see (Sollberger, 2011). Additionally, in instances where spatial location is involved, precisely locating the synaesthetic concurrent in space has been identified as particularly challenging to articulate (Chiou et al., 2013). Therefore, it is likely that the original method where participants used the equipment themselves would have resulted in more accurate recreations, as much of the feedback was related to the researcher's drawing rather than the tools which were chosen. Given this limitation, a natural progression is to move on from the “VR-by-proxy” method and allows participants to use the equipment themselves. Thankfully, COVID-19 restrictions eased enough for us to do this.

## Experiment 2

In this experiment, three audiovisual synaesthetes used the VR equipment themselves to recreate their visual experiences in response to five sounds. These sounds were played as the participants were in the VR environment for a fully immersive experience. The five sounds chosen were the “everyday sounds” from Experiment 1. This was because in Experiment 1, the everyday sounds section appeared to be appropriate for all audiovisual synaesthetes, while responses to musical and voice stimuli were more varied: in some cases, one word was not enough to elicit a concurrent response, while in other cases, a short phrase would produce a visual concurrent too complex to describe or recreate as it would require moving animations. Similarly, in some cases, one note would be too simple to warrant recreation in VR, while melodies would be too complex. We chose to focus on audiovisual synaesthesia only because Experiment 1 indicated that for grapheme-color synaesthesia, VR only very occasionally provided benefits compared to traditional methods such as color pickers.

### Method

#### Participants

Three synaesthetes were recruited for the study (two females and one nonbinary). Mean age was 25.3. Full demographics information for each participant is presented in Table 6. Participants were recruited based on their self-reported audiovisual synaesthesia. Two of the three participants were later retested as a test of genuineness.

**Table 6.** Participants’ Demographic Information for Experiment 2.

Participant	Gender	Age
1	Nonbinary	21
2	F	31
3	F	24

#### Apparatus

Participants were tested using an Nvidia GTX 1080 I Gaming PC (Windows 10) with HTC Vive Pro VR kit (Vive, 2016). The experiment was run through OpenBrush (2016) (Google, 2016) using SteamVR software (Steam, 2003). HTC Vive Pro resolution is 1440 × 1600 pixels per eye (2880 × 1600 pixels combined). Refresh rate was 90 Hz. Audio was supported by Hi-Res certificate headset Hi-Res certificate headphones (removable) with high impedance headphones support.

#### Stimuli

Everyday sounds used in Experiment 1 were selected: cars passing, birds chirping, white noise, phone ringing, and rain (see online supplementary materials for audio recordings). In order to adjust for the volume and allow participants to get used to the audio set up, simple acoustic music was selected for the onboarding (royalty free)—“Beat of Nature.mp3” (audio is available at online supplementary materials).

#### Procedure

The procedure was significantly simplified from Experiment 1. In this instance, we were interested in audiovisual synaesthesia and wanted to allow participants to draw their perceptual experiences in VR themselves. Also, the primary focus was qualitative analysis, as in Experiment 1, the nuances of participants’ reports were missed without any formal qualitative procedure.

The current experiment was approved by the College of Science and Engineering, University of Glasgow Ethics committee. Participants read the information sheet and signed a consent form before arrival at the VR lab. Upon arrival, the experiment was explained to the participants, and the VR kit was demonstrated. Once participants were comfortable they were placed in the virtual environment. Open Brush was used for onboarding^3^. Participants were introduced to variable features of the tool, including environments they could draw in, adjustment of the size of the brush, and the brushes themselves. Some standard prompts were provided for participants to get comfortable in the environment and the teleporting tool was introduced to demonstrate how they can move easily in three-dimensional space. In the meantime, the experimenter was monitoring the physical space to ensure participants did not walk into objects in the VR lab.

Once participants were comfortable using Open Brush, an onboarding sound was introduced. The volume was adjusted to the participant's comfort and they attempted to draw the perceptual experiences the music elicited. At this point, a recording of the video screen (virtual environment from the first-person perspective) and participants’ comments were started. Participants were encouraged to describe their experiences and thought processes as they went along.

When participants were comfortable with the virtual set-up, experimental sounds were played. Sounds were played in the same order, however, due to technical difficulties, the “White Noise” sound was played as the second stimulus for Participant 2, but the remaining order continued after. See Table 7 below for the full order of stimuli presented to all of the participants.

**Table 7.** Order of Stimuli Presented to Each Participant During the Experiment.

Participant	Stimulus 1	Stimulus 2	Stimulus 3	Stimulus 4	Stimulus 5
1	Bird song	Cars passing	Phone ringing	Rain	White Noise
2	Bird song	White Noise	Cars passing	Phone ringing	Rain
3	Bird song	Cars passing	Phone ringing	Rain	White Noise

Once again participants were encouraged to describe their experiences as much as possible. Each sound lasted approximately 30 s, therefore, the sound was replayed as many times as participants requested. After each stimulus, participants were asked to rank how accurately their drawing represented their perceptual experiences, from 0 to 5, where 0 is not representative at all and 5 is perfect.

After all five stimuli were presented participants were asked general debriefing questions on their impressions of the VR and how well they thought the tool helped them represent their perceptual experiences.

#### Retest

In order to get some idea of the consistency of responses, Participants 1 and 2 were retested on the same task approximately 19 weeks after their first session.

### Results

#### Ratings

Participants rated most of their drawings fairly highly as representative of the actual perceptual experiences. Due to the issues with VR controllers, the final rating for Participant 3 is not available. Participant 3 described their experience in this instance but was unable to produce a drawing. Thus, only qualitative data were collected in this instance. Full details of the ratings, their means and standard deviations are presented in Table 8.

**Table 8.** Ratings of How Representative Each Drawing was of True Perceptual Experiences, Where 0 is not Accurate at all and 5 is Very Accurate. Means and Standard Deviations Represented for Each Stimulus.

Participant	Bird Song	Cars Passing	Phone Ringing	Rain	White Noise
1	4	4	5	3	3
2	5	5	3	3	4
3	4	5	5	2	NA
Mean	4.33	4.67	4.33	2.67	3.50
SD	0.58	0.58	1.15	0.58	0.71

#### OpenBrush Drawings

**Figure 7.**
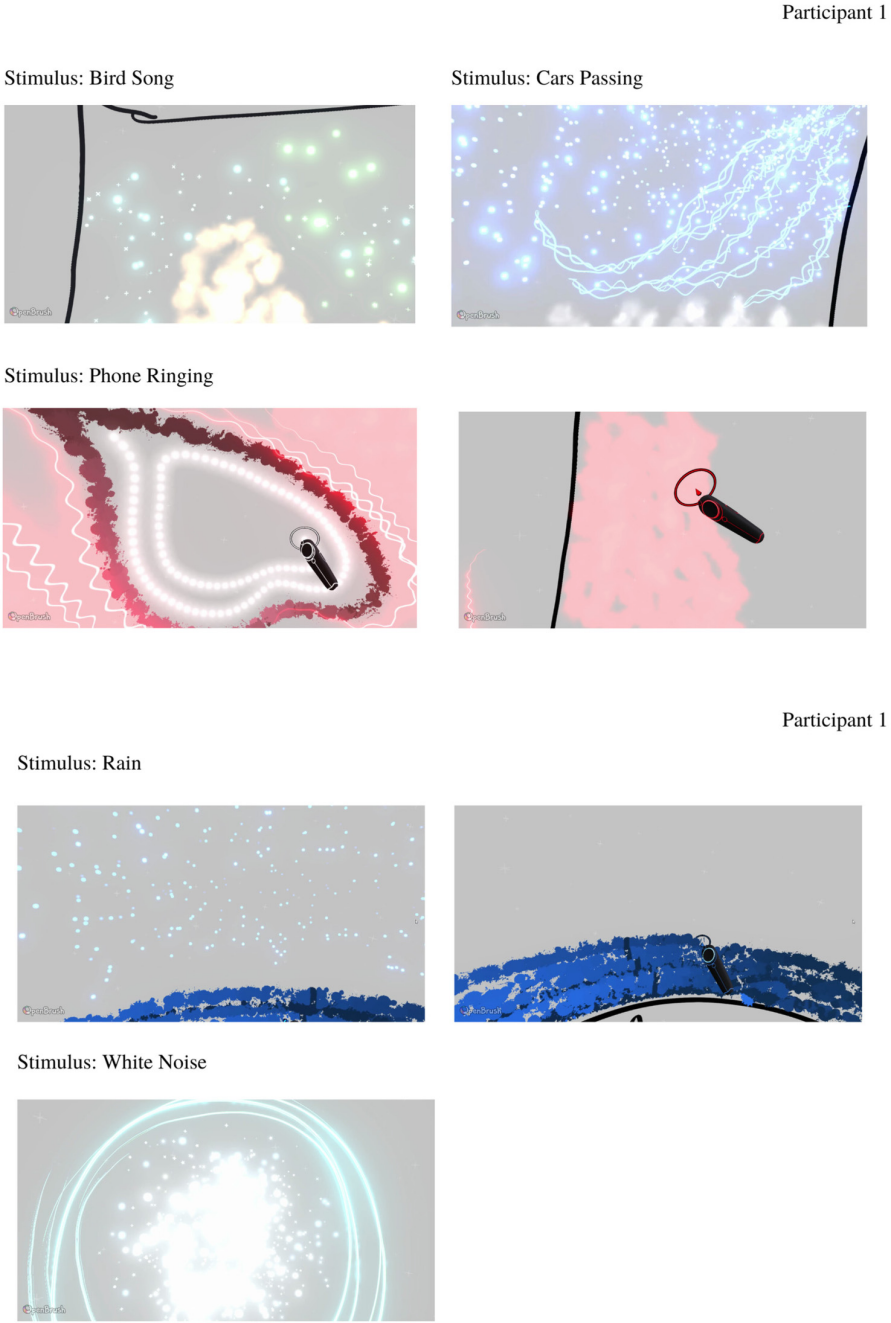
Participant 1 drawings in response to experimental stimuli.

Individual drawings made by the participants will be briefly discussed in this section before qualitative thematic analysis is explored. Participant 1 chose to produce drawings in the default gray environment. Bird song elicited clear colors and textures: yellow and sparkly. Cars passing had a bluer tone and animated, directional movement. Phone ringing, Rain, and White Noise stimuli were also represented mostly using animated brushes and colors which were elicited by the sound almost immediately (Figure 7). Full videos of the recreations are available in online supplementary materials.

**Figure 8.**
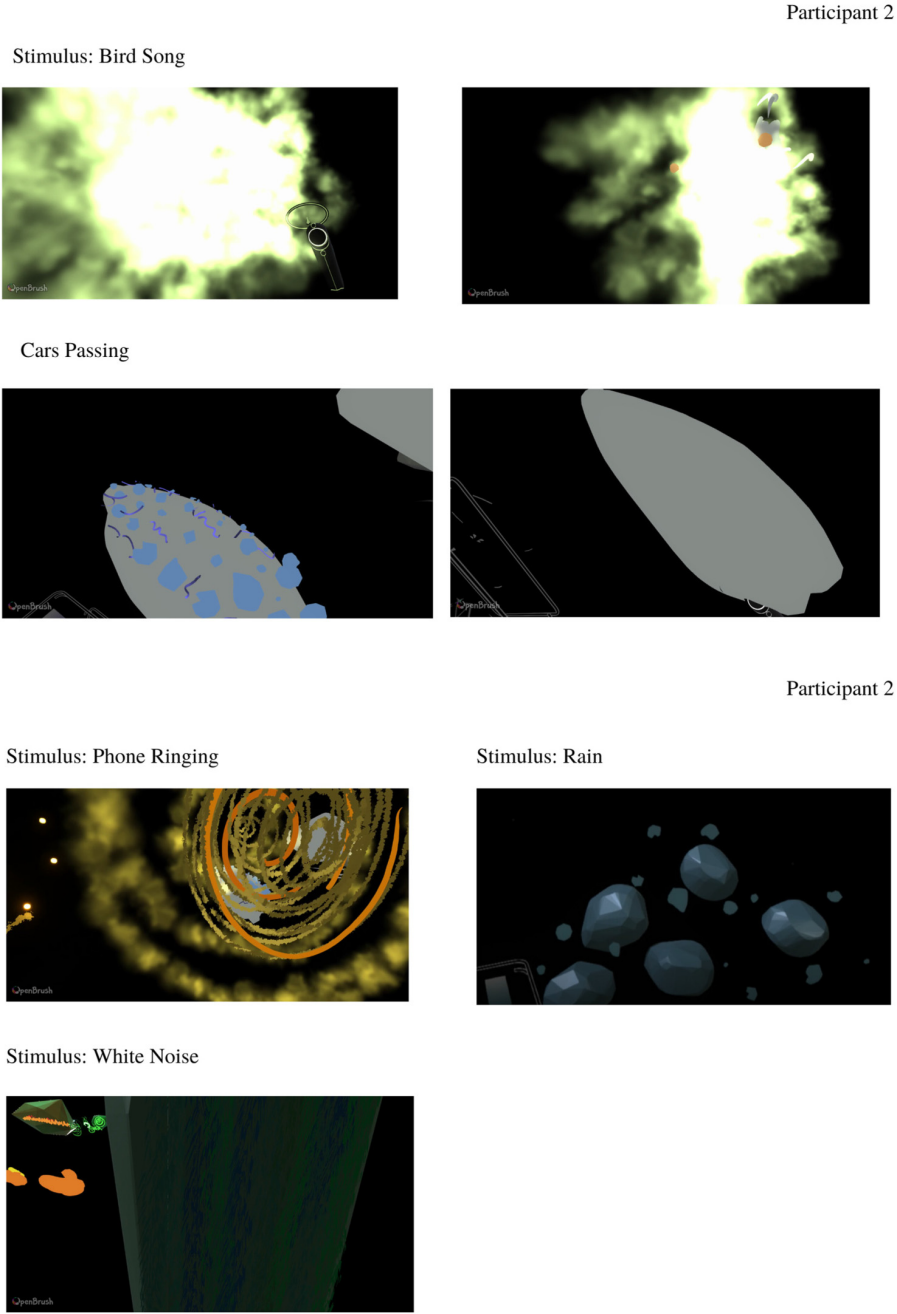
Participant 2 drawings in response to experimental stimuli.

Participant 2 produced drawings in a dark environment. The majority of the drawings had defined shape and clear colors. Bird song elicited strong color and movement. The white noise drawing was produced using smoky and speckle brushes. Cars Passing and Rain sounds elicited a specific shape with movement in some parts of the drawings. The Phone Ringing drawing was made using several animated brushes (Figure 8). See online supplementary materials for videos of each recreation.

**Figure 9.**
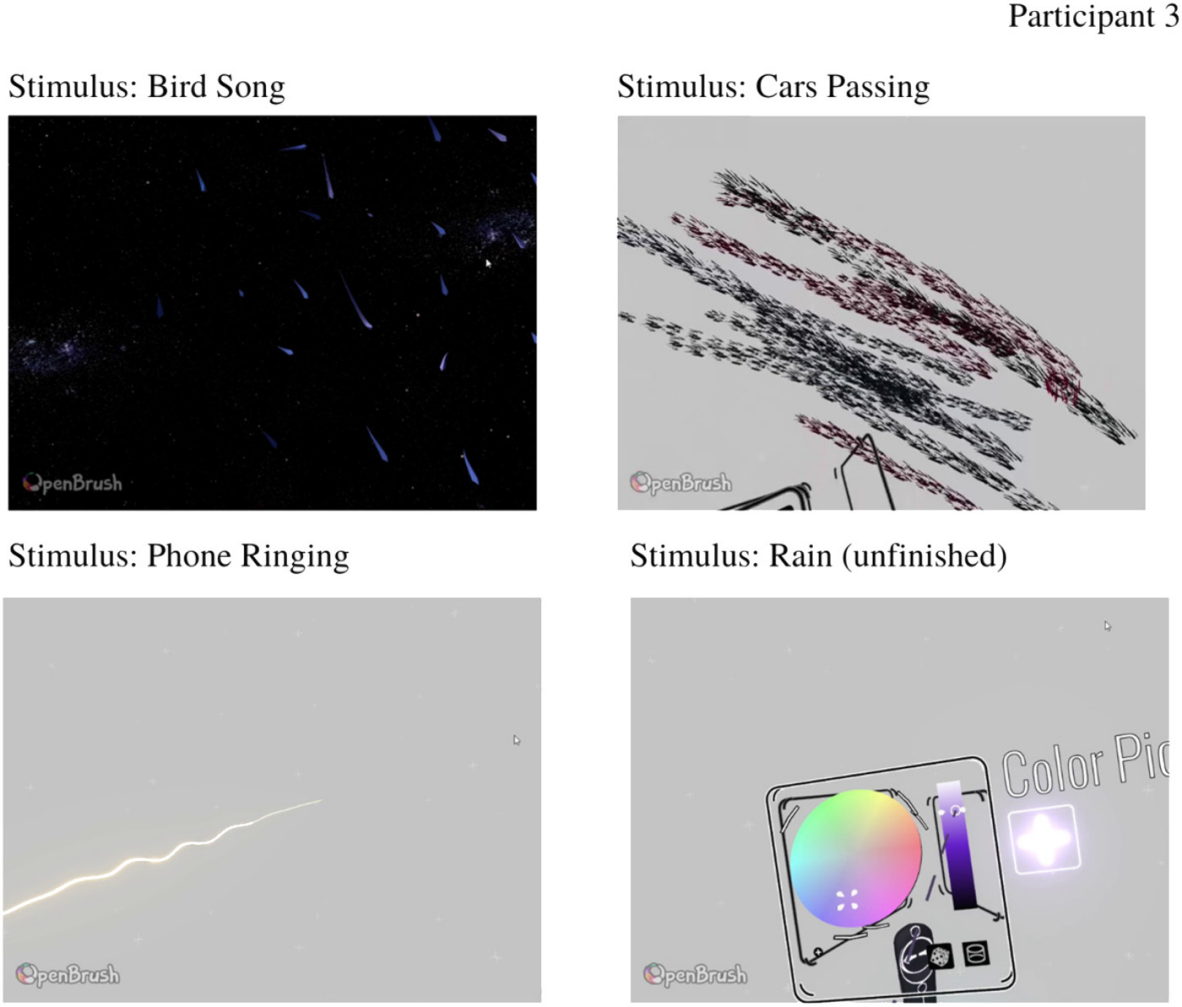
Participant 3 drawings in response to experimental stimuli.

Finally, Participant 3 initially chose to work in the dark environment for the first stimulus—Bird song, then changed environment color to default gray as it was hard to visualize certain colors and movements that they wished to use. Cars Passing and Phone Ringing stimuli elicited strong movement and texture with specific placements in space (Figure 9). Due to technical difficulties, the final stimulus of Rain was unfinished, and the participant just selected the color (light purple) and White Noise was not drawn at all. However, the participant did describe what type of sensations and perceptual experiences were elicited by the stimuli. Full analysis, transcripts, and videos of the participants’ drawings are available in online supplementary materials.

#### Qualitative

Audio recordings were transcribed verbatim and recordings themselves were deleted after transcription to comply with the Ethical procedures and GDPR guidelines. Thematic analysis was chosen as an analysis method for the qualitative data as this approach allows research on individual views, opinions, and experiences. ITA was used to analyze the transcripts (Braun & Clarke, 2006), which means that the data determined the themes presented. Semantic (explicit content of the data) and latent (subtext underlying the data) aspects were both used in the analysis. As we are dealing with the descriptions of perceptual experiences what was said was equally as important as how it was said.

Six steps developed by Braun & Clarke (2006) were followed to analyze the data: familiarization, coding, theme generation, review of the themes, definition, naming, and write up. Two researchers coded and generated themes independently and then reviewed overlapping themes and decided on the final names and subthemes. Nvivo 12 (2020) was used to analyze and code the transcripts. The following themes and subthemes were extracted (Table 9).

**Table 9.** Table Represents the Main Themes and Subthemes Identified by the Qualitative Analysis.

Theme	Subthemes
Features of drawings and perceptual experiences	Basic Features
	Movement & Texture
The use of Virtual Reality	Accuracy of representation of perceptual experiences
	Difficulty to describe experiences and drawings
	Eliciting memories and sharing experiences
	Engaging with tools and features
	Enjoyment of using Virtual Reality
Diversity of synaesthetic experiences	Cross modal
	Dimensionality

### Theme 1: Features of Drawings and Perceptual Experiences

Participants spoke in detailed terms about the specific features of their drawings. These have been broken down into more basic elements of the drawings, namely color, shape, and size, and more complex features, consisting of movement and texture.

#### Subtheme—Basic Features

Basic features such as color, size, and shape were referenced often by participants and appeared to be captured well by VR. Color was an important feature in most recreations and in some cases, participants described how different elements of the drawing changed color.

Participant 1: … there are different shades of blue because these, like electric bits. Here are a little bit lighter it's very yellow and orange, so I've done like a big yellow shape and then like a sort of orange line in the middle. But there's like other things coming through as well so. Like the little Guitar scratches are kind of silver, so…

Shape was also important in the majority of the recreations. Participant 2 expressed most of their experiences via a shape first and then discussed influences of color and size:

Participant 2: … Is very big to begin with and then it's sort of like get a little bit less… And shape that I get from it and then the little brown speckles are kind of almost like they are birds there's usually some kind of like sort of sideways eye shape in the middle…

Overall, the Basic Features subtheme captured the very first descriptions of the experiences, as it often started with either specific color or shape. Then participants explained it in more detail and more nuanced experiences were explored. This is captured by the second subtheme—Movement + Texture.

#### Subtheme—Movement + Texture

Movement was a key element to many drawings for all participants. In some instances, this could partly be captured by VR. For example, Participant 1 chose one of the animated brushes to represent their experience in response to the sound of a phone ringing. The “*circular motion”* that this brush provided was *“quite important”* in conveying their experience; however, the speed of the motion should have varied depending on its position within the drawing.

Participant 1: …in the middle it's slower, but on the outside it moves a lot more, almost like a sort of gravitational vacuum.

However, for other visual concurrents, the associated movements were not something that OpenBrush (2016) in its current form could accommodate.

Participant 1: So basically from the center here everything is pulsating outwards as if it's churning round, yeah. But I can't obviously, draw that, so that would be sort of the reverb and it's not gonna work.

**Figure 10.**
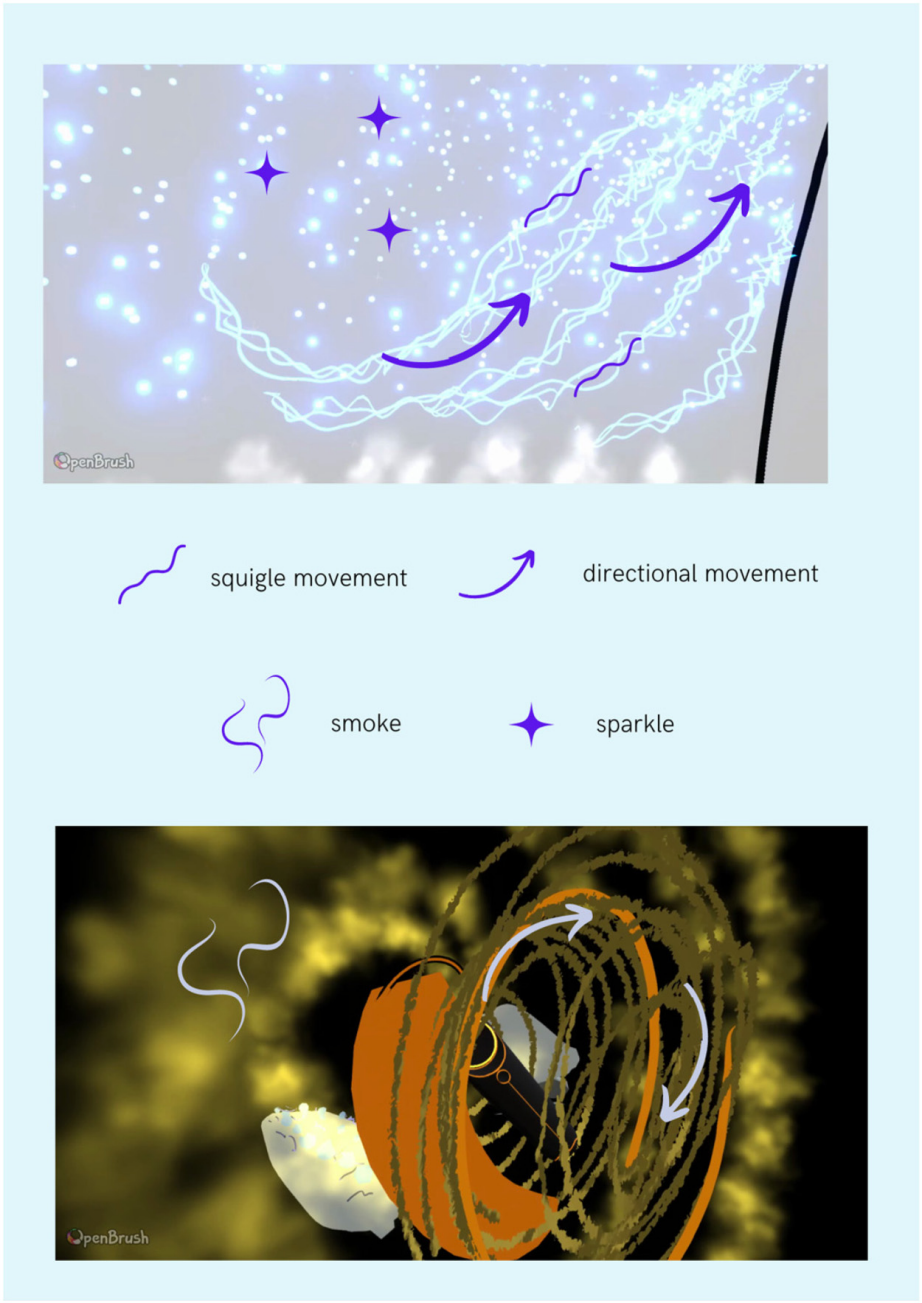
Top image is a drawing produced by Participant 1 in response to the cars passing stimulus and the bottom image represents drawing produced by Participant 2 in response to the phone ringing stimulus. Several symbols were selected to represent the movement captured in the Virtual Reality (VR) environment: squiggle movement referring to serrated/wave-like movements; directional movement referring to movement in one specific direction; smoke referring to smoke-like animated brush, which was often used by the participants; and sparkle—animated sparkle brush which was fading in and out. Videos of the drawings can be found in online supplementary materials.

Where movement could not be accurately expressed, participants found novel ways to suggest movement, such as collapsing varying-sized rings into one *“mean ring” (Participant 2)* or adding lines to depict swaying motions. Figure 10 represents some of the movement produced by the animated brushes and some described by the participants.

Participant 2: Like floating away from the shape. And then obviously it starts strong and it gets quieter since doing that sort of like shape where it's like really big to really small and then this orange line is like the mean ring.

Participant 1: and these move really slowly and with me waving them. That's just to show that they kind of move in a very solid way

Texture was also important, and the textured brushes, particularly the smoke brush, allowed for this to be well captured.

Participant 1: Yeah, I mean this smoke will actually come in really helpful because a lot of my synaesthesia looks kind of like that.

Participant 1: The embers would still be going over the top as well. You can see the embers through it. Yeah, and the bottom bit with smoke. Yeah, it's just a little bit fuzzy.

Participant 2 also referred to the “*smokiness*” and “*cloudiness*” of her sensations, as well as describing “*stony, rough*” textures, while Participant 3 described the texture of one object as if it had been cut by a “*serrated knife*.”

The Movement + Texture subtheme was of utmost importance, although somewhat limited by the brushes currently available. Each drawing produced by the participants had some type of movement, either accurately represented by the animated brushes or described by the participants themselves.

### Theme 2: The Use of VR

#### Subtheme—Accuracy of Representation of Perceptual Experiences

Participants discussed how drawing their perceptual experiences in VR allowed for more accurate depictions of their synaesthesia than they had previously achieved in other media, for example, pen and paper.

Participant 1: Yeah, I mean this is the best I've ever gotten to be honest. This is the best I've ever seen that can describe the sort of stars in your eyes. [in reference to the stars effect brush] …I never got to represent my experiences so clearly. I would like to show it to my family.

Participant 2: There's so much more that I can convey and describe about it because I'm able to use like a 3D space rather than trying to draw something on a piece of paper. So yeah, a massive advantage.

Participant 3: You need to just draw what I can see. Pen and paper are too small and doesn't get the same accurate representation so…

The introduction of animated brushes, a three-dimensional environment, and additional ways to represent movement and texture of their experiences allowed participants to capture and describe their perceptual experiences better.

#### Subtheme—Difficulty to Describe Experiences and Drawings

Participants spoke about how describing their experiences with words was difficult and the specific aspects of this difficulty were also hard to articulate.

Participant 1: It's really quite difficult to describe that…

Participant 2: It's hard, it's hard to describe so… It sounds really difficult… Yeah, it's it's really hard to describe.

Participant 3: There's not as much…. I don't know how to sum up this picture

In some cases, this difficulty extended into drawing as well. In some instances, this might have been due to limited drawing skills and artistic abilities.

Participant 3: Yeah, definitely. It's mostly difficult to sometimes describe it, if I can't draw it.

In other cases, it could have been due to the experiences themselves. Although, once again participants did not have an easy time articulating what specifically was difficult.

Participant 1: ‘cause sometimes it's not so easy to draw what I see. Sometimes it will sort of feel a little bit like this and I don't really get much else.

Participant 2: I think it is quite difficult to get this one. The general like hard shape. Is like definitely what I was thinking of..

There can be several reasons for this difficulty. The most obvious ones are the artistic ability of the participant and the way the experiences are described alongside the drawings. However, in some cases, participants might not have had enough time to fully think about how they wished to represent their experiences, and the novelty of the tool added an additional layer of complexity.

#### Subtheme—Eliciting Memories and Sharing Experiences

Being in the VR environment elicited a wide range of memories and past experiences that the participants discussed. Creative activities can often elicit these types of experiences and willingness to share and can be traced back to basic principles of art therapy, although this was not an intention of the current experiment (Hacmun, Regev & Salomon, 2018).

Participant 1 described their love for music and how they can “*play several instruments,”* and how different instruments elicit different experiences. The texture was also a dominant feature in their accounts.

Participant 1: .. for example, I can sort of see a lot of the texture of the strings. I can see when they're getting plucked. And it's stimulating, but if I was hearing a much more grainy and overdriven guitar..

Participant 1 also expressed they were “*a big metal fan*” and that the taste in music is linked to their synaesthetic experiences. They also shared that they are “*trained in [culinary] knife skills*” and the sounds and smells associated with the kitchen elicit strong memories and perceptual experiences. They also shared their love for cycling and “*the feeling of … chain on the bike between . .. pedals”* which made them think of “*..like gray sort of rocks.. like sort of grinding against each.”*

Participant 2 also shared that their tastes in music were linked to their synaesthetic experiences.

Participant 2: like I do listen to quite a lot of rock music. So it depends on like the type of song.

They also described how they perceive time and numbers and that they are represented spatially, as if on a map.

Participant 2: I am on this map. Where I am at 31. And everybody else I know is like placed across the map.

Participant 3 discussed how the sound of rain reminded them of everyday experiences living in Scotland and how colors and sounds clearly had a very strong association with memory. They also expressed how their music preferences are linked to their perceptual experiences.

Participant 3: I don't like you know like skating music because I feel like it's really harsh.

Whenever participants were placed in the virtual environment they were happy to discuss their immediate responses to the stimuli, but that also often connected to specific memories or daily experiences. Due to the qualitative nature of the study, these experiences can be captured and analyzed.

#### Subtheme—Engaging with Tools and Features

OpenBrush (2016) provides a variety of brushes and many of them are animated or unusual, often three-dimensional textures, which helped our participants express their perceptual experiences better. The smoke brush was certainly one of the most popular tools used.

Participant 1: Yeah, I mean this smoke will actually come in really helpful because a lot of my synaesthesia looks kind of like that…

Participant 1 also remarked on the animated sparkle brushes as very true to actual perceptual experiences.

Participant 1: Oh my God… Yeah, this is. Pretty much. This is the best I've ever seen that can describe the sort of stars in your eyes.

The ability to make three-dimensional shapes was admired by Participant 2 and Participant 3 found many brushes hyper-realistic.

Participant 3: I feel like I could just touch them. They're very realistic.

All participants engaged with various brushes and liked to experiment with texture. Alongside verbal descriptions of the process and the perceptual experiences themselves, the data collected had a rich qualitative account of synaesthetic perception.

#### Subtheme—Enjoyment of Using VR

The novelty of the tool and the ability to engage with the three-dimensional world with a variety of tools was enjoyable for all of the participants. They often commented on how “*cool” (Participant 2)* and “*realistic” (Participant 3)* it felt.

Participant 1: I don't think it matters how long I would spend in here. It would never stop like amazing me.

Enjoyment of the new tool has certainly been one of the key subthemes in the experiment, however, it did not deter from the task at hand. Participants completed their drawings without getting distracted by other available tools with guidance from the experimenter. However, the enjoyment should be explored further in any follow-up work.

### Theme 3: Diversity of Synaesthetic Experiences

#### Subtheme—Cross Modality

All participants mentioned that the auditory stimuli elicited further cross-modal experiences, in addition to visual experiences. For Participant 3, these were primarily gustatory experiences:

Participant 3: This is going to sound strange. But I feel like I can taste this. So it's definitely a color. And yeah… but it tastes like, you know, you get those… Little tubs of pears”

Participant 1 said that they could “*see, smell, touch and [hear]”* the stimuli, similar to how one might experience a memory. Participant 2, meanwhile, referenced physical sensations, such as feeling like her feet might get wet and experiencing the sound lower in her body as a result: “*it feels very low down… thinking my feet are going to get wet.”*

As a result, some of the visual perceptual experiences were fairly weak for some participants, which also depended on the stimuli. Participant 3 especially reported a lot of cross-modal experiences and some of the visual experiences were either weak or very hard to present as a three-dimensional drawing.

#### Subtheme—Dimensionality

The relevance of where the elicited visual experiences were in relation to participants’ bodies was mentioned by all participants and is something that VR is uniquely capable of capturing. Participant 1 talked about how he saw something at the periphery of his vision:

Participant 1: I'm just in the corner of my eye. It's just a little bit of this here, and it's about that color.

Participant 2 referred to “*standing”* on, “*walking through”* and “*being inside”* various objects, in addition to characterizing other items as “*floating in space.”* Similarly, Participant 3 mentioned being at first beside and then behind one construction:

Participant 3: Seem to be at the side of both of my ears. Not only that… I wouldn't put it front of me. I would envision it to be beside…and then we're behind it.

VR is a unique tool for representing the three dimensionality and movement of the perceptual experiences described by the participants. Although some aspects of the experiences were difficult to capture, the dimensionality and position in space subtheme reveals that there are three-dimensional aspects to synaesthetic experiences that should be explored further.

### Retest

**Figure 11.**
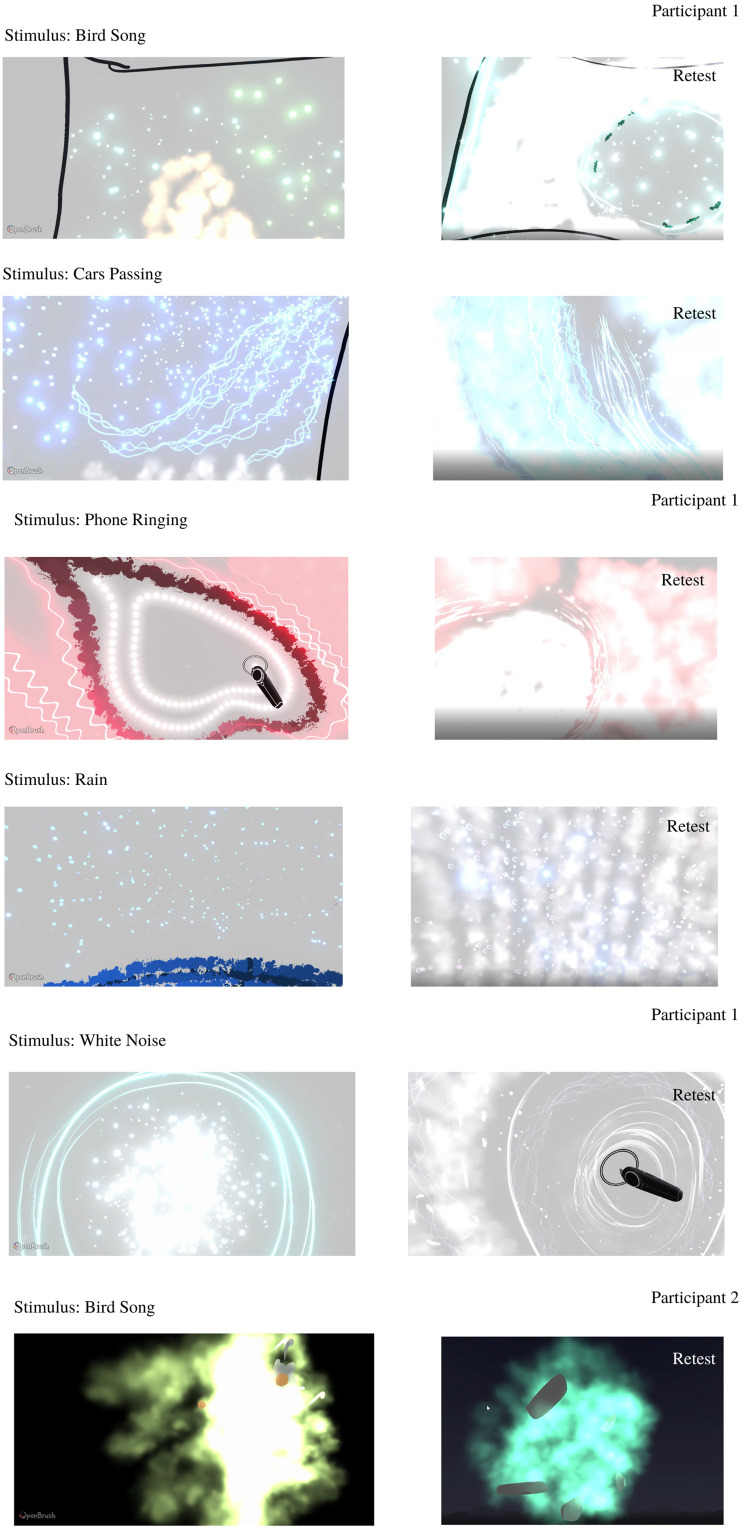

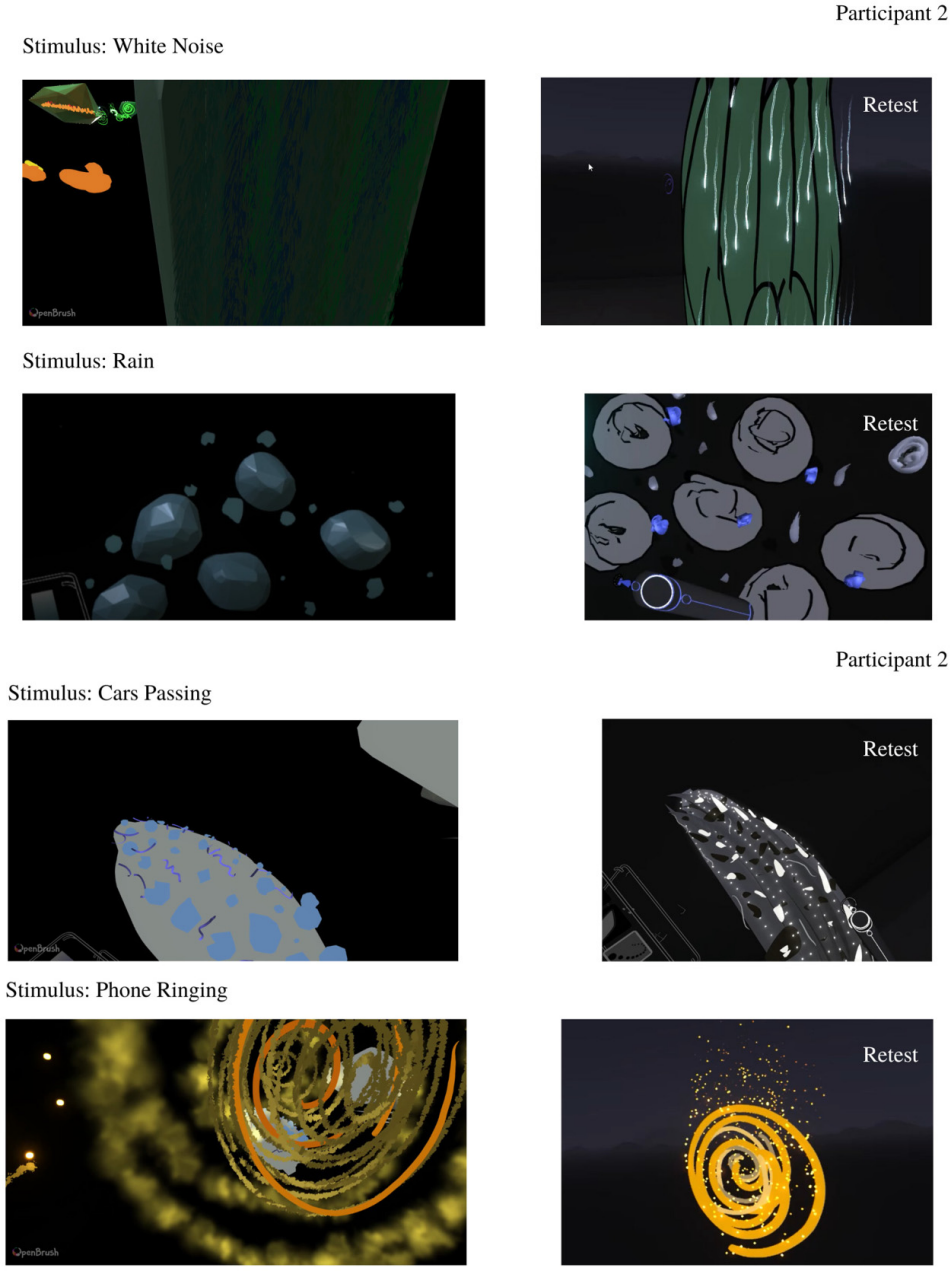
This figure represents video captures of their drawings for all stimuli for both the original experiment and the retest.

Participants produced similar drawings in the retest. Although there were some differences seen in the brushes used or the intensity of colors chosen there are clear similarities in terms of the overall shapes, hues, and gist of the retest drawings which is striking given the number of degrees of freedom available (see Figure 11).

### Discussion

Experiment 2 explored the perceptual experiences of three audiovisual synaesthetes. In contrast to Experiment 1, in Experiment 2 participants used the VR equipment themselves to recreate their additional perceptual experiences in response to five everyday sounds. We aimed to examine whether the VR environment can allow synaesthetes to create richer and more accurate depictions of audiovisual synaesthesia than two-dimensional media.

Participants rated most of their drawings relatively highly on a continuous scale of 0–5 (*M* = 3.93, *SD* = 0.997) (see Table 8). These ratings were assigned by the participants in order to provide an understanding of how accurately their drawings matched their synaesthetic experiences and were not formally analyzed. A range of reasons was given for why some drawings were rated as less accurate than others: participants had difficulty finding the best brush, had relatively weak synaesthetic associations for certain sounds, or may have required additional time to perfect the image.

Qualitative analysis revealed the following themes: Features of drawings and perceptual experiences, The use of VR, and Diversity of synaesthetic experiences (Table 9). Features of drawings and perceptual experiences were broken down into two subthemes: basic features and movement and texture. Basic features were color, shape, and size generally could be captured well in OpenBrush (2016); however, similar results could likely be achieved with more traditional media. Movement and texture were both important elements of synaesthetes’ concurrent experiences. Textural qualities, most notably smokiness and cloudiness, were referenced often by the participants and inbuilt brushes allowed for this to be well captured. In keeping with Experiment 1, small degrees of movement, such as the circular motion experienced by Participant 1 in response to the sound of a phone ringing, could be accommodated by OpenBrush (2016). More complex movements, however, such as objects changing size over time—a phenomenon mentioned by both Participant 1 and Participant 2—could not be captured so well (see the limitations section for a more detailed discussion).

The diversity of synaesthetic experiences encompassed the dimensionality aspect of participants’ concurrent experiences, as well as any additional cross-modal experiences they reported, such as taste or physical sensations. Although additional cross-modal experiences cannot be captured within OpenBrush (2016), these details provide additional context to the participants’ visual recreations and a broader picture of the degree of multisensory integration within synaesthesia. In terms of position in space, participants clearly indicated where visual concurrents should be in relation to their own bodies in the VR space. Participant 1 described seeing things within his visual field, such as in his peripheral vision, whereas Participants 2 and 3 described being behind, beside, above, or within their synaesthetic experiences. This demonstrates the importance of three-dimensional media in attempting to recreate the experience of synaesthesia, as dimensionality cannot be adequately captured by other artistic media yet appears to be fundamental to participants’ synaesthetic experience.

The use of VR contained the following subthemes: accuracy of representation of perceptual experiences, difficulty to describe experiences and drawings, eliciting memories and sharing experiences, engaging with tools and features, and enjoyment of using VR. Participants highlighted how, on some occasions, the VR medium allowed for the most accurate representation of the experiences that they had experienced so far. This was owing to several factors including the three-dimensional, immersive nature of VR and the availability of textured brushes such as the smoke and star brushes. Participants mentioned how difficult their synaesthesia could be to describe, and in some cases, the VR environment allowed them to express this. However, in other cases, this difficulty extended to drawing. Although VR requires less specialist skills than media such as animation, this can possibly be due to differing levels of artistic skill, as well as the short time participants had to become acquainted with the extensive features of OpenBrush (2016). A benefit of the case study approach and allowing participants time to explore and discuss their additional sensations was that memories and previous experiences were elicited. This allowed participants to share aspects of their synaesthesia that, although not directly relevant to the task, provide a broader picture of how their synaesthesia influences their daily lives. Participants also expressed enjoyment and a desire to spend more time in the VR environment (Wagler & Hanus, 2018; Lee, Kim & Choi, 2019).

An important issue is to what extent synaesthetic experiences vary over time. The idea of consistency as the gold-standard test for synaesthesia has been questioned in recent years, and some studies have demonstrated lability in synaesthetic concurrents with age and mood (see Lacey et al., 2021, for a recent overview). In our case, the differences between test and retest are probably due to the participants’ lack of experience with the brushes and features of OpenBrush (2016). As in session 1, they were still growing accustomed to the OpenBrush (2016) landscape. Additionally, as in session 1, participants occasionally reported difficulty in depicting their experiences accurately, especially when the concurrents included movement. A full exploration of the consistency of the depictions over time, and how this relates to the “genuineness” of synaesthesia, will be a feature of our future work.

## General Discussion

This paper has presented two experiments investigating the use of VR to explore the phenomenology of grapheme-color (experiment 1 only) and audiovisual synaesthesia. Due to constraints presented by the COVID-19 pandemic, in Experiment 1 a VR-by-proxy method was used. In Experiment 2, participants were able to use the VR equipment themselves to create their own representations of their synaesthesia. In both experiments, participants responded very favorably to the VR environment, and in some cases stated that their VR drawings were the most accurate depictions of the synaesthesia they had ever managed to create.

A clear benefit of using VR to recreate synaesthetic associations is the opportunity it affords to create drawings in an immersive, three-dimensional space. In both experiments, participants refer to where their concurrent experiences are positioned relative to their own bodies. For example, in Experiment 1, Participant 4 reported on viewing the researcher's recreation of the misty gray mass elicited by the sound of rain that rather than viewing it from a distance we should be very close up, almost among the mist. Similarly, for the speckles experienced in response to birdsong, two perspectives were shown, one from a distance and one from closer up. Although the far-away version was judged as very accurate, the participant said that he found the close-up version hard to judge as he did not experience it from such a close-up perspective. Participant 3 reported that the depiction of the researcher's voice should fade out into it the periphery, rather than hovering around a definitive edge. In both cases, the importance of positioning was something that participants mentioned only in meeting 2, but that they felt strongly about. This highlights the difficulty that participants had in verbalizing their additional perceptual experiences, a finding consistent with previous research (Sollberger, 2011). Additionally, it demonstrates the importance of dimensionality in synaesthetic experiences, which is something that VR is uniquely capable of capturing.

**Figure 12.**
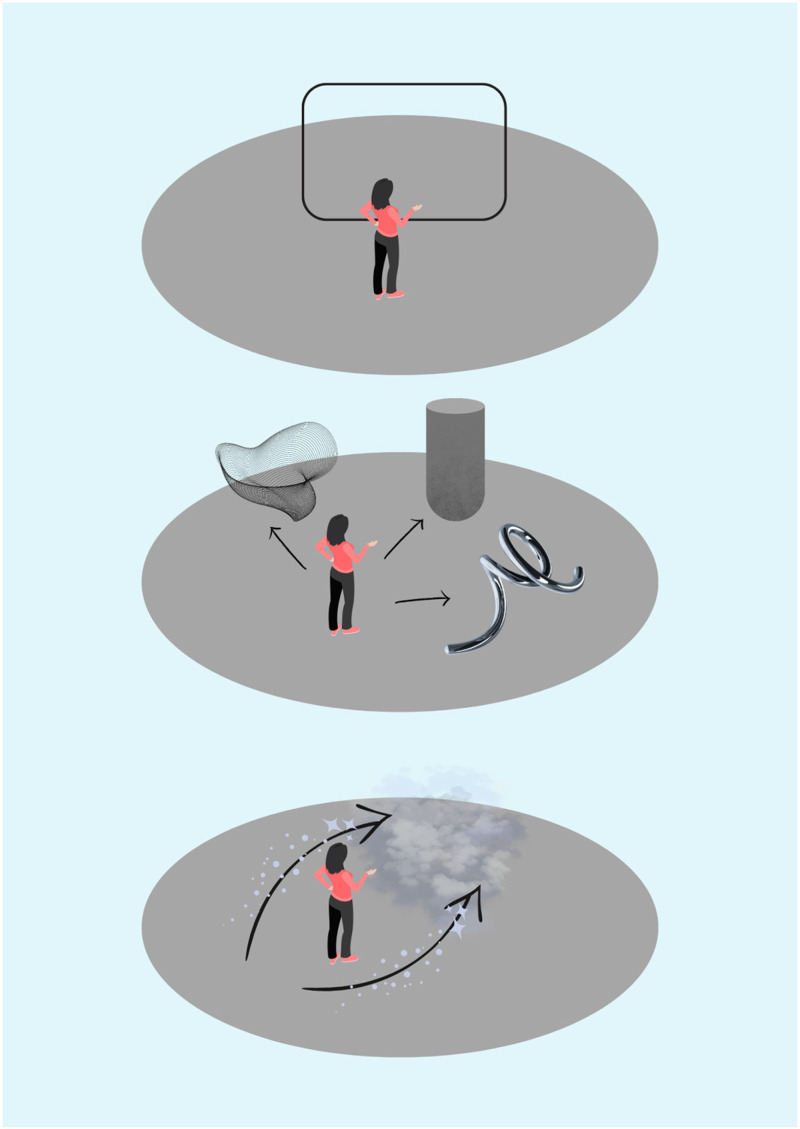
This figure represents spatial orientation of drawings in relation to the participant. Participant 1 (top) viewed things as if on a screen and felt constrained by their visual field, which made them reluctant to explore the three-dimensional space of the virtual world; Participant 2 (middle) drew things relative to their body and discussed moving through and standing on objects in the three-dimensional space; Participant 3 (bottom) again drew objects in relation to their body, but typically beside, behind or in front of them.

These findings were echoed by participants in Experiment 2, who all made repeated references to where objects should be relative to themselves (see Figure 12). For Participant 1, this was generally restricted to two dimensions, as they described seeing their synaesthetic experiences as if on a screen or in their peripheral vision. Meanwhile, Participants 2 and 3 characterized their experiences in three dimensions, as they discussed being “*beside"(Participant 3), “behind” (Participant 3), “inside"(Participant 2) “standing on"(Participant 2),* or “*walking through"(Participant 2)* their various synaesthetic experiences. These differences could reflect whether the participants were associators or projectors and exploring the differences in how associators and projectors choose to represent their perceptual experiences in a virtual environment could be an important avenue for future research.

Participants naturally drew these where they experienced them in space but tended to only describe this when prompted by the researcher. Clearly, where concurrent experiences are with respect to the synaesthetes themselves is an important element of audiovisual synaesthesia, and one which cannot be represented in traditional 2D media. VR allows for this form of expression and allows for nonsynaesthetes to step into the environment and experience a fuller, more immersive picture of another's synaesthesia.

### Limitations and Future Directions

Although showing promise, there were certain aspects of some participants’ elicited experiences that OpenBrush (2016) was not capable of capturing. Primarily, certain types of motion could not be captured. This was seen in both experiments. For example, in Experiment 1, Participant 3 described her reaction to the sound of birdsong as an orange color “jiggling” back and forth in the bottom right corner, which then darted across to halfway up the left-hand side and became a twisting peach octagon. Although the initial subtle “jiggling” motion was well captured, no tool allowed for that shape to then move across the screen, change color and shape, and then twist. Similarly, the sound of white noise elicited the experience of a white wave for Participant 3, similar to that of a heart rate monitor, moving across the screen and fading out as it did so. Again, no tool allowed for this movement to be captured. Furthermore, tools were preset, which reduced accuracy in some cases by subtly altering colors. For example, the brush which was chosen to recreate Participant 3's aforementioned pink waterfall turned white at the end of each brushstroke. Participant 3 remarked that *“the color is not quite right, but the movement is good”* indicating that while this tool could accurately recreate her dynamic concurrent experience, the preset coloring resulted in reduced overall accuracy. Similarly, in Experiment 2, certain types of motion could not be captured, and participants resorted to a range of different measures to allude to motion—such as adding lines to indicate the direction of motion or drawing the “*mean” (Participant 2)* size of rings which should be moving and gradually getting smaller—rather than accurately depicting the motion as they experience it. These examples indicate that while OpenBrush (2016) is an advantageous tool for capturing some visual elements of synaesthesia, it is missing some features which would be necessary to fully capture other elements.

However, a recent development has resulted in OpenBrush (2016) becoming an open-source platform, meaning that while Google will no longer update it, the code is now freely accessible and anyone can make changes to it (BBC, 2021). Although this is beyond the scope of this project, in the future, researchers can modify the program based on their needs, which may address the shortcomings addressed above. Alternatively, accurately capturing motion would require animation programs. In addition to the work of Ward et al. (2008), Michael Gagne and Jeremy Blake created synaesthetic animations on the films *Sensology* (2010) and *Punch-Drunk Love* (2002) where they animated auditory-visual experiences in response to music (Taberham, 2013). However, a great benefit of OpenBrush (2016) is its accessibility, in terms of the time and skill levels required to become accustomed to the technology. Therefore, the open-sourcing of its code may allow for more complex movements to be incorporated into future works, without the need for more complex animation software.

In Experiment 1, a clear limitation was that participants were not drawing the concurrent stimuli themselves. This was rectified in Experiment 2. However, a strength of Experiment 1 was the full and detailed descriptions that participants provided of their experiences, and the specific feedback on how to improve the researcher's OpenBrush (2016) drawings. Additionally, the researcher had unlimited access to the VR system, allowing for a better grasp of the features available in OpenBrush (2016), and more time to consider how best to represent the descriptions provided by the participants. In Experiment 2, meanwhile, participants had a shorter time to become acquainted with the features of OpenBrush (2016) and provided commentary throughout their time in the VR environment, but in a more sporadic manner. Additionally, as VR can cause motion sickness (Chattha et al., 2020), participants had limited time to complete their drawings. A future experiment could draw on the strengths of both approaches. This could be done by showing the participants the walkthrough video of OpenBrush's (2016) features used in Experiment 1 before the VR session and potentially giving them advance access to the stimuli as well, so that they can think about how they might recreate their synaesthetic experiences in VR. Following the VR session, a semistructured interview could be used to gain clear and detailed feedback on which aspects of their synaesthesia were well captured in VR, and what additional tools, training, or experience they would need to improve the accuracy of their drawings.

### Conclusion

Across two experiments, this study has examined the additional visual experiences of nine synaesthetes. Using the VR package OpenBrush (2016), we aimed to accurately capture the nuances of synaesthetic experiences, with positive results. This has led to a greater understanding and appreciation of synaesthesia for both the researchers and the participants. Furthermore, as an exploratory feasibility study, we aimed to determine whether it is worthwhile pursuing VR technologies as a medium for synaesthesia research.

Participants generally expressed enthusiasm for using VR and stated that they saw potential in this line of research. Additionally, participants stated that participation was a good opportunity to consider details of their experiences that they previously had not noticed or considered in great depth. For example, in Experiment 1, after viewing the introductory OpenBrush (2016) video, Participant 6 remarked that the background should be light in color to match the images she sees, but that she had “never thought about that before.” Furthermore, in Experiment 2, Participant 1 stated that they would like to use the representation to show to their family, emphasizing the potential role of VR depictions as a communication tool.

It is clear that, for some individuals with synaesthesia, both traditional color pickers and two-dimensional art-based software such as Adobe Photoshop are insufficient to represent the complexity of their concurrent experiences. This study demonstrates that VR can be used to depict more elements of a synaesthete's experience, including textural qualities, certain types of movement, and three-dimensional elements, than traditional methods, which therefore helps to promote a deeper understanding of synaesthetic individuals’ unique perceptual experiences.

A final implication is that this novel technique could be used to explore other types of perceptual diversity, including perceptual experiences associated with known conditions such as autism or aphantasia as well as others that might be revealed by ongoing research (Perception Census, 2022).

## Supplemental Material

sj-zip-1-ipe-10.1177_20416695231166305 - Supplemental material for Using immersive virtual reality to recreate the synaesthetic experience Click here for additional data file. Supplemental material, sj-zip-1-ipe-10.1177_20416695231166305 for Using immersive virtual reality to recreate the synaesthetic experience by Rebecca Taylor, Sarune Savickaite, Susanna Henderson, and David Simmons in i-Perception

sj-zip-2-ipe-10.1177_20416695231166305 - Supplemental material for Using immersive virtual reality to recreate the synaesthetic experience Click here for additional data file. Supplemental material, sj-zip-2-ipe-10.1177_20416695231166305 for Using immersive virtual reality to recreate the synaesthetic experience by Rebecca Taylor, Sarune Savickaite, Susanna Henderson, and David Simmons in i-Perception

## Notes

1. Previously TiltBrush (UploadVR, 2021). TiltBrush was produced, created and monetized by Google (2016) and was made available as an open access tool in 2021. It was renamed from TiltBrush to OpenBrush (2016) as a result.

2. Due to the qualitative nature of this study, it was not necessary to quantify sound levels precisely, so long as the two volume levels were discriminably different.

3. Onboarding here refers to a series of processes of how participants are introduced to the VR experiment. In this instance, this involves description of controllers and the headset, explanation of the virtual environment, how to use the tools available and basic navigation in the virtual space.

## References

[bibr1-20416695231166305] Adobe Photoshop (1990). Adobe Inc [Computer Software].

[bibr2-20416695231166305] Adobe Premier Pro (2003). Adobe Inc [Computer Software].

[bibr3-20416695231166305] Audacity (2000). The Audacity Team [Computer Software].

[bibr4-20416695231166305] Baron-Cohen S. Burt L. Smith-Laittan F. Harrison J. Bolton P. (1996). Synaesthesia: Prevalence and familiarity. Perception, 25(9), 1073–1079. 10.1068/p251073 8983047

[bibr5-20416695231166305] BBC News (2021). ‘*Google’s Tilt Brush VR painting app goes open source.’* Retrieved from https://www.bbc.co.uk/news/technology-55826249.

[bibr6-20416695231166305] Billie Eilish Experience (2019). https://web.archive.org/web/20190403020359/http://billieeilishexperience.com/. Last accessed 30/12/2022.

[bibr7-20416695231166305] Braun V. Clarke V. (2006). Using thematic analysis in psychology. Qualitative Research in Psychology, 3(2), 77–101. 10.1191/1478088706qp063oa

[bibr8-20416695231166305] Chattha U. A. Janjua U. I. Anwar F. Madni T. M. Cheema M. F. Janjua S. I. (2020). Motion sickness in virtual reality: An empirical evaluation. IEEE Access, 8, 130486–130499. 10.1109/ACCESS.2020.3007076

[bibr9-20416695231166305] Chiou R. Stelter M. Rich A. N. (2013). Beyond colour perception: Auditory–visual synaesthesia induces experiences of geometric objects in specific locations. Cortex, 49(6), 1750–1763. 10.1016/j.cortex.2012.04.006 22673231

[bibr11-20416695231166305] Eagleman D. M. (2009). The objectification of overlearned sequences: A new view of spatial sequence synesthesia. Cortex, 45(10), 1266–1277. 10.1016/j.cortex.2009.06.012 19665114

[bibr12-20416695231166305] Eagleman D. M. Goodale M. A. (2009). Why color synesthesia involves more than color. Trends in Cognitive Neuroscience, 13(7), 288–292. 10.1016/j.tics.2009.03.009 19525141

[bibr13-20416695231166305] Grossenbacher P. G. (1997). Perception and sensory information in synaesthetic experience. In Baron-Cohen S. Harrison J. E. (Eds.), Synaesthesia: Classic and contemporary readings (pp. 148–172). Blackwell Publishing.

[bibr14-20416695231166305] Hacmun I. Regev D. Salomon R. (2018). The principles of art therapy in virtual reality. Frontiers in Psychology, 9. 10.3389/fpsyg.2018.02082 PMC6220080 30429813

[bibr15-20416695231166305] Lacey S. Martinez M. Steiner N. Nygaard L. C. Sathian K. (2021). Consistency and strength of grapheme-color associations are separable aspects of synesthetic experience. Consciousness and Cognition, 91, 103137. 10.1016/j.concog.2021.103137 33933880 PMC9296080

[bibr16-20416695231166305] Lee J. Kim J. Choi J. Y. (2019). The adoption of virtual reality devices: The technology acceptance model integrating enjoyment, social interaction, and strength of the social ties. Telematics and Informatics, 39, 37–48. 10.1016/J.TELE.2018.12.006

[bibr17-20416695231166305] Mahrholz G. Belin P. McAleer P. (2018). Judgements of a speaker’s personality are correlated across differing content and stimulus type. PLoS One, 13(10). 10.1371/journal.pone.0204991 PMC6171871 30286148

[bibr18-20416695231166305] Mandal S. (2013). Brief introduction of virtual reality & its challenges. International Journal of Scientific & Engineering Research, 4(4), 304–309.

[bibr19-20416695231166305] Melatti M. Johnsen K. (2017). Virtual Reality mediated instruction and learning. 2017 IEEE Virtual Reality Workshop on K-12 Embodied Learning through Virtual & Augmented Reality (KELVAR), 1-6. https://doi.org/10.1109/KELVAR.2017.7961556.

[bibr20-20416695231166305] Moos A. Simmons D. Simner J. Smith R. (2013). Color and texture associations in voice-induced synesthesia. Frontiers in Psychology, 4, 568. 10.3389/fpsyg.2013.00568 24032023 PMC3759022

[bibr21-20416695231166305] Neufeld J. Sinke C. Dillo W. Emrich H. N. Szycik G. R. Dima D. Bleich S. Zedler M. (2012). The neural correlates of coloured music: A functional MRI investigation of auditory–visual synaesthesia. Neuropsychologia, 50(1), 85–89. 10.1016/j.neuropsychologia.2011.11.001 22093438

[bibr22-20416695231166305] NVivo 12 (2020). QSR International Pty Ltd [Computer Software].

[bibr23-20416695231166305] OpenBrush (2016). Google [Computer Software].

[bibr24-20416695231166305] Perception Census (2022). https://perceptioncensus.dreamachine.world/. Last accessed December 31, 2022.

[bibr25-20416695231166305] Queiros A. Faria D. Almeida F. (2017). Strengths and limitations of qualitative and quantitative research methods. European Journal of Education Studies, 3(9), 369–387. https://doi.org/https://10.5281/zenodo.887089

[bibr26-20416695231166305] Rendall D. Kollias S. Ney C. (2005). Pitch (F0) and formant profiles of human vowels and vowel-like baboon grunts: The role of vocalizer body size and voice-acoustic allometry. The Journal of the Acoustical Society of America, 117(2), 944. 10.1121/1.1848011 15759713

[bibr27-20416695231166305] Rich A. N. Bradshaw J. L. Mattingley J. B. (2005). A systematic, large-scale study of synaesthesia: Implications for the role of early experience in lexical-colour associations. Cognition, 98(1), 53–84. 10.1016/j.cognition.2004.11.003 16297676

[bibr28-20416695231166305] Rothen N. Seth A. K. Witzel C. Ward J. (2013). Diagnosing synaesthesia with online colour pickers: Maximising sensitivity and specificity. Journal of Neuroscience Methods, 215(1), 156–160. 10.1016/j.jneumeth.2013.02.009 23458658

[bibr29-20416695231166305] Savickaite S. McNaughton K. Gaillard E. Amaya I. McDonnell N. Millington E. Simmons D. (2021). Using HMD Virtual Reality to investigate individual differences in visual processing styles. *PsyArXiv Preprints.* 10.31234/osf.io/g7d9c.

[bibr30-20416695231166305] Simner J. (2011). Defining synaesthesia. British Journal of Psychology, 103(1), 1–15. 10.1348/000712610X528305 22229768

[bibr31-20416695231166305] Simner J. Carmichael D. A. (2015). Is synaesthesia a dominantly female trait? Cognitive Neuroscience, 6(2-3), 68–76. 10.1080/17588928.2015.1019441 25732702 PMC4566887

[bibr33-20416695231166305] Simner J. Mulvenna C. Sagiv N. Tsakanikos E. Witherby S. A. Fraser C. Scott K. Ward J. (2006). Synaesthesia: The prevalence of atypical cross-modal experiences. Perception, 35(8), 1024–1033. 10.1068/p5469 17076063

[bibr34-20416695231166305] Simner J. Ward J. Lanz M. Jansari A. Noonan K. Glover L. Oakley D. (2005). Nonrandom associations of graphemes to colours in synaesthetic and non-synaesthetic populations. Cognitive Neuropsychology, 22(8), 1069–1085. 10.1080/02643290500200122 21038290

[bibr35-20416695231166305] Sollberger M. (2011). Rethinking synaesthesia. Philosophical Psychology, 26(2), 171–187. 10.1080/09515089.2011.627539

[bibr36-20416695231166305] Spence C. (2011). Crossmodal correspondences: A tutorial review. Attention, Perception & Psychophysics, 73, 971–995. 10.3758/s13414-010-0073-7 21264748

[bibr37-20416695231166305] Steam (2003). Valve Corporation [Computer Software].

[bibr38-20416695231166305] Steen C. (2001). Visions shared: A firsthand Look into synesthesia and art. Leonardo, 34(3), 203–208. 10.1162/002409401750286949

[bibr39-20416695231166305] Taberham P. (2013). Correspondences in cinema: Synaesthetic film reconsidered. Animation Journal, 21, 47–68.

[bibr40-20416695231166305] Taggart E. (2019). ‘*5 Synesthesia Artists Who Paint Their Multi-Sensory Experiences’*. Retrieved from https://mymodernmet.com/synesthesia-art/.

[bibr41-20416695231166305] Vive (2016). HTC Vive [Computer Software].

[bibr42-20416695231166305] Wagler A. Hanus M. D. (2018). Comparing virtual reality tourism to real-life experiences: Effects of presence and engagement on attitude and enjoyment. Communication Research Reports, 35(5), 456–464. 10.1080/08824096.2018.1525350

[bibr43-20416695231166305] Ward J. (2019). The co-occurrence of mirror-touch with other types of synaesthesia. Perception, 48(11), 1146–1152. 10.1177/0301006619875917 31530082

[bibr44-20416695231166305] Ward J. Huckstep B. Tsakanikos E. (2006). Sound-colour synaesthesia: To what extent does it use cross-modal mechanisms common to us all? Cortex, 42(2), 264–280. 10.1016/S0010-9452(08)70352-6 16683501

[bibr45-20416695231166305] Ward J. Moore S. Thompson-Lake D. Salih S. Beck B. (2008). The aesthetic appeal of auditory-visual synaesthetic perceptions in people without synaesthesia. Perception, 37(8), 1285–1296. 10.1068/p5815 18853563

[bibr46-20416695231166305] Ward J. Simner J. (2020). Chapter 13—synesthesia: The current state of the field. Multisensory Perception, 283–230. 10.1016/B978-0-12-812492-5.00013-9

[bibr47-20416695231166305] Weinel J. (2021). Synaesthetisc Audio-Visual Sound Toys in Virtual Reality. *AM'21: Proceedings of the 16^th^ International Audio Mostly Conference*, 135-138. 10.1145/3478384.3478400.

