# Supplementary material for: Using immersive virtual reality to recreate the synaesthetic experience
Source: Iperception. 2023 Sep 4;14(3):20416695231166305. doi: 10.1177/20416695231166305 (PMC10478570; doi:10.1177/20416695231166305)
Supplement: sj-zip-2-ipe-10.1177_20416695231166305 - Supplemental material for Using immersive virtual reality to recreate the synaesthetic experience [file sj-zip-2-ipe-10.1177_20416695231166305.zip › Transcripts/SN1.docx]

Experimenter: Imagine this kind of stuff, I just need a plain white background or something. This is better. Doesn't it look too bright? Best one yeah. And then these are the colors. And then if you try the brushes, I think you must have tried a few. But if you want to try some more - the ones that look funky and you haven't yet.

Participant 1: I, I mean, I haven't yet. Trying different ones. Oh, OK.

Experimenter: This is how to make them bigger and smaller. So on your right hand there's a similar round button that you normally use.

Participant 1: Oh yeah.

Experimenter: Extend it or so you see.. now it's bigger. Yeah, so it's kind of you just sort of swipe it in a similar way and then it makes it smaller. And then you can teleport from the ones you've drawn so you. Don't have to delete. Them you can just press teleport.. yeah and then press somewhere far in. space and I have new blank canvas because. Don't have to keep deleting things and get distracted. But you need to turn off teleportation before you do anything else. And try different funky versions. See if you like them. Try different ones. If there's anything specific with movement.

Participant 1: I like that.

Experimenter: If you put it above your above your head press it, it ;ooks like it's snows. And then if you leave it, it just keeps dropping.

Participant 1: This is crazy, it does. I don't think it matters how long I would spend in here. It would never stop like amazing me.

Experimenter: Right?

Participant 1: It's really fun to do, especially yeah … 'cause once you kinda have some time to play around then I will get there.

Experimenter: Test audio set up and then we'll do the actual 5 sounds.

It seems to be glitching. I don't know what it is. Do you want me to restart?

[technical issues]

Participant 1: Yeah, that's fine.

Experimenter: That makes sense. Is there any particular type of? Sounds that trigger your synesthesia?

Participant 1: It's really quite difficult to describe that... I've got sensory adaptation to it, it's less easy to pick up on like my synesthetic reactions to the stimulus, but it's still there.

Experimenter: OK, yeah.

Participant 1: Feeling a lot. Of things yeah. But I'm not really as aware of it and I get really strong reactions to the sound of Velcro.

Experimenter: OK. Oh really, OK.

Participant 1: Food is a really strong one 'cause I also work as a chef.

Experimenter: Is it like just is it someone chewing in a sense? Or is it more like just preparation food?

Participant 1: and it can sometimes be because I'm trained in knife skills. So when I'm preparing veg and I can have a lot of sort of reactions to it and I can see a lot of colors from the sound of the blade or the smell of the vegetable.

Experimenter: So the smell is also what kind of? Adds on to that.

Participant 1: Yeah, because all of my reactions are sort of cognitive and like colors and waves or things that I can sort of cognitively see in the same way that you can see a memory and so you kind of see it in the minds eye as well. I can in that way I can see, smell, touch and sound.

Experimenter: OK.

Experimenter: Let me try to play some of the sort of sounds to see how it works with the volume.

Participant 1: Very well. That's OK.

Experimenter: So let me just play some. Something that's not too startling .

Participant 1: OK.

Experimenter: It should be coming through now, can you hear?

Participant 1: That should be.

Experimenter: Fine, is it OK sound wise? Because some of the sounds we gonna have they're bit more subtle. Yeah, so kind of keep that in mind. So maybe I can you can draw to this for a bit, or do you want me to jump into the stimuli straight away?

Participant 1: Yeah, let's do some drawing. Maybe just to start with.

Participant 1: OK, so usually. The best way for me to kind of... My experience is like... for my friends and my family before so usually my best way of doing it is by it's like a shape tool. So my best way of doing it is by sort of like just. I know. This specific color. Yeah, so if I start with just drawing. As sort of my visual field. And ignore everything outside the box. And so I'm going to have to split this into different sections.

Experimenter: OK. Just talk us through it.

Participant1 : And I can hear quite specific shape. So because basically there's a few different things going on, and I see different things for each thing, each layer to this sound so I can hear that the guitars got some reverb. And if you imagine this just as block color. Yeah, within the square. And it will just be like that. OK and.. It's in this color. And a lot of my synesthesia follows like a similar pattern, and with these kinds of sounds.

Experimenter: OK.

Participant 1: So there's usually some kind of like sort of sideways eye shape in the middle and so I don't have the best brush.

Experimenter: What sort of what you're looking for? Is this animated? Do you know the smoke brush? … You can delete it entirely. But you might need to make it bigger. It will be a little animated.

Participant 1: So then from the middle. There will be a lot of stuff like this, in fact, that's perfect, yeah.

Experimenter: Is it? So like it quite misty.

Participant 1: Yeah, and but it's almost. It's a little bit like and you know when you hold your hands onto your eyes for too long and you're going to weird like vortex or... I'll just put some here and but it does have a pretty good gradient to it.

Participant 1: So basically from the center here everything is pulsating outwards as if it's churning round, yeah. But I can't obviously, draw that, so that would be sort of the reverb and it's not gonna work.

Experimenter: So there are some sort of brushes that kind of do movement, but they're not necessarily smoke like.

Participant 1: Yeah, I mean this smoke will actually come in really helpful because a lot of my synesthesia looks kind of like that. Just 'cause there's quite a lot going on.

Experimenter: Yeah, yeah, that's good. Almost you can distinguish individual instruments like layers?

Participant 1: My answer would be a little bit biased 'cause I can play several instruments and so I'm a bit tuned to separate, you know, sounds in audio streams. It's not so clear cut, so like that that I've just drawn is the general reverb across all instruments. But then I can separate. The sound of the acoustic guitar and the electric guitar that comes in and they both look very different.

Experimenter: Oh, OK.

Participant 1: It sounds just wrong.

Experimenter: So do you think it's linked to the nature instrumentals or specifically notes, or is it neither?

Participant 1: It's it's a variety of sort of the whole construction of the instrument, and because, for example, I can sort of see a lot of the texture of the strings. I can see when they're getting plucked. And and it's stimulating quite a lot, but if I was hearing a much more grainy and overdriven guitar, I'd see a lot more sort of fuzzy images in my head and so I'll go back to smoke brush actually.

Experimenter: The further down you go. Onto the right, the funkier the brushes get, the more animated they get.

Participant 1: Yeah, I don't want that one... So electric one is this little bit moved. That is very important.

Experimenter: OK.

Participant 1: 'cause sometimes it's not so easy to draw what I see. Sometimes it will sort of feel a little bit like this and I don't really get much else.

Experimenter: OK.

Experimenter: Yeah, a few brushes are animated, so hopefully maybe they'll help. So when you draw experiences for like your family, etc. do you normally do it on paper? And I mean it doesn't come up as often as you'd think it would.

Participant 1: Yeah, I mean well, I'm quite lucky because it got spotted with me when I was quite young and I got diagnosed with autism when I was about six or seven years old. So it's been known for quite a while. And my mom works as a psychologist.

Experimenter: Oh, OK.

Participant 1: And which is quite helpful 'cause she in the past has tried to help me, sort of. Draw my synesthetic experiences a bit more. Just cover all this..

Participant 1: OK, so we have this like pulsating background and then it's kind of like that one. This is more like an electric. So in acoustic guitar it's usually the same. I can draw the notes that the. We've got guitar plays and different from the sound of the strings itself.

Experimenter: Do you think that makes it easier for you to do the different notes, or would you think that's just something that you've always been able to do? Able to differentiate with colours?

Participant 1: and I do think it makes it easier. 'cause I've never had a music lesson and I play almost entirely by ear.

Experimenter: So did you just taught yourself basically? Do you have perfect pitch?

Participant 1: Ah no, no, I can get a little bit wibbly wobbly here and there just because I'm not classically trained, so I'm not always right and you can't see this.

Experimenter: So you always think in two dimensions so far. What if you think of it as three-dimensional because you can do it in VR. So you define yourself with barrier here. But if you're wanting to something to come forward you can draw something in planes. If you look sideways it would be dimensional so it's slightly different way of thinking because it's not something you normally get to do, but maybe that would help you represent it better if you drew a plane of this pulsation. Can see those like a cloud.

Participant 1: Yes, it's... That is really difficult. Just because how faint the experience is. When I have it is very difficult to imagine it in a multi dimensional sort of way, and because it stays within your field of vision, so kind of overlaps over the dimensionality of the world.

Participant 1: But usually what would happen is with acoustic guitar strings... This would be sort of a metallic bar going across and this would kind of vibrate up and down. Yeah yeah, and it would change.

Experimenter: OK.

Participant 1: In size and get thicker and thinner.

Experimenter: Oh OK, I see.

Participant 1: Yeah, I mean this is the best I've ever gotten to be honest.

Experimenter: Really OK.

Participant 1: Yeah, it's true.

Experimenter: And then maybe we can try to do the five sounds just to see how you feel about those 'cause they are little simplistic in the sense that it's just one specific sound - everyday sound, so I'll play one and I can play it as many times as you want. Just let me know.

SOUND 1

Participant 1: Oh my God... Yeah, this is. Pretty much. This is the best I've ever seen that can describe the sort of stars in your eyes.

Experimenter: That's the bird sort of singing.

Participant 1: Yeah, yeah, that's kind of what it was. Like and it's all moving exactly. And it's always like the same kind.

Experimenter: Of color yeah? Do you think like different birds will be different color or different experiences?

Participant 1: Yeah yeah, like seagulls make like quite a yellow sound and it's quite a bold block color rather than anything that sort of moves and crows make a sort of orange sound and it's a little bit like a texture of sandpaper.

Experimenter: Ah, interesting.

Participant 1: But because I imagine they must be small, but it's like a Robin or whatever. Yeah, they all kind of get branded like this. In my mind.

SOUND 2

Experimenter: Does this sound OK? I'll replay that real quick.

Participant 1: It's another best way to relate this... Like specific lines going through.

Experimenter: Would they be somehow in space, do you think?

Participant 1: Yeah, they would kind of.

Experimenter: Did they bend towards you?

Participant 1: No, they move right to left.

Experimenter: OK.

Participant 1: Yeah, but. There'd be millions of these. I mean, this would almost be a, you know it... Would be like a thing. It would be like this. The embers would still be going over the top as well. You can see the embers through it. Yeah, and the bottom bit with smoke. Yeah, it's just a little bit fuzzy.

Experimenter: Oh, OK.

Participant 1: It's a bit like, you know when a static television and loses signal just at the bottom of like my visual field, it's just... Yeah, and it has quite a defined color as well.

Experimenter: Is it always kind of like? A blueish?

Participant 1: And yeah, there are different shades of blue because these, like electric bits. Here are a little bit lighter. And then there's some. And and it's just kind of like this. And they will just sort of, you know... like on a diagram of different sort of electromagnetic waves, they're all at different wavelengths, yeah? And they will moving from left to right, and these, and moving from top right towards sort of mid left.

Experimenter: Does this the movement coincide with the car is sort of moving away, and because it's kind of suggested that it's like going past you, the car does that somehow coincide with it?

Participant 1: I think it's it's possible I I don't understand it. Yeah, I listen to find that sounds that are. Affected by movement will have more. More sort of kinetic.

Experimenter: OK.

Participant 1: 'cause I do a lot of cycling and when I feel the feeling of my chain on the bike between my pedals and that makes me think of like gray sort of all rocks like sort of grinding against each.

Experimenter: Ah yeah, yeah.

Participant 1: They will sort of.. move and that's because it's a very connected with sound. Yeah, I guess.

SOUND 3

Participant 1: Oh, this is easy.

Experimenter: Oh really, yeah.

Participant 1: It's a similar way like you said, with the music. It was sort of the corner where for the... Yeah, it's like this diagonal shape going across. I've heard sounds like this way too many times too, so it's like this is a very common.

Experimenter: OK.

Participant 1: Experience I've had.

Experimenter: If you want me to play it again, let me know, but it's not the most pleasant of sound.

Participant 1: It's quite important that there's like this sort of circular motion.

Experimenter: Is the motion quite as frequent as this brush? Or is it a bit slower?

Participant 1: And in in the middle it's slower, but on the outside it moves a lot more, almost like a sort of gravitational vacuum.

Experimenter: Does it get distracting?

Participant 1: Yeah it can make me very over stimulated.

Experimenter: Noises like this?

Participant 1: Very blood splatter kind of thing. We need to just make it a little bit. color matched.

Experimenter: And the colors normally quite like pastel? Or is it like ever like really bright, bright, bright?

Participant 1: It does get bright and that's mostly when I listen to really textured sort of music where there's a lot going on.

Experimenter: OK.

Participant 1: And like I'm a big metal fan, and that's primarily because when I listen to metal there's so much going. On that, it just looks really nice and a lot of my sort of tastes in music are more around how it looks rather than how it sounds.

Experimenter: I see.

Participant 1: And how it makes me feel as a consequence? See, it's kind of like this, but massive. So see how big I can get it.

Experimenter: Yes, it's. Seems to follow the same sort of shape, again like that sort of eye shape like you said.

Participant 1: And this is all very blotchy.

Experimenter: OK.

Participant 1: There are in reality.. there were less fewer gaps, but I can see phasing circles in the middle, which is why that's quite good.

Experimenter: Yeah, yeah.

Participant 1: But that is almost pitch perfect. How it works?

Experimenter: Well, we've got two more sounds, so not too long. And so we've got rain next. So I'll play that. I can replay it a few times if you need to. I'll just wait till you draw the boundary.

Participant 1: And I'm sorry it's really important. It's easier to sometimes draw with them, but. Yeah, and it's kind of how I experience it.

SOUND 4

Experimenter: Can you hear OK?

Participant 1: OK. That's quite cool. That's a bit different than smoke, it's more clear. Yeah, so this is much more vivid in the background. And there are like these... Yeah, OK. And then this. So the darker colored... couldn't get that to go darker.

Participant 1: Darker than the bottom. Blue, or about the same darkness.

Experimenter: This color OK?

Participant 1: And then like a block color. Like a bold color.

Experimenter: So not really textured?

Participant 1: They move in this way, but they're solid circles like there's no like reflection or textures.

Experimenter: Yeah, yeah. Yeah, are they solid as in flat or like? Round, as in like bubble.

Participant 1: No, yeah, that's that's solid, like 2D.

Experimenter: OK.

Participant 1: And and then this. Well, it seems a bit small though. Yeah, that's yeah it's not right.

Participant 1: OK, that's perfect. And then there's... and these move really slowly and with me waving them. That's just to show that they kind of move in a very solid way. They don't sort of... Make sure we move like rain coming from a cloud, they just this is just sort of a stream.

Experimenter: Yeah, yeah.

Participant 1: This gently moves.

Experimenter: Is it just from that one side? OK.

Participant 1: And this like. So I'm just in the corner of my eye. It's just a little bit of this here, and it's about that color. OK, just kind of comes out from the corner. Yeah, that's about it.

Experimenter: Cool well one more to go. Is that too loud? If it's too loud, you can turn it down 'cause I can hear it. Do you want me to turn it down?

Participant 1: Yeah, it's fine. This is going to be hard.

Experimenter: OK, how so is it?

Participant 1: Just going to populate this area loads.

Experimenter: If you want me to play that can. Play it back. But it's not the most nicest sound. Let me know if you do want it.

Participant 1: I think it should be OK just now, yeah?

Participant 1: OK, and it's always the same sort of direction. Yeah, it's it's left to right.

Experimenter: OK yeah yeah.

Participant 1: Well, yeah, I think that's about it.

Experimenter: That's it, yeah, OK. Cool, well, unless you want to draw Something for your fun. It's basically just experiment and I just got a few more questions and. I don't want. To keep people in the VR for too long because you kind of get disoriented.

Participant 1: Oh wow, yeah.

Participant 1: I mean I really enjoy. That because it's. Well, to be honest, I never got to represent my experiences so clearly. I would like to show it to my family.

Experimenter: How did you find VR for representing your perceptual experiences?

Participant 1: very good.

Experimenter: Better than pen and paper?

Participant 1: yes, for sure.
