# Supplementary material for: Using immersive virtual reality to recreate the synaesthetic experience
Source: Iperception. 2023 Sep 4;14(3):20416695231166305. doi: 10.1177/20416695231166305 (PMC10478570; doi:10.1177/20416695231166305)
Supplement: sj-zip-2-ipe-10.1177_20416695231166305 - Supplemental material for Using immersive virtual reality to recreate the synaesthetic experience [file sj-zip-2-ipe-10.1177_20416695231166305.zip › Transcripts/SN2withimages.docx]

Experimenter: So if you want to move away, you can just move away from it and start anew. You can move sort of forward a bit and to the right. If you do want to physically move, but. That's the safe place.


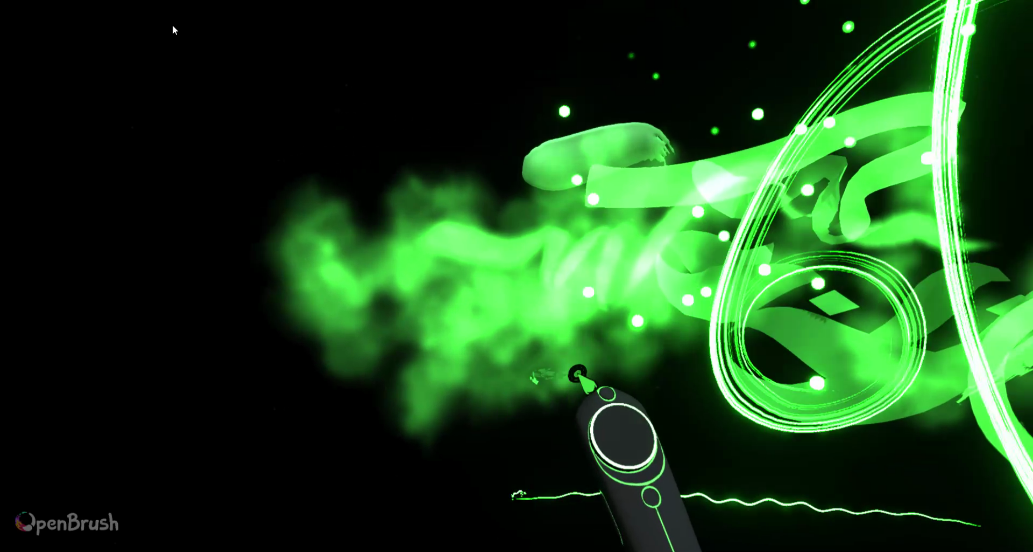
Participant 2: That's weird. Oh wow.

Experimenter: Remember you in 3D space. So you can look at these from a size and you have dimensionality, so that's sometimes a hard one to do to imagine things in 3D 'cause you always still make it very 2 dimensional.

Participant 2: Yeah, definitely.

Participant 2: It looks like a little worm when it's green.

Experimenter: It is just really discovering the ones you like? And then they stick with the ones that they think it's all closest.

Participant 2: Yes, I liked wire. And this one was quite good as well. Some other ones. Were just sort of more like this. One is more like a... It creates some sort of a shape.

Experimenter: That's great.

Experimenter: This really depends on like what do you think is the best way that represents your visual experience. 'cause everyone got different things. I don't want to share what other people do.


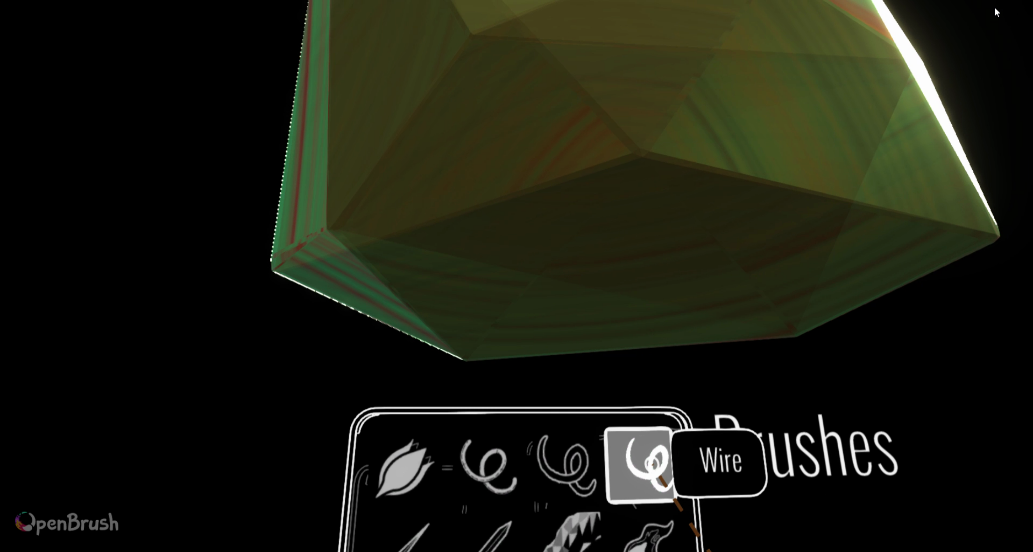
Participant 2: Yeah, Oh yeah, definitely. OK, OK I think I. Know which ones they are.

Experimenter: So now we'll just have some test music. Sort of a little mellow music. Nothing too fancy, but it's mostly just to get you to get used to sound and mostly just to get the volume right. OK. And then. Can you hear it?

Participant 2: I can yeah.

Experimenter: Is it OK? And feel free to tell what you like to do if you want, but you don't have to speak. OK, just if that helps to sort of illustrate what you're seeing and.

Participant 2: So like usually with music like this, like this artistic, it's very yellow and orange, so I've done like a big yellow shape and then like a sort of orange line in the middle. But there's like other things coming through as well so. Like the little. Guitar scratches are kind of silver, so.


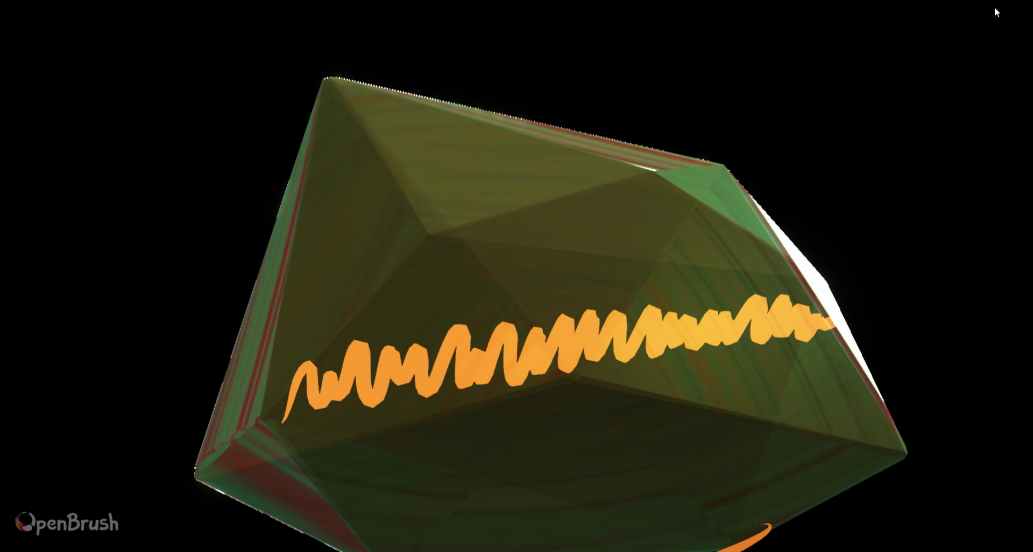
Experimenter: Do identify individual instruments by the colors?

Participant 2: Sometimes, and it kind of depends on how they're used. And a lot of songs that I like, uh, share like characteristics. OK, I'm gonna add some red 'cause there's like an electric guitar coming in.

Experimenter: Does it have any movement?

Participant 2: With music it tends to have like a beginning and an end, so it's like a journey. OK and then. Sometimes it can deviate. This is like an acoustic piece, so it sort of... It doesn't have too many like branches.

Experimenter: OK.


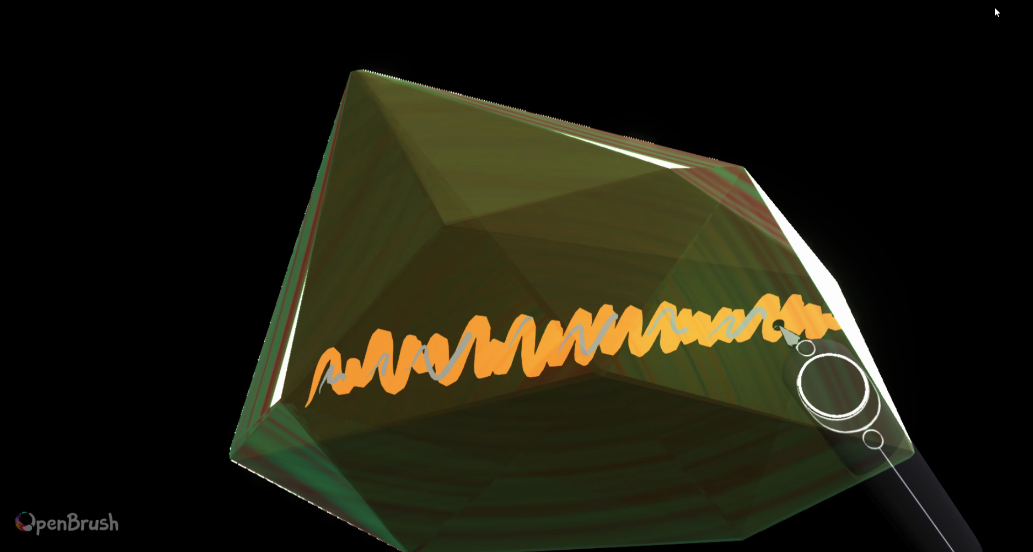
Participant 2: … like a traditional song with like verses and choruses then it sort of has like different shapes coming out of it depending on like what parts match together. So yeah, it's kind of like... I always see music. I actually draw it more on the ground. But it kind of follows a journey. Yeah, like this is very yeah.. this is very much like a still me thinking in like a 2D .. it's like I'm standing on it and I kind of like walk through it so.

Experimenter: We can't replay it again? I was gonna ask something - is it always little contained within some sort of a space that it's not just floating? Is it some sort of like boundaries?

Participant 2: I don't think it has boundaries and so. It's hard, it's hard to describe so.

Experimenter: See if you can draw it... oh, so flat like you as if it was on the floor.

Participant 2: It's kind of like a path. But I'm trying to sort of like bring things up and down as. Well OK, so some of … is like really small for some reason, but like again also sort of like.

Experimenter: And is guitar always the same sort of color?

Participant 2: No, again it depends... this is a really warm sound and it's quite round and yeah, it's like very orange.

Experimenter: OK.

Participant 2: … like electric guitars. Red and like this sort of like noodling... Big chunky orange and yellow and shades. You hear like a little scratch, so it's almost like... All these little pieces. And then sometimes, like I do listen to quite a lot of a rock music. So and it depends on like the type of song, but sometimes it can be like blue, the guitar can be like broken blue, and it depends on the mood. And the key and the tone that they're going far. OK... A bit less warm and a bit less rounded, it's kind of harder and a bit colder.

Experimenter: So is it the combination of keys and notes? Or is it more like the different expressions.


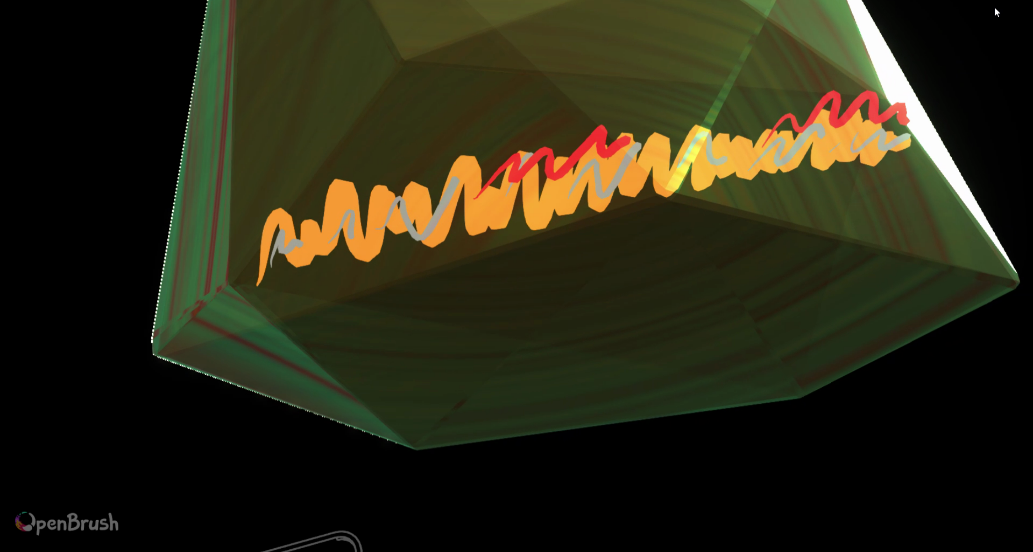
Participant 2: A little bit of both... and then again also if there's any vocals like they also have an impact on it as well. OK, and because sometimes the vocals can change the direction, so like quite often with songs like it's kind of like fully in one Direction and then we've turned.

Experimenter: Right?

Participant 2: Sometimes there's a difference in. Like the beat in the background or you can just like feel there's a different pace to the song so it turns a corner, but yeah... So with this one I was kind of like... OK so to begin with we have like the warmth of the guitar playing and little scratches... once the electric guitar starts - then it started the corner. Yeah, it's like this... a new sound being added and it's sort of like new section of the song. I used to play a trumpet when I was younger. So I also used to like look at a little sheet music. And that was something that yeah... I would break into little sections. OK, so that's part were going forward and then this part was different, so it was like turning a corner again and going in another direction. And then sometimes you can't come back, but... And yeah, it's kind of a journey through.

Experimenter: are all the songs the same? so the same song always stays consistent?

Participant 2: Yeah.. It always has the same shape. I may add things like yeah.. So like you know how sometimes when you listen with headphones here more than when you listen with the speaker and I'll hear something and I'll be like, oh.

Experimenter: Yeah, yeah.

Participant 2: That's the whole thing. That comes into this and and like gives it some embellishment a little bit.

Experimenter: Yeah, interesting. OK, so if you teleport away somewhere new. The first one and it will be just birds, so it shouldn't be too loud. If it's too uncomfortable or whatever else let me know. And we can replay.

**SOUND 1**

Experimenter: I can hear it?


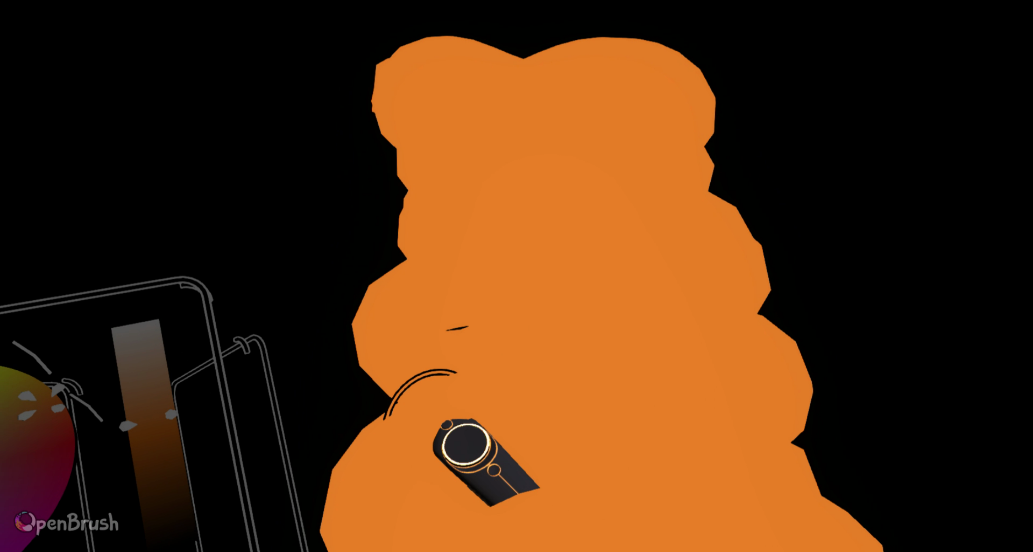
Participant 2: There's quite smokiness of... Yeah, it's kind of like cloudiness and it's also up high like rather than something I'm standing on. Move through it like I could do that here.

Experimenter: It doesn't represent better do you think? When you can ,ove through it rather than like having to draw some pen and paper.

Participant 2: Yeah, and because I've always like have tried to draw things for people and like trying to describe how it's about .. standing on it.

Experimenter: Yeah, yeah.

Participant 2: And being inside it as well, so as well as like sounds. And I do this with like time and numbers and ages. So the age that I am at the moment, I am on this map. Where I am at 31. And everybody else I know is like placed across the map.


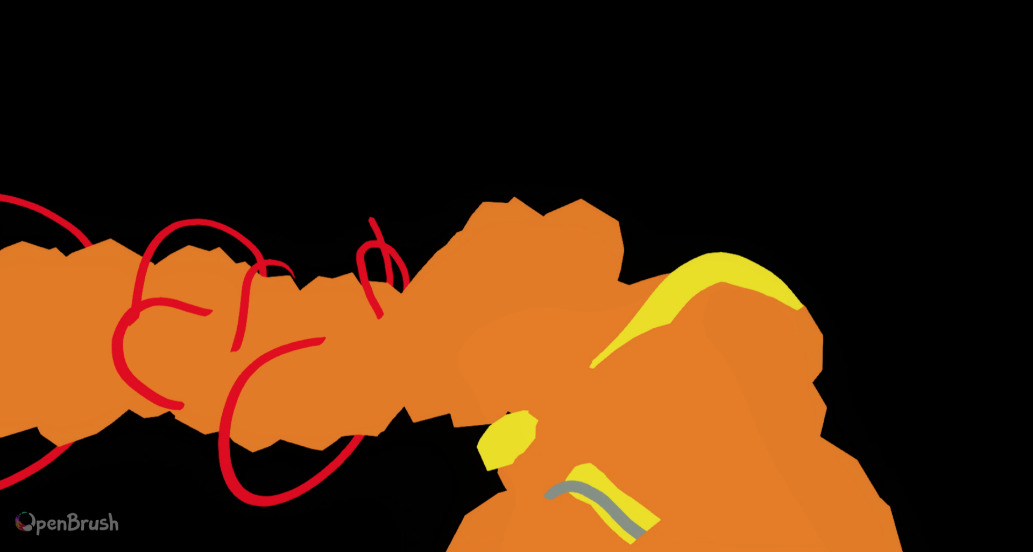
Experimenter: Right?

Participant 2: But the year is a is an anticlockwise ring for me, and again, I'm always in a month and looking at the rest of the year so. And yeah, when I draw it for people, I'm like this... It's not a Birds Eye view. It's being in the middle of it that you actually get to see.

Experimenter: There's mostly smoke in this case.

Participant 2: Oh yes. There's just like. Little dots, Yep, there's like little airborne speckles. And then I can... Obviously, like the bird, some has like a pattern to it and so the one at the end is.

[technical issues]


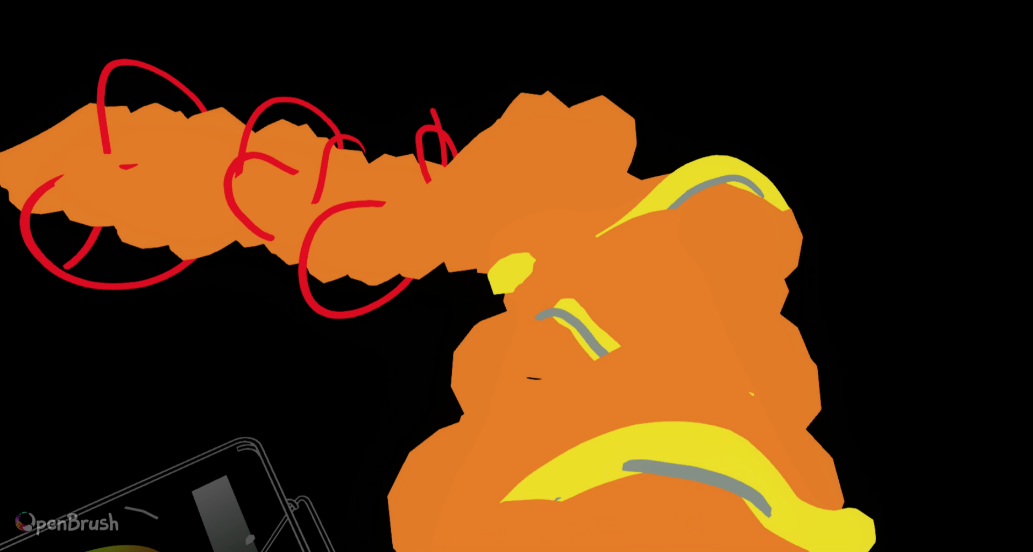
Participant 2: I should be small. Sounds like little peaks... Like this is the closest that I can describe the sound like this. This represents the sound so green cloud is almost like the overall color. And shape that I get from it and then the little brown speckles are kind of almost like they are birds. Very much feels like how is that how it feels when listening to them. And again, this is about like the journey. So yeah from beginning to end you're like walking through.

Experimenter: OK, yeah, this is so fun. We can send the video to you if you want.

Participant 2: Oh, this would be cool

Experimenter: Do different bird sound different?

Participant 2: That's a difficult question. Just take a sort of like this little brown dot looking like the cloud is kind of representative of like a different bird, even though it doesn't distinguish it. But that's also. Because of my lack of knowledge on birds... Yeah, it's just 'cause these are more like songbirds or something like a crow or seagull is very distinctive. It's not different color or but it's still there, made kinda like the yellowish green.

Experimenter: OK.

**SOUND 2**


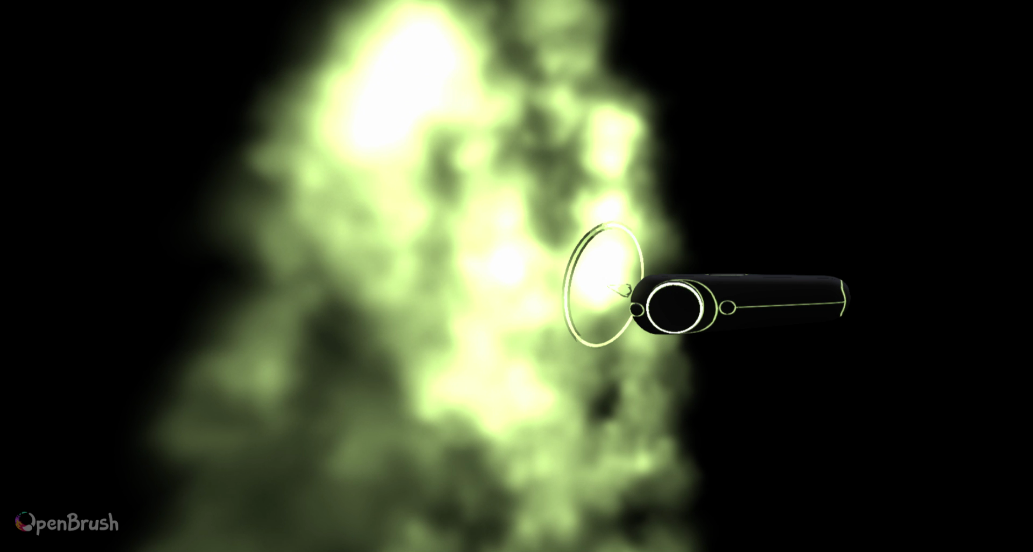
Participant 2: I sort of link with like their colors so the crow could be black, but it has like a very like serrated edge to it because of how they sound.

Experimenter: OK. cool, well we move the next one. You can teleport away. And then the next sound. So you can listen to it first, and then I'll replay a few times as you do it. And let me know if the volume is ok.

Participant 2: Can we turn it up? A little.

Experimenter: You getting anything at all?

Participant 2: All I'm hearing is very fast. OK, so it might be some of them are slightly different so... Yeah, it it's quite helpful to to hear back. It seems...

Experimenter: Is this one harder than the birds?

Participant 2: Things within it that it's sort of like..

Participant 2: Let's get some moving out in space. Oh yeah. Like a 3D element.

Experimenter: So this one is flat?


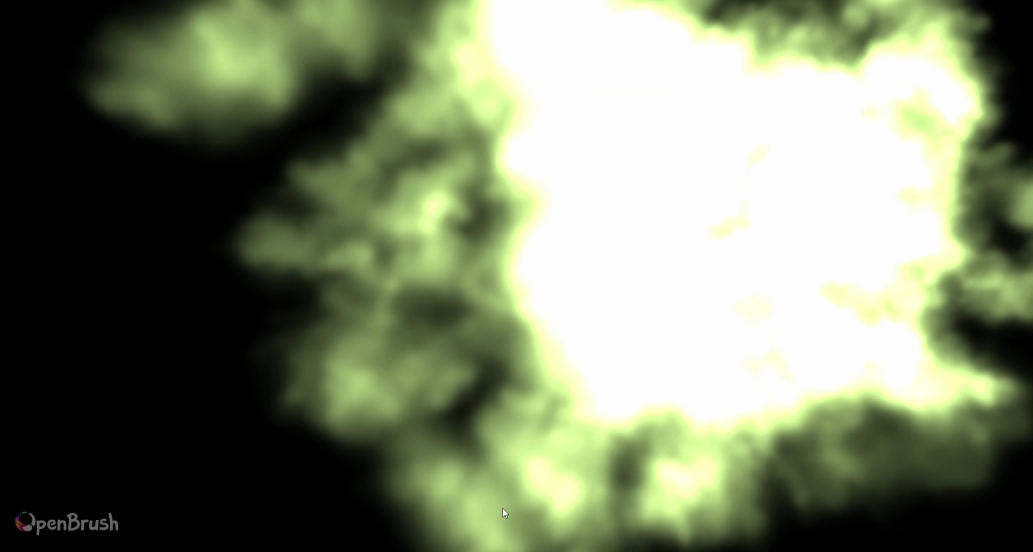
Participant 2: I think so. It looks like, yeah... OK, that's great. Good for making shapes. That one is gonna be better. OK yeah that one is there. So it's sort of like an arrow at the end and then.. look like a blimp. And it's got this big color 'cause it sounded quite a cold and wet. And I think it genuinely just reminds of rainy day and being on the street.

Experimenter: This is going to be probably small, yeah?

Participant 2: So it's got like a sort of stony rough texture.

Experimenter: Ah, OK.

Participant 2: Yeah, it's like really cold colors. Looking for it's kind of scratched so.

Participant 2: OK, yeah.

Participant 2: It's trying to do something that kind of looks like that maybe just like … Yeah, maybe just very small brush. Very sharp. That's probably small. That's type of brush that's probably not quite there.


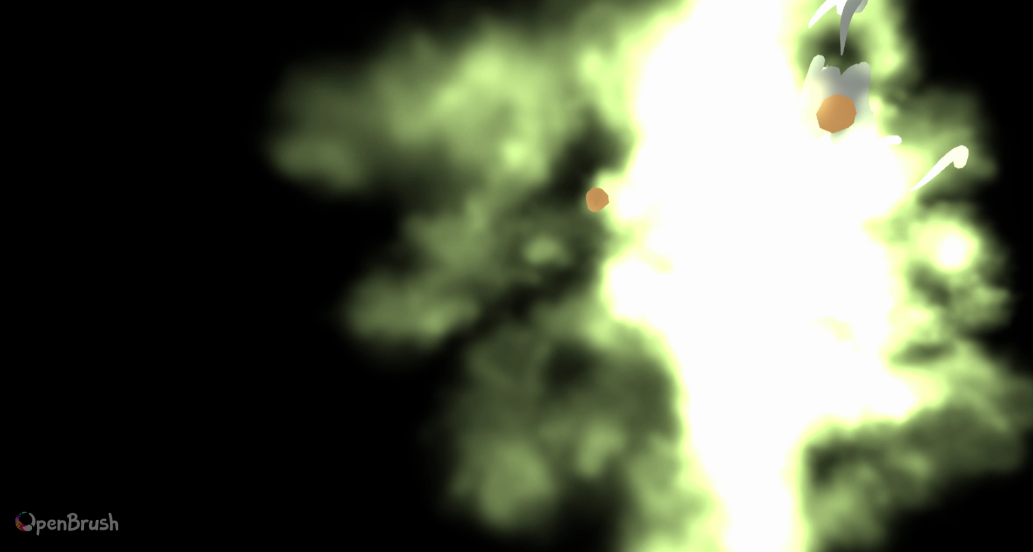
Experimenter: it might not be there [the brush] but use this for illustrative purposes.

Participant 2: Yeah, so it's like very scratched. Yeah, so it's. Got these this sort of like. Bumpy rocky texture and like if you took on your hand over there it would be like really rough.

Experimenter: Like a story?

Participant 2: Yeah, and so it starts here and grows and then goes away again.

Experimenter: OK, kind of like the car passing by. Right?

Participant: it's very accurate..

Experimenter: Do you want to spend more time on this or move on?


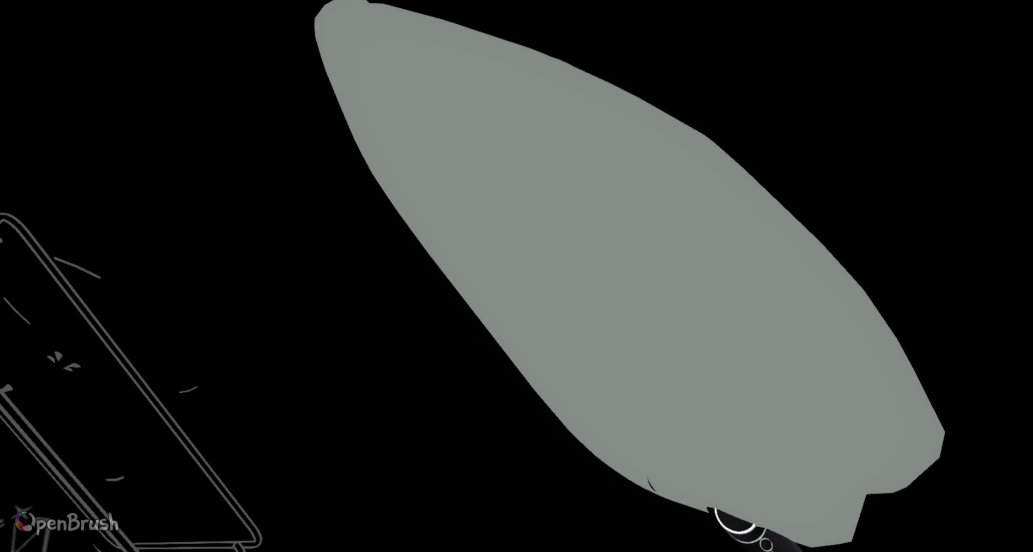
**SOUND 3**

Participant 2: There's a very yellow. Is very big to begin with and then it's sort of like get a little bit less. Like getting a ring.. like you can sort of hear it fading a little but real like actual range is this like really bright orange? Bit brighter or just a small solid. It is, yeah.. Exists within like the sort of yellow shape, so there's like little vibrations with the yellows.

Experimenter: Do you want me to play it again? Or you OK, it's not the most pleasant sound.

Participant 2: I don't mind it too much. It's not the most annoying phone, but it's telephone. It's kind of got that urgency with it.


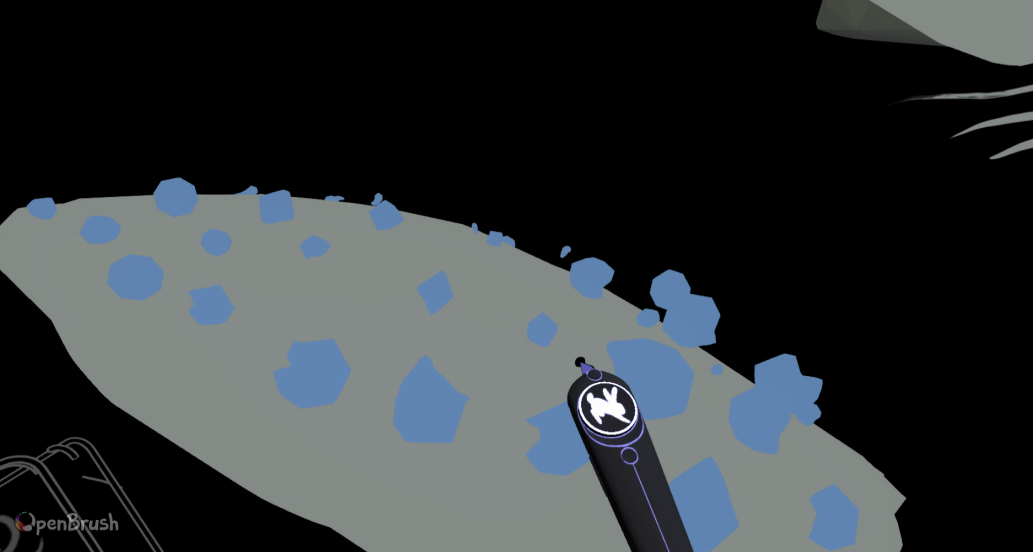
Experimenter: And still goes in this circle, yeah?

Participant 2: It's very round. And so this one is nice, but it's like a a rough. I drew the yellow spiral... It so like the broken up texture makes a lot of sense, but the smoke is sort of Like they're kind of ringing off from it. Like floating away from the shape. And then obviously it starts strong and it gets quieter since doing that sort of like shape where it's like really big to really small and then this orange line is like the mean ring.

Experimenter: And then.

Participant 2: The one that's like really really loud. There should really be 2.

Experimenter: OK.


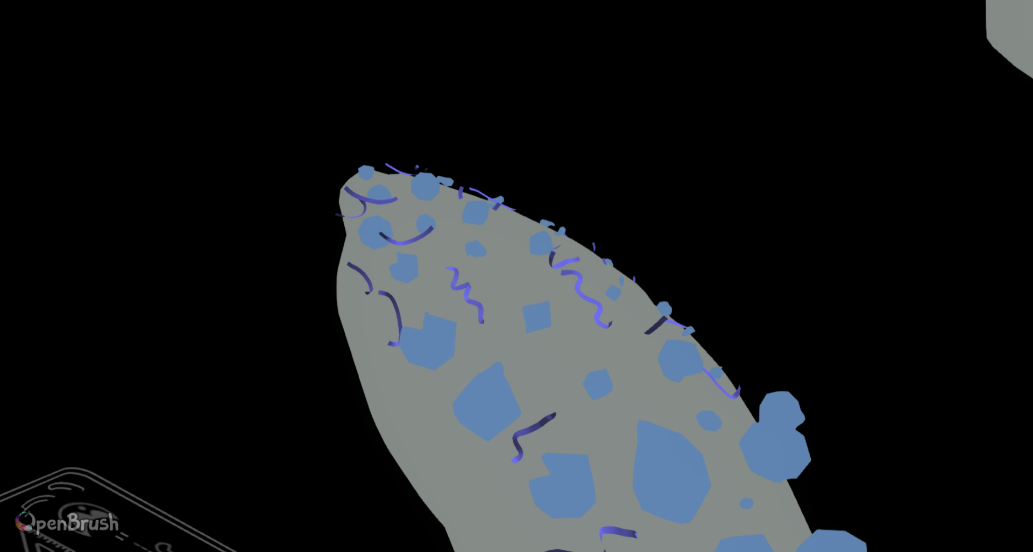
Participant 2: Since it goes … bring bring... so I'll keep the gap between them.

Experimenter: Yeah, I see.

Experimenter: Do you want to add anything else? Or do you want to try different?

Participant 2: And no, I'm happy with this. Yeah, I don't think I have much more to add to.

Experimenter: Teleport away. an you hear? It yeah, is it OK?

**SOUND 4**


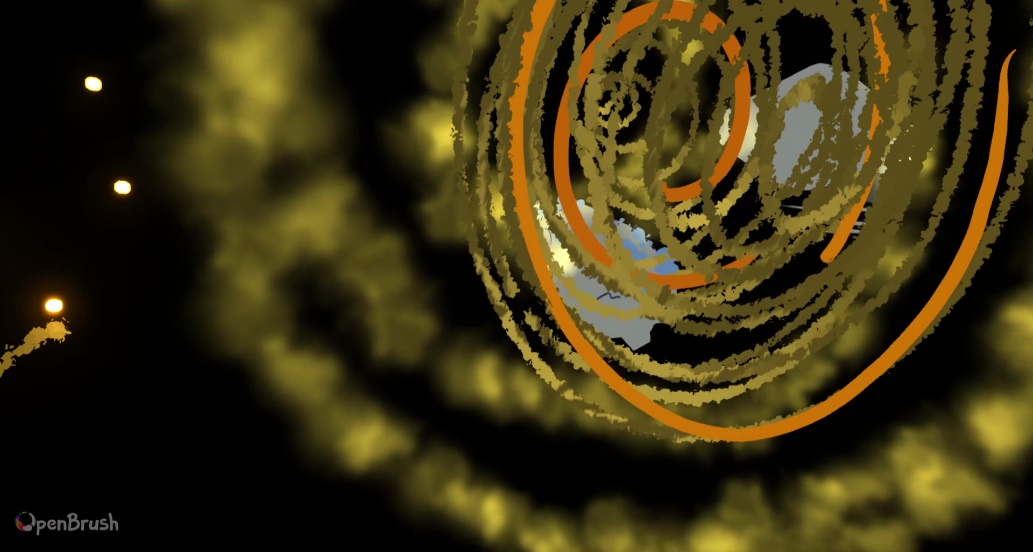
Participant 2: No, it's OK. So I'm just trying to like kind of expanding it with shapes a little bit. So like this like big solid block that's very rigid. It's like got hard edges and almost like a shiny. And and then I was thinking of adding like sort of other colors to the side.. is very green, like a very dark green and and there's like...

Experimenter: OK.

Participant 2: Sort of Blues and things coming through as well. It's very cool. It could even be darker and I think like lind of a more like emerald.

Experimenter: OK, does that block extend further up in space or is it just like a specific different shape?

Participant 2: It would. Extend for yeah just keep going, yeah OK.

Experimenter: Is there movement to the other elements? Or is it quite solid?

Participant 2: It's quite solid. If there is movement, it's not .. It's slow and not very... It's not like rainfall, which is obviously what I'm listening to.. like I don't see it sort of falling that fast. Yeah, it's almost as if like... Yeah, there's like lines of other colors. Could be moving. Very slowly up and down or mostly static.

Experimenter: OK, yeah.. Is it? Do you think it's almost stationary from all the other ones you've done drawn so far?

Participant 2: Like the other ones are floating in space.

Experimenter: Do you have like some movement around them, whereas this one is very much as if it's resting on the ground? And like extends all of the way up. Can walk through it, yeah? And like see the same like material and colors as you can on the outside.

Participant 2: I think it is quite difficult to get this one. The general like hard shape. Is like definitely what I was thinking of.

Experimenter: So well, we got one more.

Participant 2: OK, cool.

**SOUND 5**

**
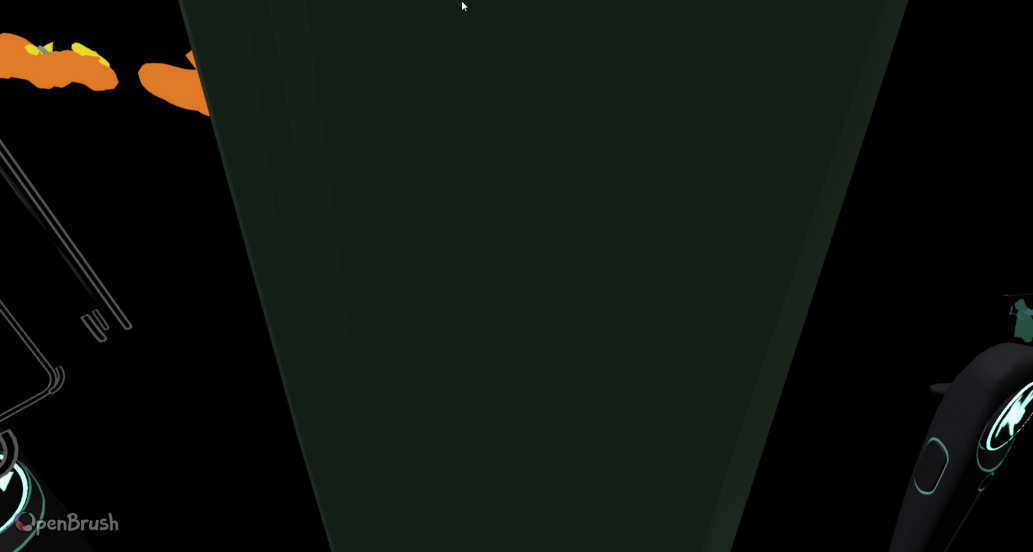
**Experimenter: is that OK? Sounds right. It's very quiet. Do you want me to? Yeah, that'll be good. Is it better? OK, yeah.

Participant 2: It's OK now. It sounds really difficult... Yeah, it's it's really hard to describe.

Experimenter: I mean, just do your best.

Participant 2: And so I think we do this kind of shape but It's like so much smaller, OK? And so it's because it's like very similar and obviously with it being like rain, yeah.. I think this sort of shadiness of these shapes is definitely to do with like water, moisture ..

Experimenter: Is it similar when it rains normally? Would you normally get some similar experience you know when in real life rather than us playing at the computer?


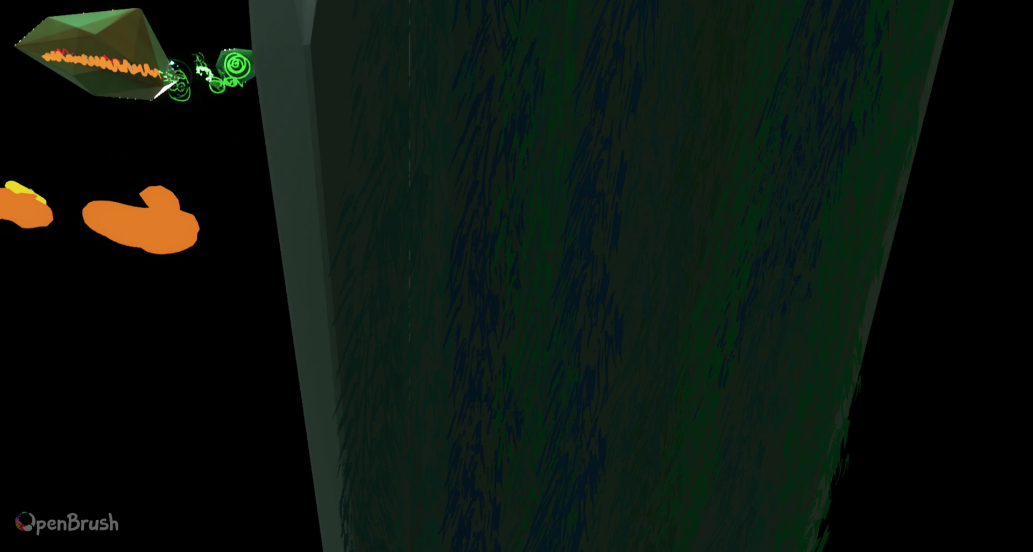
Participant 2: Yeah, so definitely. The colors that I've gone with, like the greens and the blues of like coldness hardness and instinctively what I associate with rain like that and the thing. Of it, the one I've just heard is that it feels very low down and I think so in my body and came up like thinking my feet are going to get wet and like thinking about how that feels... To have to dress for it. Yeah it affects me. It's kind of very much centered down at my feet.

Experimenter: See, it sounds like sounds kind of like dictates of space awareness.

Participant 2: I think so and it's like sometimes and like, yeah, things like that can like... it's so difficult to describe, but sometimes I'll feel a sensation in a part of my body, because it's something that im thinking about. So and yeah, if I was here rain like that, I would suddenly start associating like if I was out there in it.

Experimenter: I see.

Participant 2: Yeah, it's very hard. Like I get ASMR sometimes.

Experimenter: crosses modalities? As you've got visual and audio, but then it could be some other?

Participant 2: Yeah, sure yeah yeah.


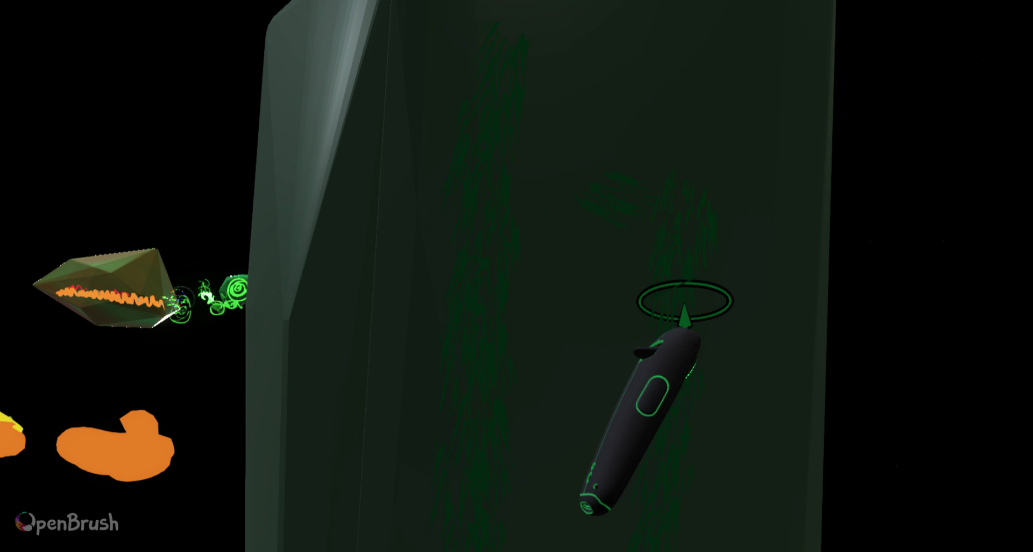
Participant 2: It is really hard to display. Like I saw a dead bird running over and as soon as I saw it, I got this like horrific like it... was like a big lump in my throat and like a sort of like burning sensation in my chest. And it was like a little bit like fright or flight mode... I was imagining that had happened to me. Were they thinking like this has happened to me because you're seeing it made my body parts feel like they're in danger? Looking at something. And then feeling.

Participant 2: kind of adding like little pieces, arranging shapes because they're very slack either. Connect not consistent and like making a mass as well.

Experimenter: Would they be moving again or are they more stationary?

Participant 2: Yeah, they would be moving. They would be sort of in bending around.. all of this sort of locally.

Experimenter: we've been in about 40 minutes so, oh, my.

Participant 2: It's not so much when you're in it, but when you come out, it's confusing.

Experimenter: Yeah, Oh no definitely.

Experimenter: So in terms of any benefits in using VR compared to traditional methods. Do you think?

Participant 2: there's so much more that I can convey and describe about it because I'm able to use like a 3D space rather than trying to draw aomething on a piece of paper. So yeah, a massive advantage.

Experimenter: And do you see, VR is a good method for creating synesthesia experiences as it relates to the previous question.

Participant 2: I think so, yeah.
