# Supplementary material for: Using immersive virtual reality to recreate the synaesthetic experience
Source: Iperception. 2023 Sep 4;14(3):20416695231166305. doi: 10.1177/20416695231166305 (PMC10478570; doi:10.1177/20416695231166305)
Supplement: sj-zip-2-ipe-10.1177_20416695231166305 - Supplemental material for Using immersive virtual reality to recreate the synaesthetic experience [file sj-zip-2-ipe-10.1177_20416695231166305.zip › Transcripts/SN3.docx]

Experimenter: Just draw anything.

Experimenter: Yep, press the trigger again. Have fun.

Participant 3: Is it this one?

Experimenter: All this makes sense?

Participant 3: Yes, yeah. And how it works in 3D

Experimenter: Yeah, if you go more towards the left, there's the one the top right one in the top smoke that seems to be quite popular. With trying to represent perception, yeah.

Participant 3: I can actually see why that would be, yeah.

Experimenter: And remember you. Don't have to just draw in your visual field. You can draw and walk through it. You can if you step forward.

Participant 3: I was thinking that. And then we did, yeah.

Experimenter: We might not have the brushes that might be handy, but it's just sort of trying to get it as closely as possible to what you normally perceive.

Participant 3: Oh, OK, that's why I'm here so.

Participant 3: I feel like I could just touch them. They're very realistic.

Experimenter: I'll play you some music so maybe that will help you to sort of get a sense of the volume.

Participant 3: OK.

Experimenter: It can hopefully helps you to like start. Thinking of what you could draw.

Experimenter: Does something normally come up when you listen to music. Any specific things you get with instruments?

Participant 3: Feels quite l.. And kind of like silky. It's hard to find this, but maybe some of these. Ones you know .. like diamond, they can be shiny.

Experimenter: Try a few different things.

Participant 3: Yeah, maybe this one. Let me try.

Experimenter: Any movement too? It or is it just kind of like a color.

Participant 3: It kind of, uh, beats like a heartbeat.

Participant 3: It's definitely this color. not too. I don't know if it would be silky 'cause it's not shiny and it doesn't look 3D. It's just flat like a block and it may be beats as if it's like moving like this. OK, but not in time to the music.

Experimenter: Interesting and is it moving in space?

Participant 3: See that to be honest and yeah, would still just be 2-dimensional thing, yeah? I don't like you know like skating music because I feel like it's really harsh. And it's like harsh colors and I don't like that image and this kind of music is fine and I feel like this is the kind of music you would hear if you were like on hold on the phone. And normally people hate that, but I'm normally quite like it because I would quite like to see this color. So yeah, just yeah, roughly that and it would just keep going like let's keep going. It was empty and then just refilled. Ah OK, basically yeah well.

SOUND 1

Experimenter: Yeah so. It takes about a minute, but I can play you for as many times as you want. Sometimes people remember it and it's enough. And once or twice. But if you want me to replay it, just let me know and I'll replay it.

Participant 3: Seems really ... like straight away get the differently colored always come first thing. Is maybe like purple like really more white? Definitely being very small. And yeah, probably spikes that way. Yeah, that's what it looks like. If I just like, yeah, just like little noise.

Experimenter: Are they in space or more 2 dimensional and?

Participant 3: Yeah, it definitely looks like this, it's strange to be able to jump.

Experimenter: How do you think you would rate this drawing as a representative of what you see from certified?

Participant 3: I would say it's quite accurate and it before for the only thing it's not accurate because the space behind and also I've accidentally going a little pink line will still be there.

Experimenter: Yeah, yeah. What about this space behind that small?

Participant 3: The space behind it doesn't seem to match up with the signs I associate.

Experimenter: How would you?

Participant 3: That's the issue. Probably just white or maybe a sky..

Experimenter: OK, is there different association for different birds?

Participant 3: Yeah, because bigger birds ones that you don't maybe see in this country. I would say it could be darker like. And I don't really like those colors, so I wonder if it's because I like blue and that's kind of common. It is when it's bigger parts or you know, once you don't normally see, I think, yeah, that's more fitting than coming towards me as opposed to this just being a little bit further back.

Experimenter: Ah, OK, well we can jump.

Participant 3: OK.

SOUND 2

Experimenter: So this time round will have cars passing. It might need to be a little bit louder. But I know. Can you hear it?

Participant 3: Yes

Participant 3: Comes past me. OK so it's moving yeah? But on both sides.

Experimenter: OK, yeah yeah, that's awesome.

Participant 3: Definitely some red.

Experimenter: Would they be moving ideally?

Participant 3: Yeah, they have to go. OK, so it's like 2 separate streams. That's kind of blue.

Experimenter: How representative do you think this particular one is?

Participant 3: I would say that one is three. OK and I'll probably imagine the background to be more white..

Experimenter: But you can change the change the environment.

Participant 3: Yeah, I'm gonna do that.

Experimenter: Is that better?

Participant 3: Going to keep it like that stand OK even. The colors little more representative.

Experimenter: Would you rate it higher?

Participant 3: definitely higher. I'm gonna say 5 to be honest.

Experimener: OK.

SOUND 3

Experimenter: We'll try next one, yes. So this will be a phone ringing.

Participant 3: This is going to sound strange. But I feel like I can taste this. So it's definitely a color. And yeah... but it tastes like, you know, you get those... Little tubs of peers. Yeah huh? It's like the texture. If you were to take a serated knife and to cut them. So it's kind of like shiny.

Experimenter: And how would they move in, do they move in space?

Participant 3: Seem to be at the side of both of my ears. Not only that... I wouldn't put in front of me. I would envision it to be beside.

Experimenter: Accurate, do you hink of this one is?

Participant 3: Yeah, I can still taste. It, but honestly I can't draw that in real life, so...

Experimenter: Do you want to spend more time? Doing this or do you want to try another one?

Participant 3: No, I think this is fine I. Know it's simple, but I feel like this is what I would feel.

Experimenter: So we'll move to. The next one. So this will be rain OK. So let me know if it's too quiet.

Participant 3: Oh, OK. Feeling this is so white. OK, for this one I feel like it's. There's not as much.... I don't know how to sum up this picture.

Experimenter: OK, do your best you can describe it if you can't find the exact brush.

Participant 3: And I need to change the color. And then we're behind it, it feels like a sound that I wouldn't really notice like. It's because it's every day you get a lot. Especially in Scotland. Yeah, I don't really feel that much for that one. The colors are so light, which again is because it's like it's not like a striking color. I feel like that one is difficult. It's not really one that I don't have a clear vision. It's just like a sort of a sense of it being there, if that makes sense.

Experimenter: Yeah, yeah no, that makes sense. So obviously you can't really rate it, but I suppose it's not something you could draw as it so actually.

Participant 3: Yeah, struggling..

SOUND 4

Participant 3: That it reminds me of taste of sweets. Like you can. It's like the sprinkles that you would get on a cupcake. But I feel like I just feel texture. That happens a lot, that's it's crosses over. It's not just visual. It's just weird to just describe this.

Experimenter: OK.

SOUND 5 – technical difficulties

Experimenter: So do you think VR is good for recreating your visual experiences?

Participant 3: Yeah, definitely. It's mostly difficult to sometimes describe it, if I can't draw it.

Experimenter: Oh, OK. Then the last other question is just any other comments in general?

Participant 3: OK. I don't think so, no. I just think it's yeah I think it's good and I think that I would imagine that most people would. I would assume they would agree because it's more realistic.. You need to just draw what I can see. Pen and paper are too small and doesn't get the same accurate representation so...
