# Supplementary material for: Using immersive virtual reality to recreate the synaesthetic experience
Source: Iperception. 2023 Sep 4;14(3):20416695231166305. doi: 10.1177/20416695231166305 (PMC10478570; doi:10.1177/20416695231166305)
Supplement: sj-zip-1-ipe-10.1177_20416695231166305 - Supplemental material for Using immersive virtual reality to recreate the synaesthetic experience [file sj-zip-1-ipe-10.1177_20416695231166305.zip › Results.zip!/Brushes used in Tilt Brush.docx]

The following table details which brushes were used for the creation of each synaesthetic illustration.

| Participant | Stimuli | Brush Used |
| --- | --- | --- |
| 1 | 7 | Smoke |
| 1 | June | Marker, Smoke |
| 1 | All days and months except June | Marker |
| 2 | Researcher’s voice | Smoke |
| 2 | Birdsong | Dr.Wigglez, Marker |
| 2 | Cars: Full volume | Streamers, Spikes |
| 2 | Cars: Half volume | Streamers, Bubbles |
| 2 | White noise | Highlighter, Soft Highlighter |
| 2 | Phone ringing | Smoke |
| 3 | Piano note | Icing, Unlit Hull |
| 3 | Birdsong | Electricity |
| 3 | Rain | Matte hull, Smoke |
| 3 | Cars | Matte hull, Smoke, Pinched Marker |
| 3 | White noise | Electricity |
