# Supplementary material for: Using immersive virtual reality to recreate the synaesthetic experience
Source: Iperception. 2023 Sep 4;14(3):20416695231166305. doi: 10.1177/20416695231166305 (PMC10478570; doi:10.1177/20416695231166305)
Supplement: sj-zip-1-ipe-10.1177_20416695231166305 - Supplemental material for Using immersive virtual reality to recreate the synaesthetic experience [file sj-zip-1-ipe-10.1177_20416695231166305.zip › Results.zip!/Participant 1 Results (session 1).docx]

**Participant 1 Results**

| A | Bright red. |
| --- | --- |
| Bb | Uppercase - Green  Lowercase - brown, described as a pale, green-tinted brown. He thinks this may be due to the similarity of the shapes of b and d, perhaps due to Mears-Irlen syndrome. |
| C | Lilac |
| D  (d) | D is the texture of dark brown hair – if you took a close-up photo of dark brown hair and cut the letter D out, that is how he sees it. He thinks this is due to his association of the letter D with the word dad, as it is his dad’s hair colour.  However, in the context of the word day, lower case d is blue. |
| E | Yellow |
| F | Blue |
| Gg | Uppercase G is green and lowercase is lilac. The number 7 flickers between these two colours as well as grey, and g is the 7^th^ letter of the alphabet. |
| H | Dark green |
| Ii | Uppercase I is black, lowercase blue |
| Jj  (J) | Rusty orange, with uppercase J being brighter and lowercase more muted. In the name Jay, the J is blue |
| Kk  (K) | Brown, uppercase darker than lower. Can sometimes be blue. |
| Ll | Uppercase navy blue, lowercase brighter blue |
| Mm | Lilac, possibly due to the association with the word mum, & his mum wore lilac a lot when he was a child. |
| Nn | Uppercase green, lowercase pale lilac |
| O | Black |
| Pp | Pinkish-lilac – lowercase is more lilac than pink |
| Qq | Uppercase Q is aubergine, but a bluish aubergine, possibly due to the O shape in Q. Lowercase is more purple |
| Rr | Red – again R is darker, again possibly due to the rounded shape of the letter R being reminiscent of the letter O |
| S | Matte darkish yellow. Lower case s is very slightly duller but almost exactly the same |
| Tt | Dark green. Lower case t is almost imperceptibly duller |
| Uu | Purple – lowercase is a lighter & brighter purple |
| Vv | Uppercase – darkish magenta  Lowercase – lilac |
| Ww | Both purply-blue, uppercase more purple & lowercase more blue |
| Xx | Uppercase -navy, but can also be black or bright blue. Lowercase – bright blue  Note: x as you would draw it in algebra (more rounded) is even brighter and “friendlier” |
| Yy | Yellow, uppercase is more matte & darker |
| Zz | Same as X.  Uppercase -navy, but can also be black or bright blue. Lowercase – bright blue |

| 0 | Black |
| --- | --- |
| 1 1 | 1 is black when it is followed by a 0, but on its own it is blue |
| 2 | Yellow |
| 3 | Green |
| 4 | Blue |
| 5 | Red |
| 6 | Greyed out pink |
| 777 | 7 is the most interesting number – while the other numbers (and letters) are static colours, 7 is iridescent, flickering between lilac, green and grey. Difficult to capture; also related to gender and personality as other numbers have binary genders but 7 is non-binary, and complex, introverted, shy. The colour is almost like when you see colours in an oil spill, with grey being the base colour and green and lilac swirled in |
| 8 | Pale light pink |
| 9 | Dark aubergine. Dark because of influence of the circular shape (looks like an O) |

| Monday | Because O is so early in the word it darkens it to a very dark purple.  Attention: inverse logarithmic |
| --- | --- |
| Tuesday | Dark green because of U, still aware e is bright orange.  Attention generally flowing downhill with a small peak/blip/uptick at e |
| Wednesday  Wednesday | Overall colour is lilac but e shines through, almost like the orange is superimposed on the lilac so it’s there but not interfering with the lilac.  Shape of attention: like a rollercoaster up and down |
| Thursday  Thursday | Very dark, almost black – T and H individually are dark but Th together is even darker. U is notable for its purpleness rather than its darkness, whereas in other words, u is notable for being dark and darkening the whole word.  Shape: plateau at start of Th, then down with a small peak at U (I have a sketch which captures this better) |
| Friday | Red because of r and a, and comes at you all at once. F and I are blue so it’s odd that Friday is so red – it takes effort to see these blue colours, he has to think of it no longer as Friday and more as a collection of letters.  Shape: like a flash, all at once, maybe because of transition between weekday and weekend.  Maybe association with word fire in German |
| Saturday  Saturday | Overall yellow. The As are an understated red in a way that it is usually difficult for red to be.  Attention – a lazy swooping from start to finish, with upticks at the As, finishing at same intensity as it started. This is a lower intensity than the weekdays – 4 or 5 rather than 8 or 9 (if plotted from 1-10 on x axis) |
| Sunday  Sunday | Darker, richer yellow than Saturday. U is a slight attention blip as its purple – almost like a reminder of upcoming Monday. Intensity of around 6/7. |

| January  January | R and N – he doesn’t pay attention to these, so the colours are like the letters have an absence of their own colour so take on the J.  Eclectic, swinging |
| --- | --- |
| February  February | Overall a watery blue. More homogenous than January, but e and ua are points of interest. Ua are blended, fading together like they were one letter |
| March  March | Dominant colour given to A and R  M & C still get a look in  Bleeding of AR is very strong, even though he is aware that m is a paler colour  Comes as a flash of red but on a secondary level he is aware M and C are paler |
| April  April | Orangey yellow, similar to S (but slightly more orange then yellow) – even though there are no orange or yellow letters. |
| May  May | Pink M is dominant and permeates the whole word. Slight peak of interest/awareness that a is red |
| June | Both purple and orange at the same time. Complex colour – like a sunset gradient – purple and orange mottling together. He is aware which letters contribute which colour but the effect is very much a mingling of both colours. Purple very slightly more dominant. A bit like when orange shines through purple clouds |
| July | Matte dark purple not black. More purple because of the U. |
| August | While second half is more purple, orange A is very dominant. Overall impression comes from orange A and purple u – sees these almost as one vowel |
| September | Overall yellow from S so Es don’t stand out. Very flat attentionally with tiny blips for Es. Focus is slightly more on the letter S but the yellow colour leeches onto the whole word fairly equally |
| October | Overall brown but darker at the beginning. Gradient dark-light. O is fairly “shocking”/attention-grabbing – 8 or 9 |
| November  November | Dark blue – gradient down with sight peaks at two orange Es.  Dark because of O  Both Es only noticeable because first E appears early enough to then highlight both |
| December  December | Warmer, more muted brown than D on its own due to the Es |
