# Supplementary material for: Using immersive virtual reality to recreate the synaesthetic experience
Source: Iperception. 2023 Sep 4;14(3):20416695231166305. doi: 10.1177/20416695231166305 (PMC10478570; doi:10.1177/20416695231166305)
Supplement: sj-zip-1-ipe-10.1177_20416695231166305 - Supplemental material for Using immersive virtual reality to recreate the synaesthetic experience [file sj-zip-1-ipe-10.1177_20416695231166305.zip › Results.zip!/Participant 1 Results (session 2).docx]

**SESSION 2 – Feedback on Tiltbrush recreations**

7 - most accurate depiction he has seen so far

- The indistinct nature of the number 7 is its defining feature; therefore, the distinctness of the VR recreation is what makes our depiction inaccurate
- our depiction is like if you get corrective lenses and people don’t look quite the same but you know it is them
- Tilt Brush definitely brings something better than mediums like Paint
- overlay of colours captures what he was trying to describe but had difficulty in describing
- like he’s “seeing it naked”
- 4.5/5 - the thing that makes it inaccurate is that he’s physically seeing it

Days – Saturday and Wednesday are very good

- all good schematics but not literal representations
- shapes are more about how much focus he gives them, rather than how he literally sees them
- graph could be better used as an indicator of the boldness of the letters
- more like latter half of Monday fades out of perception - so maybe background darker at end
- he doesn’t feel like VR brings anything new here
- Monday: ⅖ - slanting is distracting
- Tue: ⅖ - ue should be like e on wed
- Wed: 3.5 - generally this is good
- Thurs: 3/5 - impossible to show u as purple as well as yellow - hard to explain and hard to recreate
- Fri: ⅖ - should be more red
- Sat: 3.5/5 - yellow around a would be better
- Sun: ⅗ - impossible to show u as purple as well as yellow

Months - overall better than days because months are more “topsy turvy”

- shapes match personality of months more - again not literal shape, but it is a good match and retains the feeling well
- Jan - ⅘ - if no line but letters were still in these positions it would be better
- Feb - 3.5/5 - too much up and down
- March - ⅘ - M and c should be softer
- April - 3.5/5 - instead of mottling, it’s very one thing then the other - he never sees it like this as a whole, rather if he thinks specifically of the end, it is very blue. Like if you have a warm tone sound then a violin at the end. Not sure if yellow underneath might help
- May - ⅘ - soft line underneath helps, can’t take it away and get same impression
- June - ⅘ - base purple is very accurate. Orange not how he imagines it. purple is a 4.5 and orange a 3, overall impression is a 4. Good that orange does not interfere with purple. The smoky effect captures idea of sunset coming through clouds well
- July - 2.5 - colour too grey because of screen
- August - ⅗ - line helps - A should be deeper colour
- Sept - ⅗ - September is simple - colour is too bright - should be more “cat sick”
- October - 3.5/5 - generally good
- November 3.5/5 - “Nov” could be darker - lines okay
- December - ⅘ - generally good

**Does participant see any benefits of using VR?**

Generally, doesn’t bring benefits for his grapheme-colour synaesthesia except for number 7, and here it captures it better than any other medium.

**Does participant see potential in using VR as a method of recreating synaesthesia?**

- Yes: dark background would be better
- Also using headset personally would be better - would feel more like being alone in your head

**Overall thoughts**

Biggest issues with Tilt Brush - translating the idea of what feels important, as impossible to separate grapheme-colour and ordinal-linguistic personification
