# Supplementary material for: Using immersive virtual reality to recreate the synaesthetic experience
Source: Iperception. 2023 Sep 4;14(3):20416695231166305. doi: 10.1177/20416695231166305 (PMC10478570; doi:10.1177/20416695231166305)
Supplement: sj-zip-1-ipe-10.1177_20416695231166305 - Supplemental material for Using immersive virtual reality to recreate the synaesthetic experience [file sj-zip-1-ipe-10.1177_20416695231166305.zip › Results.zip!/Participant 2 Results (session 1).docx]

**Participant 2 Results**

*Everyday Sounds:*

5 everyday sounds were played, but the participant couldn’t hear the last one so only 4 are reported below. All were played at full volume first, then repeated at half volume.

| Birdsong | Initially, this was seen as an orangey colour “jiggling” back and forth in the bottom right corner. This then jumped across to middle left and became a static peach colour, which was octagonal and twisted as the chirps changed. When played at half volume, there was no experience in the bottom right. |
| --- | --- |
| Cars Passing | This elicited a pink waterfall starting at ¾ way up on the left-hand side. Just below halfway, purple shards were seen. At half volume, the shards were seen as bubbles instead. Where the bubbles began, the waterfall began falling straight rather than continuing on its path. |
| White Noise | This sound elicited white waves similar to that of a heart rate monitor. The waves were very close together and left a trace of around 10% as they moved from left to right across her visual field.  When played twice with a gap in between, the wave went back to the start after the short gap. When played on a loop, the wave got stuck at the end after about 13-14s. No difference was observed at half volume. |
| Phone Ringing | A teal aura was experienced all over her visual field. This was the same at half volume. This may be because a phone ringing is associated with voices. |

*Musical Notes:*

7 notes were played on the piano and 7 on the guitar. For the piano notes, all were seen as

A pinprick of red at eye level on right-hand side. “E” was a larger pinprick. There was more variation in the guitar notes. “A” elicited the same red pinprick as the piano, but at nose level. “B” and “C” elicited a green pinprick at nose level. “G” elicited a brown pinprick in the bottom left. No synaesthetic experience was observed for notes “D”, “E”, or “F”.

*Voices:*

| Researcher’s voice | Like a vignette around a photograph. The colours were bottle greens and lemon yellows. Any time I said “em” there was a flash of lime green in the top right-hand corner. |
| --- | --- |
| Female voice 1, “hello” | On the “o” of hello a bright yellow colour jumped up from around feet like jack in the box |
| Female voice 2, “hello” | No synaesthetic concurrent |
| Male voice 1, “hello” | No synaesthetic concurrent |
| Male voice 2, “hello” | Similar to female voice 1, except mustard yellow, and on left-hand side rather than centre |
| Male voice 3, “hello” | No synaesthetic experience |
| Queen Elizabeth, “good morning” | Felt like she was trying to look through a black screen |
| Barack Obama, “hello everybody” | Bouncing ball, random movement - lighter teal colour, dissolves into aura |
