# Supplementary material for: Using immersive virtual reality to recreate the synaesthetic experience
Source: Iperception. 2023 Sep 4;14(3):20416695231166305. doi: 10.1177/20416695231166305 (PMC10478570; doi:10.1177/20416695231166305)
Supplement: sj-zip-1-ipe-10.1177_20416695231166305 - Supplemental material for Using immersive virtual reality to recreate the synaesthetic experience [file sj-zip-1-ipe-10.1177_20416695231166305.zip › Results.zip!/Participant 2 Results (session 2).docx]

**SESSION 2**

| My voice | 2/5  Colours are relatively accurate, but should be a lot more gentle. The frame was drawn very definitively, but really there is no end to the image, it fades out into the periphery. |
| --- | --- |
| Birdsong | Bottom right (jiggly paintbrush): 5/5 Although the shape isn’t quite accurate and the colours a little too solid, the movement and rough edges are very accurate. Participant expressed surprise at how well this captured her experience.  Middle left (peach octagon): 0/5. No movement from right to left, and overall, very inaccurate depiction. |
| Cars: full volume and half | 4/5 for both depictions. The colour is not quite right, but the movement is good (the way it falls in different places is also a good representation of how speech patterns can be, particularly for people with lyrical voices) |
| White noise | 3/5  The movement and fading out are very important aspects of this sound, and this was not captured. Also, the waves needed to be more angular. |
| Phone | 5/5  Good because of the transparency. The subtle motion is good but it should be more of a pulsing with each ring. |

**Does participant see any benefits of using VR?**

Some benefits - appears capable of capturing certain elements that other mediums can’t (e.g., jiggly paintbrush for birdsong)

**Does participant see potential in using VR as a method of recreating synaesthesia?**

Yes potentially - difficult to tell due to VR-by-proxy method, but participant was surprised at how much of her experience was accurately recreated

**Overall thoughts**

Overall, the participant expressed surprise at how much of her experience was accurately recreated in VR. However, other aspects were impossible to capture, particularly with regards to certain types of motion. She also noted that her synaesthesia is very difficult to explain, so there would always be a loss in accuracy when another person attempts to reproduce it.
