# Supplementary material for: Using immersive virtual reality to recreate the synaesthetic experience
Source: Iperception. 2023 Sep 4;14(3):20416695231166305. doi: 10.1177/20416695231166305 (PMC10478570; doi:10.1177/20416695231166305)
Supplement: sj-zip-1-ipe-10.1177_20416695231166305 - Supplemental material for Using immersive virtual reality to recreate the synaesthetic experience [file sj-zip-1-ipe-10.1177_20416695231166305.zip › Results.zip!/Participant 3 Results (session 1).docx]

**Participant 3 Results**

*Everyday sounds:*

5 everyday sounds were played.

| Birdsong | This elicited sparkly speckles in the top centre. These were a blue and creamy colour, tiny and not particularly vivid |
| --- | --- |
| Rain | A misty metallic grey mass in the middle, with little bumps protruding outwards. These were not static, they moved slightly (the bumps) |
| Phone Ringing | This was seen as little rose-gold beads in a chain moving towards him. The beads left a white shadow behind them, still taking the form of beads. They come from the middle and get wider as each ring moves towards him. |
| Cars Passing | Similar to the rain one, but less grey and more white, again a mass in the middle, as car passes getting bigger and expanding, then getting smaller. Yellow strip at top. White ball most dominant |
| White Noise | Central - thin blade coming towards him. If he turned round and saw it from the side it would be like TV interference. Mostly white, slightly yellow, like a blade, metallic. |

*Musical Notes: Piano*

All piano notes take the same form. They move upwards or downwards on the y axis depending on pitch. The shape is a 3-dimensional, smooth edged triangle - “creamy pinky orangy colour, sort of yellowish as well, with a slightly darker edge” Texture: like cheap plastic.

*Musical Notes: Guitar*

Again, for all guitar sounds the form is the same and only the positioning changes depending on pitch. Another triangle, concave edges, dark green/khaki colour, dark tone.

*Music: Short Melodies*

5 short melodies, of varying lengths (3-13s), were played

| 3s piano | Piano melody so same piano shape as before, triangle just moves with melody up and down - up down up with melody |
| --- | --- |
| 5s piano | Different tone piano so edges softer and more blurred - more pinky colour - more blurring in movement - not sharp jumps so more of a hazy blur |
| 7s piano | Back to initial shape. Base note pulsing at bottom - darker and bigger - copies of shape stacked on top of each other & moving as tune moves - bouncing off each other. Always same shape just lower is always darker and bigger |
| 11s piano | Chords darker at bottom - not as low as last one. Cluster of notes move on top move as chords move - higher notes at top moving about on top as one moving around in another register |
| 13s piano | Bad quality so experience not that vivid. Whole thing is hazy - not blurry but like vid in 140p. Hazy and undefined. Electric piano so purply pink (not like light pink as before) almost like rubber balls - quite low down - green triangles round side - light pink triangles around top (these are alternating positions at top) |

*Voices: One word*

For words and human voices, participant 3’s two forms of synaesthesia (grapheme-colour and auditory-visual) end up competing with each other. If he focuses purely on the sound, he has a different experience from if he focuses purely on the word. Usually, both end up mixing. The shape of all words and phrases are the outline of a mouth shape saying the words. Shape is the dominant feature rather than colour.

The word “hello” was said by 10 different speakers, 5 females and 5 males. All had similar accents. All words beginning with the letter h are a light brown, so all words were first experienced as the colour reported below, then with the competing experience of the light brown. Depending on if he focussed on sound or meaning either the colour below or brown could be seen, or both.

| F1 | Yellow |
| --- | --- |
| F2 | Silvery colour, coarse and glistening. Further down on y axis than previous |
| F3 | Very quick - so less movement - more like an orangy pulse of colour |
| F4 | Higher pitch so higher up, thinner grey - last was like a ball this one is more like an oval - again a quick flash of something because so quick, but still interference from brown hello because he still could understand the meaning of the word |
| F5 | A lot softer so not defined in edges, greyish colour - again too quick for colours to manifest - softer more like soft toy - again mouth shape but rounder shape |
| M1 | thin dark blue - nearer bottom because lower - little bit of a squareness because it sounded electronic - texture: like touching something electric |
| M2 | Higher pitched and more rounded - because of intonation moves up then down  Indistinct grey colour (with sound more of shape and texture) - but immediately overridden by brownish |
| M3 | Much higher pitched - yellowish creamy - again intonation movement  All Scottish accents so shapes are all v similar |
| M4 | Very thin - no movement - monotonous - light blueish grey - square, static |
| M5 | Lower, gravelly, again monotonous - deep grey low on y axis - again ball moving like mouth but more bumpy |

Next tried phrases but found this was unsuitable/ too complicated (as concurrent experiences were a mouth shape continuously moving).
