# Supplementary material for: Using immersive virtual reality to recreate the synaesthetic experience
Source: Iperception. 2023 Sep 4;14(3):20416695231166305. doi: 10.1177/20416695231166305 (PMC10478570; doi:10.1177/20416695231166305)
Supplement: sj-zip-1-ipe-10.1177_20416695231166305 - Supplemental material for Using immersive virtual reality to recreate the synaesthetic experience [file sj-zip-1-ipe-10.1177_20416695231166305.zip › Results.zip!/Participant 3 Results (session 2).docx]

**SESSION 2**

Piano note - 2.5/5 - colour is good, should be more rounded. Difficult to say if texture is accurate. VR looks promising as a method of exploring synaesthesia though

Birdsong - 5/5 - very close to how he experiences it, especially from a distance. Less so up close as he doesn’t see it so close up

Rain - ⅗ - not quite a ball, feels more like he’s in amongst it - should be much closer, almost among it

Cars Passing - ⅗ - again too spherical, also too smooth. Should be more like a cloud or a chunk of mist in a room. Colour is good and the effect of lighter and darker is good, yellow strip at top is accurate - only shape inaccurate

White noise - ⅘ - should be longer (like paper) but static texture is good, and point is good

**Does participant see any benefits of using VR?**

Likes that you can move around VR environment, 3D element is very good, interactivity is good

**Does participant see potential in using VR as a method of recreating synaesthesia?**

Yes - looks promising, would like to try it out himself
