# Supplementary material for: Using immersive virtual reality to recreate the synaesthetic experience
Source: Iperception. 2023 Sep 4;14(3):20416695231166305. doi: 10.1177/20416695231166305 (PMC10478570; doi:10.1177/20416695231166305)
Supplement: sj-zip-1-ipe-10.1177_20416695231166305 - Supplemental material for Using immersive virtual reality to recreate the synaesthetic experience [file sj-zip-1-ipe-10.1177_20416695231166305.zip › Results.zip!/Participant 4 Results (session 1).docx]

**Participant 4 Results**

*Everyday Sounds:*

5 everyday sounds were played. Results are shown in the table below.

| Rain | Bluey green - single rough line, like side view of carpet |
| --- | --- |
| Birdsong | Stabs of very bright yellow - acidic yellow - unpleasant |
| Phone Ringing | Dull red - not quite burgundy - little floating shapes like a porcupine (wobbly circle with lots of little points) - same shape repeated but moving, fluid, not identical |
| Cars Passing | Bottom quarter of visual field, slightly uneven solid colour - dull grey with a hint of green & blue, indistinct bands of earthy yellow |
| White noise | Very pale bluey grey, covers entire vision like a wall - transparent - like a semi-opaque film |

*Voices:*

10 voices were presented saying the word “hello”. 5 were female, and 5 were male. Then, a further ten, 5 female/5male, were presented saying “colour”. All synaesthetic associations were experienced on the left-hand side of the visual field.

| F1 “hello” | Yellow ochre - earthy yellow - linked to length of sound so short - fuzzy edged rectangle floating in mind’s eye about three quarters of way up |
| --- | --- |
| F2 “hello” | Same colour but a warmer version - same place and shape - bending up the end |
| F3 “hello” | Fuzzy triangle on its edge like play button - pale blue - lower down (about halfway up) |
| F4 “hello” | Similar colour but slight bit of purple - bluey -violet - slightly lower down, same shape |
| F5 “hello” | Back into higher field like first ones, back to an ochre with a bit of blue - like pale khaki.  Khaki at one end, goes down to ochre (remove blue as you go down)  Cross between rectangle and triangle (made sketch) |
| M1 “hello” | Mid brown - not reddish-brown, rather brown with a bit of blue - dull  Fuzzy rectangle in lower half of visual field |
| M2 “hello” | Same base of brown, got a bit of red in it - top graduates to red - less blended  Same shape and place as M1 |
| M3 “hello” | Longer thinner rectangle going up at end - ochre (earthy sandy) - lighter tone towards the end - a bit higher than halfway (⅗) |
| M4 “hello” | Reddy earth, mix between ochre, bit of brown, bit of red, quite pale though - mixed with white, truncated triangle/rectangle again (like F5) - about halfway up |
| M5 “hello” | Soft blue with a slight green to it - take a bit of ochre and mix with blue  ¾ way down - distorted rectangle again, shortish (mid length) |

| F1 “colours” | Like a cone on its side - pale blue - less fuzzy - ¾ way up |
| --- | --- |
| F2 “colours” | Same cone shape - slightly fuzzy this time - same base blue but going a bit green - earthier version - slightly lower down than previous |
| F3 “colours” | An earthy blue/ dirty greenish, ¾ way up, not fuzzy  Quite short and percussive (made sketch) - two tier Christmas tree on side |
| F4 “colours” | Same shape - more fuzzy, same position - back up to earthy colour again - more brownish ochre w a hint of purple |
| F5 “colours” | Christmas tree again but elongated - slightly higher up, bit fuzzy, plain ochre again |
| M1 “colours” | Christmas tree shape - edges softened - ¾ down - darkish brown with hint of purple |
| M2 “colours” | Longer triangle on side, halfway up, not fuzzy, at beginning of triangle quite percussive - ochre/brown/purple/ but lighter in tint |
| M3 “colours” | Softer Christmas tree shape, longish - almost twice as long, fuzzy, brown w purple, ¾ down |
| M4 “colours” | Sideways triangle, pointed end extends a bit (pull end of triangle out) fades out, quite indistinct  Halfway up, not very fuzzy but a bit, blue with a bit of ochre → pale earthy green |
| M5 “colours” | Sideways Christmas tree (normal one) - slight pulling away at apex, paler brown w purple, lighter tint  Down in bottom half - pretty low |

Next, two phrases were presented. Again, 5 female & 5 male voices were played for each phrase. The two phrases were “some have accepted it as a miracle without physical explanation” and “I urge you to submit your essay by the end of the week.”

| F1 phrase 1 | Same colouring and positions as equivalent of single words  Band going across with random points probably following intonation of voice  Ochre colour ¾ way up |
| --- | --- |
| F2 | Same as F1 but slightly darker ochre band |
| F3 | Paler yellower ochre, quite high up, a bit like saw edge (again following consonants) - words more distinct so saw tooth rather than fuzzy |
| F4 | Slightly darker, similar saw tooth shape  Lower down– just higher than halfway  Darker ochre with hint of blue so greenier earthier ochre |
| F5 | Band but this time more of a bluey ochre - khaki with hint of purple  Lower half  Wavier band |
| M1 | Brown with hint of purple  Lower half  More wavy, slightly fuzzy |
| M2 | Woody - slightly purply tint of bleached tree  Wavy soft fuzzy band  Lower half |
| M3 | Back into top half  Wavy band, bit fuzzy, quite a dark ochre with bit of brown, hint of purple |
| M4 | Slightly orangey ochre  Softer sawtooth band  ¾ way up |
| M5 | Pale purple with tiny bit of earthy ochre  Band is wider than others, quite fuzzy, waves are flatter  Quite low down, almost at bottom |

| F1 phrase 2 | Narrow band, quite high up, ochre again but less earthy - like a pale yellow  Soft wavy band not fuzzy, flatter and narrower |
| --- | --- |
| F2 | Higher than last one  Same colours but lighter  Not fuzzy, waves slightly sharper |
| F3 | Earthy pale khaki  Halfway up  Slightly fuzzy softer band |
| F4 | Very similar to previous  Slightly lower, softer waves, colour slightly browner – brownish-ochre |
| F5 | Back up to slightly yellow ochre up top, quite high up, narrow flatter band, points sharper - less prominent but sharper |
| M1 | About middle, mid-brown with tiny hint of purple - wider than last one (back to normal medium band)  Slightly flatter - undulating but flattish |
| M2 | Brown with blue and grey - slightly paler  Quite low down - in lower quarter  Wide band - relatively flat with odd saw tooth |
| M3 | Dark brownish ochre, hint of green - like a khaki again (brown-khaki)  Quite wide band, saw-tooth, ¾ way down |
| M4 | ¾ way up  Narrowish band with fairly distinct sawtooth  Slightly greenish-ochre |
| M5 | Purply blue with little bit of brown - slightly pale  Wide band down at bottom, average amount of sawtooth |

*Music*

Finally, 4 short piano melodies of varying lengths were played, as participant 4 stated “I have chromesthesia but the boundaries between perceived colours have overlaps and relate to timbres and tones rather than individual notes.” For music, bass sounds trigger dark colours such as dark blue. Moving through the colour wheel, strings are orangey, extremely high sounds like a bright high violin would be white.

| 3s piano | Pale cobalt blue  Series of triangles on side which would match melody  Longer triangles all joined together (like long, tall Christmas tree)  Last triangle pulled and faded out (paler than others)  No fuzziness. Experienced ⅘ way up  Each triangle only slightly higher or than other depending on pitch (but subtly, not jumping) |
| --- | --- |
| 5s piano | Blues again - warmer blue with hint of magenta - slightly transparent  Series of little triangles but shorter, again matching melody and again last one pulled out (but same colour as others this time)  Quite narrow triangles, ¾ way up  Again, very slight ranging in position, overlapping each other |
| 7s piano | Quite high up  Darkish blue with hint of purple, darker than previous, pleasant strong lilac  Triangles again - quite distinct but no overlap, more opaque - again upper or lower slightly with notes  Chords - 7 or 8 times - at top and bottom of those triangles there are similar triangles but lighter at top and darker at bottom  Slight fuzziness to the whole thing - triangles distinct but general impression is almost out of focus  Quite warm overall |
| 11s piano | More towards yellow  Series of very distinct only just touching triangles (smallish, band is narrow) which do meander up and down depending on melody - much more movement than previous melodies  Very distinct, not fuzzy  More complex chords this time (4) so: more than 3 triangles, more like ⅘ (can’t pick out exactly) - lighter and darker again, below ones have a bit of orange tint  Warm again |

As all shapes experienced are 2D, participant doesn’t see point of Tiltbrush over e.g., photoshop.
