# Supplementary material for: Using immersive virtual reality to recreate the synaesthetic experience
Source: Iperception. 2023 Sep 4;14(3):20416695231166305. doi: 10.1177/20416695231166305 (PMC10478570; doi:10.1177/20416695231166305)
Supplement: sj-zip-1-ipe-10.1177_20416695231166305 - Supplemental material for Using immersive virtual reality to recreate the synaesthetic experience [file sj-zip-1-ipe-10.1177_20416695231166305.zip › Results.zip!/Participant 5 Results (session 1).docx]

**Participant 5 Results**

A

B

C: yellow

D

E

F

G

H

I: white

J: pale yellow

K

L

M

N

O: white

P

Q

R

S

T

U

V

W

X

Y

Z

1: yellow

2

3

4

5

6

7

8

9

10: yellow

Monday

Tuesday

Wednesday

Thursday

Friday

Saturday: yellow

Sunday

The days are rectangular boxes next to each other. Saturday and Sunday are quite flat, and then Monday starts to rise. The week peaks at Wednesday and slopes back down to the weekend. The whole thing starts again each week.

January: white

February

March

April

May

June

July

August

September

October

November

December

Each month is a square shape, with the first six months of the year in a row along the top, and the second six months in a row along the bottom.

Once a year ends, I visualise going right back to the left hand start of the top row again. When people mention dates, seasons, events etc. to me, they ‘slot in’ to the month they are in.

Comments on Tilt Brush Video:  I would have to change a black background though, as every image that I see is light in colour. I've never thought about that before.
